# Supplementary material for: Men and sexual and reproductive healthcare in the Nordic countries: a scoping review
Source: BMJ Open. 2021 Sep 30;11(9):e052600. doi: 10.1136/bmjopen-2021-052600 (PMC8487177; doi:10.1136/bmjopen-2021-052600)
Supplement: Supplementary data [file bmjopen-2021-052600supp001.pdf]

Appendix 1: Search terms

1) Pubmed/Medline

Search	Search terms	Nr. Of articles
**#1<br>Sexual and reproductive health**	(Sexual Health[mesh] OR "Sexual health" OR Reproductive Health[mesh] OR "Reproductive health" OR<br>"Sexual and reproductive health" OR "Sexual and reproductive health and rights" OR SRHR OR<br><br>"sexual function" OR "sexual functions" OR "sexual dysfunction" OR "sexual dysfunctions" OR<br>erectile dysfunction[mesh] OR "erectile dysfunction" OR sexual satisfaction OR sex offenses[mesh] OR sexual violence OR gender-based violence[mesh] OR gender-based violence OR<br><br>family planning services[mesh] OR "Family Planning" OR contraceptive agents[mesh] OR contraceptive devices[mesh] OR "contraceptive" OR "contraceptives" OR Condom[mesh] OR condom OR condoms OR Infertility[mesh] OR infertility OR fertility[mesh] OR fertility OR prostatic neoplasms[mesh] OR "Prostate cancer" OR<br><br>Genital Diseases, Male[mesh] OR sexually transmitted diseases[mesh] OR "Sexually transmitted infections" OR STIs OR "Sexually transmitted diseases" OR STDs OR chlamydia[mesh] OR Chlamydia OR Gonorrhoea[mesh] OR gonorrhea OR gonorrhoeae OR Syphilis[mesh] OR Syphilis OR Trichomonas Infections[mesh] OR Trichomoniasis OR Herpes Genitalis[mesh] OR "herpes genitalis"[tiab] OR Papillomavirus Infections[mesh] OR "Papillomavirus" OR Condylomata Acuminata[mesh] OR "Genital warts" OR HIV Infections[mesh]) AND	1 034 552
**#2<br>men**	(men[mesh] OR Men OR man OR men's OR Male[mesh] OR male OR masculinity[mesh] OR masculinity OR Men's Health[mesh] OR Homosexuality, Male[mesh] OR "MSM" OR "men having sex with men"[TIAB] OR "men who have sex with men"[TIAB] OR "men who have sex with other men"[TIAB] OR<br>((transgender*[TIAB] OR transgender persons[MH] OR transsexual*[TIAB]) AND man) OR transman[TIAB] OR "trans men"[TIAB] OR transmen[TIAB]) AND	558 975
**#3<br>healthcare**	(Health Services Accessibility[mesh] OR Health Facilities[mesh] OR community health services[mesh] OR health services[mesh] OR health services research[mesh] OR delivery of health care[mesh] OR preventive health services[mesh] OR health services needs and demand[MH] OR quality of health care[majr:noexp] OR "Health care providers" OR<br><br>"Health care"[tiab] OR "Health services"[tiab] OR "Healthcare"[tiab] OR "health-care"[tiab] OR clinic[tiab] OR hospital[tiab] OR "primary care"[tiab]) AND	121 596
**#4 experiences**	(Professional-Patient Relations[mesh] OR Attitude of Health Personnel[mesh] OR Patient Satisfaction[mesh] OR Healthcare Disparities[mesh] OR patient acceptance of health care[MH] OR health care evaluation mechanisms[mesh] OR<br><br>"perception"[tiab] OR "perceptions"[tiab] OR "perceive"[tiab] OR "perceived"[tiab] OR "satisfaction"[tiab] OR "expectation"[tiab] OR "expectations"[tiab] OR "experience"[tiab] OR "experiences"[tiab] OR "evaluation"[tiab] OR "assessment"[tiab] OR "quality"[tiab] OR "trust"[tiab] OR "Shame"[tiab] OR "stigma"[tiab]) AND	93 209
**#5<br>Empirical studies**	(Epidemiologic Research Design[mesh] OR Qualitative Research[mesh] OR empirical research[mesh] OR<br>Surveys and Questionnaires[mesh] OR survey OR surveys OR questionnaires OR questionnaire OR Empirical OR Cross-Sectional Studies OR Cohort OR Case-Control OR Observational OR Registries OR analysis OR	79 428

	Clinical Trials OR Meta-Analysis OR “meta analysis” OR “systematic review” OR “scoping review” OR “literature review” OR “review of literature” OR<br><br>Qualitative OR “Grounded Theory” OR Interviews as Topic[mesh] OR “interviews” OR “interview” OR focus groups[mesh] OR “Focus group” OR “focus groups” OR themes[tiab]) AND	
**#6**<br>**Nordic countries**	(Scandinavian and Nordic Countries[mesh] OR Scandinavian[tiab] OR Denmark[tiab] OR Danish[tiab] OR Finland[tiab] OR Finnish[tiab] OR Norway[tiab] OR Norwegian[tiab] OR Sweden[tiab] OR Swedish[tiab] OR Iceland[tiab] OR Icelandic[tiab] OR Greenland[tiab])	1892

Search results 13th Mai 2020: 1892 articles

Published from 1st January 2010: 896 articles

2) Svemed+

(Sexual health[mesh] OR Reproductive health[mesh] OR sexually transmitted diseases[mesh] OR hiv infections[mesh] OR contraceptive agents[mesh] OR contraceptive devices[mesh] OR infertility[mesh] OR prostatic neoplasms[mesh] OR sex offences[mesh] OR erectile dysfunction[mesh]) AND
(men[mesh] OR male[mesh] OR masculinity[mesh]) AND
(Professional-Patient Relations[mesh] OR Attitude of Health Personnel[mesh] OR Patient Satisfaction[mesh] OR Healthcare[mesh] Disparities[mesh] OR patient acceptance of health care[mesh] OR health care evaluation mechanisms[mesh] OR Health Facilities[mesh] OR community health services[mesh] OR health services[mesh] OR preventive health services[mesh])

Search results 26th April 2020: 1696 articles

Published from 1st January 2010: 390 articles
