# Supplementary material for: Men and sexual and reproductive healthcare in the Nordic countries: a scoping review
Source: BMJ Open. 2021 Sep 30;11(9):e052600. doi: 10.1136/bmjopen-2021-052600 (PMC8487177; doi:10.1136/bmjopen-2021-052600)
Supplement: Supplementary data [file bmjopen-2021-052600supp002.pdf]

Appendix 2:

list of included articles on men's experiences in sexual and reproductive healthcare in the Nordic countries

Authors (year)	SRH subject	Country	Study design	Aim of the study	Discussed concepts
**Åhman et al. (2012)** [74]	1	Sweden	Qualitative (interviews) 17 m	To explore men's expectations of routine ultrasound and experiences when soft markers were discovered.	Information, policy, organization of care, waiting times, satisfaction, control, autonomy, Support, power and responsibility
**Andersson et al. (2012)** [37]	1	Sweden	Qualitative (interviews) 20 k, 8 m	To investigate parents' experiences and perceptions of group antenatal care in different antenatal clinics in Sweden	Knowledge about the services, continuity of care, users' gender, information, hcps' knowledge/competence, Engaging men
**Andersson et al. (2016)** [63]	1	Sweden	Quasi randomized study (survey) 627 m	To identify expectant fathers' expectations regarding the content of antenatal care during pregnancy and to examine associations between expectations and social factors	Information, regard, engaging men, users' age
**Andersson et al. (2017)** [77]	1	Sweden	Quasi randomized study (survey) 239 m	To compare the experiences of fathers with two different models of antenatal care, group based antenatal care and standard antenatal care	Information, satisfaction, support, autonomy
**Armuand et al. (2012)** [64]	5, cancer	Sweden	Cross-sectional (survey) 328 k, 156 m	To investigate cancer survivors' perception of fertility-related information and use of FP options in connection with cancer treatment during reproductive age. An additional aim was to investigate the relationships between receiving fertility-related information and sociodemographic factors, diagnosis, and a pretreatment desire for children.	Information, users' age
**Armuand et al. (2015)** [32]	5, cancer	Sweden	Qualitative (interviews) 11 k, 10 m	To investigate newly diagnosed cancer patients ' experiences of fertility-related communication and their reasoning about the risk of future infertility.	Information, satisfaction, autonomy, time constrain, No body's responsibility, vulnerability, masculinity
**Armuand et al. (2017)** [85]	5	Sweden	Qualitative (interviews) 15 m	To evaluate how transgender men experienced FP aimed at oocyte cryopreservation in a pilot program, which was developed within an established university hospital-based FP program.	Referral possibility, waiting times, vulnerability, masculinity, satisfaction
**Åsenhed et al. (2014)** [38]	1	Sweden	Qualitative (internet bloggs) 11 m	To identify and describe the process of fatherhood during the partner's pregnancy among expectant, first-time fathers.	Women centred care, engaging men, users' gender, willingness to talk
**Bäckström et al. (2011)** [42]	1	Sweden	Qualitative (interviews) 10 m	To explore how first-time fathers describe requested and received support during a normal birth.	Communication, trust, support, engaging men, autonomy, users' as person

Baroudi M, *et al. BMJ Open* 2022; 11:e052600. doi: 10.1136/bmjopen-2021-052600

**Berg (2013)** [51]	6	Norway	Cross-sectional (survey) 2011 m	To identify prevalence of, and factors that are associated with, HIV testing	Users' age, users' education, users' knowledge, knowledge about the services, users' attitude, users' sexuality, confidentiality, regard, holistic care
**Bergengren et al. (2018)** [67]	Cancer	Sweden	Register based cross-sectional (survey) 1 288 m	To investigate overall satisfaction with care (OSC) and factors associated with OSC among men with low-risk prostate cancer (PC)	Satisfaction, autonomy, information, hcps' profession, users' age, users' education, waiting times, health outcome
**Berglund et al. (2012)** [68]	Cancer	Sweden	Population based cohort 17 522 m	To examine possible associations between socioeconomic status, metastatic work-up, treatment and mortality in patients with high risk pca managed in Sweden	Users' SES, health outcome
**Bjornshagen et al. (2020)** [55]	6	Norway	Cross-sectional (survey) 849 m	To illustrate who might benefit from HIV self-testing, by describing the characteristics of MSM who took an interest in the GLHN pilot project	Confidentiality, anonymity, health seeking behaviours, users' sexuality, users' age
**Bodin et al. (2018)** [70]	5	Sweden	Randomised controlled study (structured interviews) 201 m	To evaluate if Reproductive Life Plan (RLP)-based counselling during a sexual health visit could increase men's fertility awareness	Users' knowledge, information, satisfaction
**Brannstrom et al. (2016)** [10]	6	Sweden	Cross-sectional (survey) 372 m, 203 w	To identify factors in HIV-infected patients and the health care system which contribute to late diagnosis.	Users' age, users' ethnicity, health outcome, users' sexuality, users' gender
**Christianson et al. (2013)** [49]	6	Sweden	Qualitative (interviews) 20 m	To investigate how to prevent transmission of HIV and CT from a gender perspective by exploring whether screening of men during pregnancy may be an innovative way to reach men, to increase detection, and to avoid the present gendered responsibility.	Risk perception, engaging men, women centred healthcare, information, knowledge about the services, social norms, No body's responsibility, users' attitude
**Christianson et al. (2017)** [13]	6	Sweden	Qualitative (interviews) 20 m	To discursively explore expectant fathers' perceptions of chlamydia and HIV, and their masculinity constructions about testing, and explored how they talked about their potential resistance towards testing and their pre-test emotions.	Vulnerability, masculinity, health seeking behaviour, users' attitude/feelings
**Erlandsson and Häggström-Nordin (2010)** [86]	1, 2	Sweden	Qualitative (interviews) 15 m	To capture fathers' conceptions of parental education topics, illuminated by their experiences as primary caregiver of their child immediately following birth.	Role of men, engaging men, social norms
**Fabian et al. (2015)** [44]	1, 2	Sweden	Qualitative (interviews) 26 midwives	To explore antenatal care midwives' experiences and thoughts about the parental class activities provided during pregnancy.	Holistic care, clinical training, hcps' competence, Users' social capital, engaging men, users' gender

**Fridriksson et al. (2012)** [66]	2, cancer	Sweden	Register based cross-sectional (survey)<br>1 621 m	To assess the proportion of men subsequently diagnosed with prostate cancer who had received information prior to blood draw	Information, knowledge about the services
**Grandahl and Small (2019)** [17]	Other	Sweden	Qualitative (interviews)<br>22 midwives	To explore the thoughts and experiences of midwives working in the primary care setting concerning their preventive work for men's sexual and reproductive health and rights	No body's responsibility, hcps' attitude, Clinical training, hcps' knowledge, health seeking behaviour, organization of care, time constrains, women centred care, engaging men
**Grandahl et al. (2109)** [48]	6	Sweden	Qualitative (interviews) 33 m	To investigates boys' awareness and thoughts about human papillomavirus (HPV) and HPV vaccination, perceived benefits of vaccinating men, information sources and intention to be vaccinated against HPV	Users' attitude, willingness to participate, risk perception, information
**Hasman et al. (2014)** [46]	1	Denmark	Qualitative (interviews)<br>10 m	To describe how fathers experienced childbirth when nonprogressive labour occurred and augmentation was established.	Information, engaging men, role of men, control, hcps' competence, , security
**Herder and Agardh (2019)** [34]	6	Sweden	Qualitative (interviews)<br>10 m	To explore experiences and perceptions regarding communication about infectiousness and the rules of conduct with clinical staff at HIV clinics among MSM living with HIV in Sweden	Hcps' attitude, regard, trust, hcps' knowledge, security, , information
**Hildingsson and Sjöling (2011)** [60]	1	Sweden	Prospective longitudinal study (survey)<br>655 m	To describe personal and professional sources of support used by prospective and new fathers and to study factors associated with fathers having no support from anyone in mid-pregnancy.	Support, engaging men, users' social capital, willingness to participate, satisfaction
**Hildingsson et al. (2011)** [62]	1	Sweden	Cross-sectional (survey)<br>595 m	To identify the proportion of fathers having a positive experience of a normal birth and to explore factors related to midwifery care that were associated with a positive experience.	Satisfaction, support, information, users' age, users' ethnicity, users' education
**Holter et al. (2014)** [71]	5	Sweden	Cross-sectional (survey)<br>292 m,363 k	To investigate whether men and women differ in their evaluations of the importance of different aspects of quality of care, when measured by the use of the validated QPP-IVF instrument and to investigate if any baseline characteristics influenced the scores of subjective importance	Continuity of care, information, satisfaction
**Holter et al. (2017)** [83]	5	Sweden	Cross-sectional (survey)<br>268 IVF staff, 1435 m, 1863 k	To compare IVF healthcare professionals' estimates with patients' actual experiences of patient-centered quality of care measured with the QPP-IVF questionnaire, and investigate if certain factors influenced the IVF professionals' perceptions and IVF patients' experience of quality of care.	Satisfaction, continuity of care, autonomy, easy access, users' education, public vs. Private

Baroudi M, *et al. BMJ Open* 2022; 11:e052600. doi: 10.1136/bmjopen-2021-052600

**Hoyos et al. (2018)** [58]	6	Multinational	Cross-sectional (survey) 8 226 m (397 Danish)	To describe the knowledge about the existence as well as actual and potential use of self-sampling testing and to assess the acceptability of different result communication methods as well as the preferred sampling method among MSM recruited online in eight European countries.	Knowledge about the services, acceptability, communication
**Johansson and Hildingsson (2013)** [78]	1	Sweden	Cross-sectional (survey) 827 m	To explore Swedish fathers' intrapartum care quality experiences, with a specific focus on care deficiencies in relation to birth mode. A secondary aim was to explore which issues of quality that contributed most to dissatisfaction with the overall assessment of the care.	Satisfaction, support, autonomy, nature of the problem, information
**Johansson and Thies-Lagergren (2015)** [33]	1	Sweden	Mixed methods (survey, free texts) 221 m	To investigate how maternal birth position during second stage of labour may influence fathers' experience of childbirth	Satisfaction, nature of the problem, power, security, engaging men, support, willingness to participate, hcps' attitude, information, hcps' knowledge/competence, continuity of care, trust
**Johansson et al. (2011)** [15]	5	Sweden	Qualitative (interviews) 8 m	To describe men's experiences of obstructive azoospermia infertility	Vulnerability, masculinity, women centred care, engaging men
**Johansson et al. (2012)** [41]	1	Sweden	Mixed methods (survey, free texts) 827 m	To explore Swedish fathers' birth experiences, and factors associated with a less-positive birth experience.	Satisfaction, nature of the problem, users' education, hcps' competence, support, information, regard, autonomy, engaging men
**Johansson et al. (2013)** [75]	1	Sweden	Qualitative (interviews) 22 m	To describe and explore fathers' experiences of their partner's caesarean section birth.	Nature of the problem, information, hcps' Attitude, support, satisfaction, hcps' competence, control
**Johansson et al. (2014)** [82]	1	Sweden	Qualitative (interviews) 21 m	To explore and describe Swedish fathers' beliefs and attitudes around the decision for a caesarean section.	Shared responsibility, trust, information, nature of the problem
**Johnsen et al. (2017)** [39]	1	Multinational	Qualitative (interviews) 31 m Sweden, 8 m Denmark, 5 m Finland	To illuminate expectant first-time fathers' experiences of participation during pregnancy in three Nordic countries.	Willingness to participate, engaging men, social norms, responsibility
**Jungmarker et al. (2010)** [76]	1	Sweden	Cohort study (survey) 827 m	To describe expectant fathers' experiences of and involvement in prenatal care in Sweden.	Willingness to participate, satisfaction, holistic care, information, support, engaging men
**Kero et al. (2010)** [73]	7	Sweden	Qualitative (interviews) 23 couples	To gain knowledge about the male partner's experience of being present during an induced home abortion	Willingness to participate, autonomy, information, engaging men

**Klaeson et al. (2013)** [28]	8, cancer	Sweden	Qualitative (fgds) 19 m	To explore how men diagnosed with prostate cancer before the age of 65 years, in all stages, experienced and talked about changes in their sexuality due to cancer as a subgroup in the society	Vulnerability, masculinity, nature of the problem, social norms, Willingness to talk, hcps' gender, continuity of care, information, support
**Klaeson et al. (2017)** [16]	Other	Sweden	Qualitative (interviews) 9 nurses	To illuminate nurses' experiences and opportunities to discuss sexual health with patients in primary health care	Social norms, hcps' attitude, clinical training, organization of care, no body's responsibility, users' gender, clinical training, holistic care, users' age
**Koert et al. (2019)** [43]	1	Denmark	Qualitative (interviews) 11 couples	What do couples referred to or attending a Recurrent Pregnancy Loss clinic believe they need in terms of treatment, support and follow up?	Holistic care, regard, engaging men , information, continuity of care, referral possibility, organization of care
**Linnarsson et al. (2013)** [87]	4	Sweden	Cross-sectional (survey) 46 emergency departments	To describe the preparedness to provide care for victims of violence and their families in emergency departments (eds) in Sweden.	Women centred care, policy, organization of care, clinical training
**Makenzius et al. (2012)** [84]	7	Sweden	Cross-sectional (survey) 590 m, 798 k	To investigate satisfaction with abortion care among women and their male partners, and to identify factors associated with high overall care satisfaction	Satisfaction, regard, information, willingness to participate
**Makenzius et al. (2013)** [53]	7	Sweden	Qualitative (interviews) 24 k, 13 m	To explore women's and men's experiences and needs in relation to an induced medical abortion that involves carrying out the final treatment at home and to elicit their views on contraception and prevention of future unwanted pregnancies.	Autonomy, responsibility, continuity of care, privacy, control, information, regard, satisfaction, women centred care
**Marrone et al. (2016)** [54]	6	Sweden	Register based cross-sectional (survey) 1896 m, 950 k	To evaluate the Health Questionnaire and identify the main determinants of adherence	Satisfaction, autonomy, users' gender, users' ethnicity, health outcome
**Micaux Obol et al. (2017)** [31]	5, cancer	Sweden	Nationwide cross-sectional (survey) 329 oncologists and haematologists	To investigate the practice behaviors, attitudes, confidence in knowledge and perceived barriers to discussing fertility issues among physicians in cancer care and to identify factors related to physicians' practice behaviors regarding discussions about treatment-related fertility risks with female and male patients of reproductive age.	Information, users' age, no body's responsibility, referral possibility, workload, organization of care, hcps' knowledge
**Mikkelsen et al. (2013)** [27]	5	Denmark	Cross-sectional (survey) 210 m	To gain further knowledge about the experiences of infertile men for whom intracytoplasmic sperm injection treatment was the only way to establish fatherhood and to explore the psychological needs of the infertile man, focusing on communication in the clinic	Vulnerability, masculinity, engaging men, information , hcps' gender

**Oster et al. (2013)** [35]	Cancer	Sweden	Qualitative (conversational support group sessions) 9 m	To describe the shared experiences in a conversational support group of men with prostate cancer during a course of radiotherapy.	Satisfaction, nature of the problem, autonomy, waiting times, information, hcps' attitude, support, regard
**Persson et al. (2012)** [47]	1	Sweden	Qualitative (fgds, interviews) 20 m	To explore and describe factors, which influence fathers' sense of security during the first postnatal week.	Willingness to participate, information, security, hcps' knowledge/competence, organization of care, engaging men, support, regard, follow up
**Persson et al. (2016)** [50]	6	Sweden	Cross-sectional (survey) 2 373 m	To explore motivators and barriers to HIV testing and to assess factors associated with testing among MSM in the era of ART	Risk perception, users' knowledge, confidentiality, knowledge about the services, users' age, users' ethnicity, users' education
**Premberg et al. (2011)** [45]	1	Sweden	Qualitative (re-enactment interviews) 10 m	To describe fathers' experiences during childbirth	Information, willingness to participate, engaging men, support, autonomy
**Qvarnstrom and Oscarsson (2015)** [72]	6	Sweden	Cross-sectional (survey) 656 m	To describe experiences of and attitudes towards HIV/ STI prevention efforts prior to travel abroad among MSM and to investigate the kinds of prevention efforts that are desirable.	Availability of services, information, information
**Qvist et al. (2014)** [56]	6	Denmark	Cross-sectional (survey) 1 clinic	To evaluate a community based human immunodeficiency virus (HIV) testing program for its capacity to reach men who have sex with men (MSM) and successfully refer HIV-positive patients to treatment.	Easy access, follow up, referral possibility
**Rasmusson et al. (2013)** [65]	8, cancer	Sweden	Cross-sectional (survey) 54 k, 51 m	To investigate information about sexual effects of cancer on patients irrespective of age and diagnosis in terms of fertility, sexual desire and sexual function.	Information, users' gender, users' age
**Schildmeijer et al. (2019)** [79]	Cancer	Sweden	Qualitative (interviews) 14 m	1. Explore and describe how patients diagnosed with prostate cancer experience their journey through cancer care, by visualizing a typical patient journey and juxtaposing it with the SCP, and 2. Identify the patients' needs for support during the journey.	Information, waiting times, control, vulnerability, masculinity
**Schmidt et al. (2013)** [57]	6	Multinational	Cross-sectional (survey) 52430 m	To compare the performance of STI services used by MSM.	Knowledge about the services
**Schytt and Bergstrom (2014)** [61]	1	Sweden	Randomized controlled study (survey) 777 m	To investigate first-time fathers' expectations and experiences of childbirth and satisfaction with care in relation to paternal age.	Users' age, satisfaction, hcps' competence, support, trust

**Simmons et al. (2016)** [29]	4	Sweden	Qualitative (interviews) 12 m	To develop a theoretical model concerning male victims' processes of disclosing experiences of victimisation to healthcare professionals in Sweden.	Holistic care, fear, trust, confidentiality, masculinity, users' gender, hcps' gender, social norms, gender norms, support, regard
**Sollesnes (2010)** [5]	Other	Norway	Qualitative (fgds) 22 k, 10 m	To obtain insight to factors that can influence adolescent males and females' use of adolescent health clinics	Women centred care, help seeking behaviour, knowledge about the services, confidentiality, regard (patient as person)
**Stromdahl et al. (2017)** [52]	6	Sweden	Cross-sectional (survey) 244 m	To examine HIV-testing prevalence and uptake of HIV prevention interventions including different HIV-testing options among foreign-born MSM living in Sweden	Users' ethnicity, knowledge about the services, risk perception, easy access, anonymity, confidentiality
**Stromdahl et al. (2019)** [59]	6	Sweden	Cross-sectional (survey) 595 m	To evaluate the uptake of Testpoint (whether Testpoint achieved its aim of reaching MSM and trans persons, with a special focus on young and foreign born MSM)	Health seeking behaviour, users' ethnicity, users' age, knowledge about the services
**Sylvest et al. (2016)** [36]	5	Denmark	Qualitative (interviews) 10 m	To explore experience, expectations, needs, and assessment of fertility care among men with severe male-factor infertility.	Information, communication, waiting times, regard (patient as a person), willingness to participate, engaging men, holistic care
**Sylvest et al. (2018)** [69]	5	Denmark	Qualitative (interviews) 21 m	To explore men's expectations and experiences of fertility assessment and counseling through qualitative interviews conducted immediately before and some weeks after fertility counseling	Information, power, satisfaction, regard, masculinity, vulnerability
**Thies-Lagergren and Johansson (2019)** [81]	1	Sweden	Cross-sectional (survey) 209 couples	To describe and evaluate uniformity in couples' birth experience and experience of the quality of intrapartum midwifery care	Satisfaction, control, support,
**Vik and Brekke (2017)** [30]	Other	Norway	Cross-sectional (survey) 22 general practitioners 1 117 consultations	To shed some light upon how frequently and how Norwegian gps deal with concerns related to sexuality among their patients.	Prevalence of sexual problems presented to GP, hcps' competence, satisfaction
**Wibe et al. (2012)** [80]	2, cancer	Norway	Qualitative (online messages, interviews) 12 m	To explore how an online patient-nurse communication (OPNC) service meets the information needs of men with newly diagnosed testicular cancer	Information, support, control, communication, Waiting times
**Widarsson et al. (2012)** [14]	1	Sweden	Qualitative (fgds, interviews) 22 k, 10 m	To describe expectant mothers' and fathers' perceived needs of support during pregnancy	Holistic care, satisfaction, engaging men, women centred care, information
**Widarsson et al. (2015)** [40]	1	Sweden	Qualitative (fgds, interviews) 20 k, 10 m	To describe the perspectives of expectant mothers and fathers on fathers' involvement during pregnancy	Willingness to participate, engaging men, information, satisfaction

Baroudi M, *et al. BMJ Open* 2022; 11:e052600. doi: 10.1136/bmjopen-2021-052600

1. Antenatal, intrapartum and postnatal care
2. Comprehensive education and information
3. Contraception counselling and provision
4. Gender-based violence prevention, support and care
5. Fertility care
6. Prevention and control of HIV and other sexually transmissible infections
7. Safe abortion care
8. Sexual function and psychosexual counselling

Baroudi M, *et al. BMJ Open* 2022; 11:e052600. doi: 10.1136/bmjopen-2021-052600
