# Supplementary material for: Men and sexual and reproductive healthcare in the Nordic countries: a scoping review
Source: BMJ Open. 2021 Sep 30;11(9):e052600. doi: 10.1136/bmjopen-2021-052600 (PMC8487177; doi:10.1136/bmjopen-2021-052600)
Supplement: Author's manuscript [file bmjopen-2021-052600.draft_revisions.pdf]

BMJ Open is committed to open peer review. As part of this commitment we make the peer review history of every article we publish publicly available.

When an article is published we post the peer reviewers' comments and the authors' responses online. We also post the versions of the paper that were used during peer review. These are the versions that the peer review comments apply to.

The versions of the paper that follow are the versions that were submitted during the peer review process. They are not the versions of record or the final published versions. They should not be cited or distributed as the published version of this manuscript.

BMJ Open is an open access journal and the full, final, typeset and author-corrected version of record of the manuscript is available on our site with no access controls, subscription charges or pay-per-view fees (http://bmjopen.bmj.com).

If you have any questions on BMJ Open's open peer review process please email info.bmjopen@bmj.com

BMJ Open

Men and sexual and reproductive healthcare in the Nordic countries: a scoping review

Journal:	*BMJ Open*
Manuscript ID	bmjopen-2021-052600
Article Type:	Original research
Date Submitted by the Author:	20-Apr-2021
Complete List of Authors:	Baroudi, Mazen; Umeå Universitet Medicinska fakulteten, Epidemiology and Global Health<br>Stoor, Jon Petter; Umeå Universitet Medicinska fakulteten, Department of Epidemiology and Global Health; UiT The Arctic University of Norway, Centre for Sami Health Research, Department of Community Medicine<br>Blåhed, Hanna; Umeå Universitet Medicinska fakulteten, Department of Epidemiology and Global Health<br>Edin, Kerstin; Umeå Universitet Medicinska fakulteten, Department of Epidemiology and Global Health<br>Hurtig, Anna-Karin ; Umeå Universitet Medicinska fakulteten, Department of Epidemiology and Global Health
Keywords:	PUBLIC HEALTH, HIV & AIDS < INFECTIOUS DISEASES, Sexual dysfunction < UROLOGY

SCHOLARONE™
Manuscripts

I, the Submitting Author has the right to grant and does grant on behalf of all authors of the Work (as defined in the below author licence), an exclusive licence and/or a non-exclusive licence for contributions from authors who are: i) UK Crown employees; ii) where BMJ has agreed a CC-BY licence shall apply, and/or iii) in accordance with the terms applicable for US Federal Government officers or employees acting as part of their official duties; on a worldwide, perpetual, irrevocable, royalty-free basis to BMJ Publishing Group Ltd ("BMJ") its licensees and where the relevant Journal is co-owned by BMJ to the co-owners of the Journal, to publish the Work in this journal and any other BMJ products and to exploit all rights, as set out in our licence.

The Submitting Author accepts and understands that any supply made under these terms is made by BMJ to the Submitting Author unless you are acting as an employee on behalf of your employer or a postgraduate student of an affiliated institution which is paying any applicable article publishing charge ("APC") for Open Access articles. Where the Submitting Author wishes to make the Work available on an Open Access basis (and intends to pay the relevant APC), the terms of reuse of such Open Access shall be governed by a Creative Commons licence – details of these licences and which Creative Commons licence will apply to this Work are set out in our licence referred to above.

Other than as permitted in any relevant BMJ Author's Self Archiving Policies, I confirm this Work has not been accepted for publication elsewhere, is not being considered for publication elsewhere and does not duplicate material already published. I confirm all authors consent to publication of this Work and authorise the granting of this licence.

For peer review only

Men and sexual and reproductive healthcare in the Nordic countries: a scoping review

Mazen Baroudi* mazen.baroudi@umu.se

+46– 90 786 75 54 Department of Epidemiology and Global Health, Umeå University, 901 87 UMEÅ

Jon Petter Stoor, petter.stoor@umu.se

Department of Epidemiology and Global Health, Umeå University, Umeå

Centre for Sami Health Research, Department of Community Medicine, UiT – the Arctic University of Norway, Tromsø, Norway

Hanna Blåhed hanna.blahed@umu.se

Department of Epidemiology and Global Health, Umeå University, Umeå

Kerstin Edin kerstin.edin@umu.se

Department of Epidemiology and Global Health, Umeå University, Umeå

Anna-Karin Hurtig anna-karin.hurtig@umu.se

Department of Epidemiology and Global Health, Umeå University, Umeå

*corresponding author

For peer review only

Men and sexual and reproductive healthcare in the Nordic countries: a scoping review

Abstract

Context

Men generally seek healthcare less often than women and, other than traditional gender norms, less is known about the explanation. The aim was to identify knowledge gaps and factors influencing men regarding sexual and reproductive healthcare (SRHC) in the Nordic countries.

Methods

We searched PubMed and SveMed+ for peer-reviewed articles published between 2010 and 2020. The analyses identified factors influencing men's experiences of and access to SRHC.

Results

The majority of the 68 articles included focused on pregnancy, birth, infertility, and sexually transmitted infections including HIV. During pregnancy and childbirth, men were treated as accompanying partners rather than individuals with their own needs. The knowledge and attitudes of healthcare providers were crucial for their ability to provide SRHC and for the experiences of men. Organizational obstacles, such as women-centred SRHC and no assigned profession, hindered men's access to SRHC. Lastly, the literature rarely discussed the impact of health policies on men's access to SRHC.

Conclusions

The identified knowledge gap indicates the necessity of the improved health and medical education of healthcare providers, as well as of health system interventions.

Keywords

Sexual and reproductive healthcare, men, experience, Nordic countries, gender norms, right to healthcare

Strengths and limitations of this study

- This review is the first to examine the experiences of men in SRHC in the Nordic countries.
- We used of a Nordic-specific database without restriction to language
- Search was restricted to two databases but complemented with manually screening the reference lists of the identified literature
- The broad nature of the field and the wide variety of terms related to sexual and reproductive health make it difficult to assure the inclusion of all relevant literature.

For peer review only

For peer review only - http://bmjopen.bmj.com/site/about/guidelines.xhtml

- We implicitly treated the Nordic countries as essentially similar, which might obscure important differences between or within countries.

For peer review only

For peer review only - http://bmjopen.bmj.com/site/about/guidelines.xhtml

Introduction

Men generally seek healthcare, especially primary healthcare, to a lower degree than women, and this also applies to sexual and reproductive healthcare (SRHC).[1,2] For example, 23% of over 40 year-old men in Europe reported sexual dysfunction but only one-quarter of them sought healthcare,[3] and similar results have been reported in Sweden.[4] Also, studies from Sweden and Norway have indicated that youth clinics are perceived as "women clinics". Therefore, fewer men seek these services compared to young women.[5,6] Additionally, men test themselves for sexually transmitted diseases to a lower extent compared to women.[7,8] However, various groups of men might have different health seeking behaviours. For example, men with high socioeconomic status[1,9] and men who have sex with men (MSM) seek SRHC more often.[10,11]

Traditional gender norms might urge men to be independent, strong and invulnerable and also hinder them from acknowledging having problems, creating a barrier to seeking healthcare.[12] In particular, admitting sexual health problems might imply more vulnerability for men, thus decreasing the likelihood of seeking healthcare.[5,13] Even though gender norms play an important role in men's health seeking behaviours, it cannot alone explain the lower utilization of SRHC. Men who eventually sought SRHC did not get the help they expected. For example, more than half of men who sought help related to sexual function in Sweden reported not getting enough support.[4] Furthermore, men often felt excluded in healthcare related to infertility and pregnancy.[14,15] This mirrors the lack of response of the health system to men's needs that can be related to healthcare organization and delivery,[9] including no support or guidelines for health professionals to promote men's SRH.[16] Additionally, health and medical education in Sweden, as an example, does not have enough focus on men's SRH.[16,17]

The Nordic countries are among the best in the world in the available international gender equality statistics.[18] Since gender inequality affects women's sexual and reproductive health (SRH) to a larger degree compared to men,[19,20] there is a greater focus on women's rights to SRH. Men's SRH does not get the same attention in practice and little is known about men's SRH in the Nordic countries.[21] The available literature mainly focuses on gender norms and masculinities and its link to health seeking behaviours and risk taking, while much less is known on how men are experiencing SRHC.[9,21-23]

Aim

The aim of this scoping review was to identify knowledge gaps and factors influencing men regarding SRHC in the Nordic countries during the period between 2010 and 2020.

For peer review only - http://bmjopen.bmj.com/site/about/guidelines.xhtml

Method

Search strategies and selection criteria

A structured search of the literature was conducted using two databases, PubMed and SveMed+ (a Scandinavian database). Search terms included sexual and reproductive health, men, healthcare, experiences, and Nordic countries (see Appendix 1 for detailed search terms). The following eligibility criteria were used: (1) peer-reviewed empirical studies, all study designs were considered; (2) published between January 2010 and May 2020; (3) assessing men's experiences in SRHC or perspectives of HCPs on men's SRHC; and (4) conducted in the Nordic countries.

The initial search gave 1286 articles (896 from PubMed and 390 from SveMed+). After screening the titles and abstracts, 108 articles were read in full, and after being judged for their eligibility, 44 articles remained. An additional 24 papers were identified through the reference lists of these papers, resulting in 68 papers included in this scoping review (Figure 1). The articles were judged for eligibility by the first author, but when uncertainties arose, two co-authors read and judged the articles for eligibility separately. The three researchers then discussed the articles and decided unanimously on the inclusion/exclusion of these articles.

Data extraction and synthesis

The identified articles were mapped using the World Health Organization (WHO) framework for operationalizing SRH,[24] and the result parts of each article were extracted and coded using sensitizing concepts of healthcare experiences (Appendix 2). Thereafter, the results were synthesized using a theoretical framework, adapted from Kilbourne et al., which provides health service research perspectives on understanding health and healthcare disparities.[25]

Figure 1: PRISMA flow chart on search results of men's experiences in sexual and reproductive healthcare in the Nordic countries.

For peer review only - http://bmjopen.bmj.com/site/about/guidelines.xhtml

Results

Description of the identified studies

Despite not restricting the language of the studies, all the 68 studies included were in English. The absolute majority of the studies were conducted in Sweden (54 articles), while six studies were conducted in Denmark, five in Norway and three in more than one country. No studies were identified from Iceland or Greenland.

Half of the studies (34) adopted a qualitative design, 32 studies a quantitative design and two studies a mixed methods design. Most of the studies (61 articles) were about men's perspectives of SRHC, while only seven studies covered the perspectives of healthcare providers (HCPs). Of the studies dealing with men's perspectives, 16 studies assessed women's perspectives together with that of men. Apart from two articles about the experiences of transgender men, the articles did not mention gender identities. Most of the papers dealing with men's perspectives referred to the overall experience of healthcare and healthcare staff in general. Of the 28 papers referring to specific primary healthcare providers, 14 mentioned midwives, eight mentioned physicians and six mentioned nurses.

SRH topics were grouped with help of the WHO framework for operationalizing sexual health and its linkages to reproductive health.[24] The framework demonstrates the interlinked nature between sexual health and reproductive health, yet clearly distinguish topics for intervention and research in both sexual health and reproductive health (Figure 2). Besides the eight topics from this framework, SRH cancers were also added, while the remaining studies with no one topic of focus were grouped under "other".

More than one-third of the papers were about the experiences of fathers/expectant fathers during antenatal, intrapartum and postnatal care (25 papers, including 12 about antenatal care and 11 about intrapartum care), while 15 papers dealt with sexually transmitted infections (STIs), mainly HIV (12 papers) and MSM (nine papers). We found 11 papers concerning men's experiences in infertility care (three of them were related to infertility among cancer patients) and eleven papers in cancer care. We also found four studies dealing with sexual education and information (two of them related to cancer and the other two related to antenatal care), three studies about abortion care, two studies about sexual violence and two studies about sexual functioning and counselling (both related to cancer patients). We found no study dealing with the provision of men's contraceptive counselling (Figure 3).

Figure 2: Framework for operationalizing sexual health and its linkages to reproductive health (from "Sexual health and its linkages to reproductive health: an operational approach").[24] The intertwined blue and orange ribbons represent sexual health and reproductive health, respectively.

Figure 3: Men's experiences in sexual and reproductive healthcare in the Nordic countries. Number of studies identified grouped by sexual and reproductive health topics.

Theoretical framework for analysis

The identified literature dealt with men's experiences in SRHC from various perspectives and can be organized in the framework adapted from Kilbourne et al.[25] The factors influencing men's

For peer review only - http://bmjopen.bmj.com/site/about/guidelines.xhtml

experiences are divided into (i) individual, including healthcare providers and users; (ii) interpersonal, which deals with the healthcare encounter and contact circumstances; (iii) organizational, which deals with healthcare system factors; and (iv) the larger influence of the community and public policies (Figure 4).

Figure 4: Theoretical framework for analysis of men's experiences in sexual and reproductive healthcare, adapted from Kilbourne et al.[25]

1. Healthcare providers' factors

The literature described on how factors related to HCPs, such as sex, attitude, knowledge and competence, affect the HCP-user relationship and experiences of men in healthcare. For example, female HCPs did not prevent men from talking about their concerns regarding infertility.[26] Similarly, men diagnosed with prostate cancer wished to talk about sexuality with a mature knowledgeable HCP, without considering their sex.[27] Furthermore, disclosing victimization to female HCPs as compared to male HCPs was claimed to be easier for some men.[28]

1.1. Varied levels of knowledge, competing demands and differing attitudes

Lacking knowledge about men's SRH was expressed by various HCP professions and were associated with less ability to deal with men's SRH consultations. For example, nurses perceived that their lack of knowledge was influencing their preparedness to provide sexual health consultations for men.[16] Midwives also expressed their limited knowledge about male SRH, which was considered essential if inviting men for SRH consultations.[17] Additionally, less experienced physicians (young and/or under training) felt uncomfortable dealing with sexual health consultations.[29] Additionally, the competing demands in the form of high workload and limited time hindered HCPs from discussing SRH with men.[16,30,31]

Differing attitudes towards health seeking behaviours of men were found. While most HCPs were described as having positive attitudes, being friendly, sensitive and supportive,[32-35] some were still perceived as harsh and nonresponsive.[32,33] These negative attitudes were sometimes perceived by men as discrimination based on their sex, which hindered them, for example, from disclosing victimization.[28]

1.2. The view of men in reproductive healthcare services, an accompanying partner or an individual?

Even though HCPs in reproductive healthcare services usually deal with couples having a common reason for visiting healthcare, in most cases, they have primarily communicated with the women.[26,32,36,37] Women were in focus during infertility treatment, pregnancy and birth, leading men to feel neglected, invisible and superfluous during the visits.[14,15,38,39] The lack of interest in listening to or interacting with men also hindered their involvement in supporting their partners, for example, when giving birth.[40,41]

The lack of focus on men might be explained by time constraints and no time being allocated to men's concerns during visits.[17] Anyhow, the attitudes and behaviours of HCPs generally made a difference in men's perception of their involvement or lack of involvement in healthcare.[38] Couples highlighted the need to treat partners on equal terms and to focus on them as a unit rather than solely on the women,[14,15,26,36] and they expected communication as inclusive with both partners.[35] Men also expressed that HCPs should welcome them to more active involvement during birth and

For peer review only - http://bmjopen.bmj.com/site/about/guidelines.xhtml

For peer review only

support their role as expectant fathers. HCPs should acknowledge men's needs and give them the opportunity to talk about their concerns.[14,26,32,42]

Examples of good practices involving men in reproductive healthcare are also mentioned in the literature. One example was participatory parental classes or separate parental classes for men and women dealing with men's concerns related to pregnancy and birth, which helped men to take part and to feel involved.[39,43] Another example was assigning tasks and continuously informing men during labour and allowing the father to stay at the hospital after the baby is born. These practices gave men a feeling of being important and recognized, hence receiving needed support.[41,44-46]

2. Healthcare users' factors

The literature described users' factors that influence their experiences in healthcare. This included men's socioeconomic situation, including education, age, knowledge and attitude. For example, the ages of users were discussed in relation to the ages of HCPs; nurses were more comfortable talking about sexuality with younger men as compared to men of their own age or older.[16] Young men, in comparison to young women, were pointed out as being less acquainted with youth clinics or where else to seek SRHC.[5]

Healthcare users' factors are discussed in the literature mainly in three SRH subject areas, namely, prevention and control of HIV and other STIs, antenatal/ intrapartum care and cancer care, which is elaborated on below.

2.1. Prevention and control of HIV and other STIs

Most of the literature focused on HIV testing and treatment and the sociodemographic factors of users related to it. See Box 1 for more details about the factors discussed in the literature.

The literature relating to other STIs (besides HIV) was limited to the attitudes of school boys toward HPV vaccination and attitudes of men toward STI testing during the pregnancies of their partners. School boys had a positive attitude with regard to participation in HPV vaccinations; they stated that vaccinating only girls is unfair. Even though they had a positive attitude to share the responsibility of STI prevention, boys rarely used condoms, especially if they knew their sex partner in advance.[47] Men's attitudes toward STI testing during pregnancy were diverse. Some men perceived the test as an "infidelity check" that is sensitive and can risk the relationship, while others perceived it as a safety measure that should be "routine" during pregnancy.[13,48]

For peer review only - http://bmjopen.bmj.com/site/about/guidelines.xhtml

Box 1: **The sociodemographic factors of users in relation to HIV testing and treatment**

- **Age**: Younger age was reported to be associated with higher HIV testing among men who have sex with men (MSM)[49,50] and earlier diagnosis in the general population.[10]
- **Education**: A lower level of education was associated with less testing for HIV in the general population,[50] but not among MSM.[49]
- **Country of birth**: Studies showed that country of birth was not associated with lower HIV testing among MSM.[49,51] However, two-thirds of the foreign-born HIV patients had not been tested for HIV at migration to Sweden.[10,52] Therefore, foreign-born men were more likely to be diagnosed late (65% of foreign-born compared to 43% of Swedish-born) and less likely to optimally adhere to HIV treatment.[10,53]
- **Sexuality**: Since HIV testing was perceived as implicitly implying same sex sexual relations, non-disclosing MSM were more likely to have been never tested for HIV.[50,54] However, MSM were less likely to be diagnosed late (40% of MSM compared to 67% of heterosexual patients) and less likely to optimally adhere to HIV treatment.[10,53]
- **Knowledge**: Men's knowledge about HIV transmission was associated with never being tested for HIV among MSM and the general population.[49,50] Never being tested for HIV was also associated with not knowing if the tests were free or affordable[50,55] and lack of knowledge about HIV testing services.[44,49,51] For example, only one-fourth of MSM knew about home sampling (Internet ordered tests),[56] and around 40% have never heard of the Testpoints programme (peer-led testing performed in MSM clubs, among other places).[57]
- **Risk perception**: The perception of having a very low risk of contracting STIs, including HIV, was highly associated with never being tested for HIV or STIs.[3,7,48,51]

2.2. Antenatal and intrapartum care

The literature discussed men's socioeconomic characteristics, knowledge and experiences in antenatal and intrapartum healthcare. Lack of knowledge about antenatal services, such as antenatal classes, was common among men; they usually had not heard about the service before but received information from their partners.[36] Men who had no social support from family and friends during the pregnancies of their partners were more dissatisfied with antenatal care and less likely to attend parental classes.[58] Studies found younger age and higher education level were associated with lower satisfaction with the overall birth experience,[40,59] while no such association was reported in relation to men's country of birth.[60] Additionally, younger men as compared to older men, perceived midwives as less supportive, less attentive and as not inspiring confidence.[59] These differences might be explained by younger men having higher expectations.[61]

2.3. Cancer care and SRH

The literature explored the factors of users related to cancer care, especially the effects of cancer treatment on fertility and sexuality. The majority of physicians claimed that they discussed the impact of cancer treatment on fertility if the patient was at reproductive age. However, one-third of the physicians did not do this regularly.[30,62] Around half of men in the 41–60 years old age group claimed that they had not received enough information about the effects of cancer treatment on sexual desire, sexual function and fertility.[63]

For peer review only - http://bmjopen.bmj.com/site/about/guidelines.xhtml

Similar to other SRH services, lack of knowledge about the services was common, with only around one-fifth of men knowing about the PSA test for prostate cancer screening before testing.[64] Studies showed no associations between age and the overall satisfaction with cancer care, while a higher level of education was associated with lower overall satisfaction with prostate cancer care.[65] Furthermore, the literature indicated that manual workers were less likely to receive a bone scan and radical prostatectomy, and they had higher overall and cancer-specific mortalities as compared to non-manual employees.[66]

3. Healthcare encounter factors

The factors under which the healthcare encounters took place influenced the HCP-user relationship and experiences of users. The literature discussed, among other issues, HCP-user communication and the power and autonomy of men.

3.1. Information and communication

Information and communication were recurring themes in all SRH subject areas. More than half of the studies touched upon some aspect of information, or the way it is delivered and communicated. Receiving information was described as valuable and important and made men feel pleased, satisfied and empowered.[52,67-72] During the birth process, for example, information helped men to feel included and to find their place in supporting their partners and facilitated the decision-making of couples.[32,42,45,67] Contrarily, lack of sufficient information was associated with more concerns and feelings of exclusion and dissatisfaction.[40,73] Insufficient information was reported in various healthcare settings, for example, the effects of antenatal care,[36,74-76] infertility care[69] and cancer treatment/surgery on sexual health.[63,77]

The literature also discussed the format of information. Oral information was especially preferred when the matter aroused many questions, such as communicating an infertility diagnosis[35] or HPV vaccination,[47] while written information was considered more suitable in other cases, such as HIV and STI information for MSM.[70] However, even though recommended by the National Board of Health and Welfare in Sweden, studies have shown that the majority of men did not receive written information about prostate cancer screening and some were not even aware that they underwent the screening.[64] In other cases, a combination of oral and written information was considered easier to comprehend, for example, when communicating the side effects of cancer treatment on fertility[34] (see Box 2).

For peer review only - http://bmjopen.bmj.com/site/about/guidelines.xhtml

Box 2: **The charactersitics of satisfying information and communication - men's views**

- **Clear and simple language**: Clear and proper level information were perceived as important. The inability to understand the medical language of HCPs caused distress.[32,34,44,67]
- **Reliable**: Contradictions, exaggerations and lack of reliable information caused frustration.[33,42] Exaggerated information was associated with unease, confusion and a sense of not being taken seriously.[52] Men wanted to feel welcome when asking questions and wanted honest, consistent and clear answers.[41,42] Men expressed a need for help to choose reliable websites and organize and discuss the information received.[14]
- **Personalized and relevant**: While general information could be obtained from the Internet, receiving personalized and relevant information from the HCPs was a high priority[26,46,67] For example, an online patient-nurse communication service played a central role in providing personalized information for cancer patients.[78]
- **Comprehensive and sufficient**: Receiving adequate and comprehensive information was regarded as important.[52] For example, men highlighted the need for a deeper dialogue about personal experiences or the psychological concequences of male infertility,[26] as well as psychological support during waiting times for cancer treatment.[77]
- **Appropriate and interactive**: The way HCPs communicated the information affected men's feelings; a possitive attitude and "a good mood" among HCPs mirrored less stress in men.[73] Having time to ask questions and interact with HCPs was also appreciated.[31]
- **Timely**: Constant updates of information during their partner's labour and birth was highly appreciated by men. Men who received timely information felt well informed, calm, secured and satisfied.[32,41,60,73] On the other hand, receiving information at inappropriate times was perceived as insufficient.[77]
- **Inclusive**: Involving men in the communication as an equal partner in reproductive healthcare was perceived as necessary.[26]

3.2. Lack of control and compromised autonomy in reproductive healthcare

Men's engagement in reproductive healthcare seemed to be a complex matter; midwives valued men's involvement, to a certain point, since they experienced over involvement as a possible sign of controlling behaviour or intimate partner violence.[17] The literature discussed men's involvement and their lack of control and compromised autonomy in various situations in reproductive healthcare, especially during pregnancy and birth. For example, the inability to help or act during their partner's birth made men experience lack of power and control.[45,73,79] Similarly, the uncontrollable process of non-progressing delivery left men with a feeling of helplessness and insecurity.[45] Men appreciated being involved in the decision regarding their partner's elective or emergency caesarean section, but 40% of the men felt they were not involved enough.[76,80] Also, men reported being more in control and more involved in decision-making during an elective caesarean section or normal spontaneous vaginal birth as compared to emergency caesarean section or assisted vaginal birth.[73,76,80] However, they also described situations where they were forced to participate in tasks and rituals without their consent, (i.e., cutting the umbilical cord or touching the child's head before the baby was born).[44] Even though involvement in decision-making during birth was associated with higher

For peer review only - http://bmjopen.bmj.com/site/about/guidelines.xhtml

satisfaction,[40,76] it was still important to be able to choose whether to participate or not in different stages of birth.[41]

Compromised autonomy was also reported in the infertility clinic[81] and when banking sperm before cancer treatment.[31] To the contrary, control and involvement in decisions were more satisfactory during home abortions. The pregnant woman made the decision, but the partner's opinion was important for her.[52,71]

3.3. Good treatment increases security and satisfaction

Men wanted HCPs to treat them as persons, respecting their needs, feelings and experiences. HCPs should try to understand the unique situation of each man and take it seriously.[35,41,46,52] Respectful treatment was highly expected and associated with higher satisfaction with care.[61,82] It was especially important to deliver negative news with sensitivity.[42,67] Men who experienced HCPs as professional, empathetic and attentive, felt satisfied, important and "not just a number".[5,35,50,52] In other cases, men perceived insensitivity and lack of respect or attention in the comments of HCPs, resulting in feeling disappointed and dissatisfied.[40,52,67]

The support of midwives during antenatal, intrapartum and postnatal care was necessary and created a feeling of security and satisfaction. Providing attention and information and addressing men's needs and questions helped men to build trust in the midwives and be supportive to their partners.[41,46,60,73-76] However, men were not always satisfied with the support of midwives, which made men feel insecure, helpless and worried.[40,76,79]

3.4. Confidentiality, a prerequisite to access to SRHC

Confidentiality was considered an essential condition to access certain SRH services, including youth clinics and HIV testing. For example, fear of being recognized in the clinic was one of the main reasons for not being tested for HIV.[49] Getting an HIV test was considered as implicitly disclosing same-sex sexuality, which led to preferring self-testing as an anonymous alternative, especially among non-gay MSM and those who had never been tested for HIV.[54] Therefore, anonymous HIV testing outside the healthcare system were requested and considered helpful for MSM.[51] Similarly, young people visiting youth clinics expressed the importance of HCPs' confidentiality and that they are used to and only work with young people.[5] Trust in HCPs' confidentiality was also described as important in the process of men disclosing victimization.[28]

4. Healthcare system factors

The healthcare system influenced the HCP-user relationship through its effects on HCPs and the healthcare encounters. Among other issues, the literature discussed the organization of healthcare, the holistic approach (or the lack of it), SRHC as traditionally women-centred care and men's SRHC as "nobody's mandate".

4.1. Men's SRH is not a priority

The literature indicated that the clinical training and organization of care does not give men's SRH enough priority. Nurses, for example, highlighted the lack of basic medical training and organizational support to deal with men's sexual health issues. Their main source of knowledge about men's SRH was received from pharmaceutical companies.[16] Similarly, midwifery education and clinical training doesn't regularly include andrology, which together with lack of time and organizational support hindered them from providing counselling to men.[17]

For peer review only - http://bmjopen.bmj.com/site/about/guidelines.xhtml

Another example of the low priority of men's SRH was the lack of follow-up and continuity of care, which was reported in various services. For example, men reported not being followed-up after being prescribed medication for sexual function.[27] Additionally, the stays of men with the family after delivery was not welcomed in some hospitals, even though this was important for men in order to feel supported and to support their new family.[46]

The lack of prioritizing men's SRH was also reflected by the few prevention activities that healthcare performs regarding men's SRH. For example, the vast majority of MSM did not encounter any HIV/STI prevention services, despite the importance of making it more available.[70] Another example was the missed opportunity to counsel for sexual health in around one-third of men testing for HIV.[50]

4.2. Lack of holistic care

The literature discussed the lack of holistic care in SRH services. For example, psychological aspects of infertility were usually not acknowledged and therefore overlooked.[35] For couples with repeated pregnancy loss, psychological counselling was restricted to a few with certain criterion and also without considering individual situations.[42] Furthermore, antenatal care was perceived to focus mainly on medical support and rarely on emotional and psychological support, leaving only few users being very satisfied with this aspect of antenatal care.[14,74] Consequently, men who were subjected to gender-based violence were less likely to seek help unless they had severe physical injuries.[28]

4.3. Women-centred reproductive healthcare, a compromised right for inclusion of men

Both men and women expressed a wish to include men and to focus on "the couple" rather than one partner, that is, equal partners sharing a common reason for visiting reproductive healthcare.[15,35,42,52] Even though men felt that the focus on women in reproductive healthcare is reasonable, they stated that this attention should not exclude men.[35,48] The feeling of exclusion was experienced by men in different reproductive health services, including fertility and antenatal care.[14,15,37,48] One study showed that the investigations and treatments focused only on the women, even when the cause of infertility was a low sperm count, which led to perceive infertility care as the "women's world".[15] Additionally, the midwives discussed sexual and reproductive rights for men as being women's partners rather than being men's own rights, and men's concerns about contraception are dependent upon his partner's choice to include him or not in contraceptive counselling.[17]

4.4. Men's SRH is nobody's responsibility

Different HCPs expressed their concern about men's sexual health as "no one's responsibility". The attitudes of midwives toward providing counselling to men were divided. Some were positive and found it a continuation of their current responsibilities that concerned women.[17] This opinion was shared by men of pregnant partners who expressed their trust and faith in midwives and saw them as the best ones to promote sexual health among men.[48] Other midwives were reluctant and expressed their difficulty in providing counselling to men. For them, the pregnancy is about the woman's body, and thus, man's participation was not evident.[17] Nurses also questioned if men's sexual health is their duty, especially if it included an emotional aspect. In their opinion, primary care was not equipped to deal with sexual health problems; therefore, they often referred patients to other healthcare units.[16]

For peer review only - http://bmjopen.bmj.com/site/about/guidelines.xhtml

5. Sociopolitical factors

The outer layer of the model (Figure 4) discusses the social and political factors, including social and gender norms, which affect the healthcare system and the attitudes and behaviours of HCPs and users.

5.1. Social and gender norms

The literature described traditional social and gender norms as contributing to setting values for men that hinder their abilities to cope or seek help and affect their sexual health and wellbeing. For example, infertility was described as a "malfunction of manhood" that is faced by denial and changes how men perceive their masculinities. The absence of sperm was an identity question and a threat to men's masculinities.[15,26,31,67] Similarly, suffering the decline in sexual function associated with a diagnosis of prostate cancer was perceived as threatening to the male identity and therefore accompanied by feelings of inadequacy and not being a "real man".[27,77]

Furthermore, the attempts of men to conform to traditional masculinity norms affected their ability to talk about experiences of violence, especially if they were exposed to intimate partner violence.[28] Additionally, transmen had experiences of vulnerability during gynaecological examination or when they resumed menstrual bleeding after family planning treatment. This was perceived as stressful, humiliating and uncomfortable, as well as a reminder of a sex they "wanted to forget".[83]

These "threatened masculinities" were also reflected through men's health seeking behaviours. Men disregarded their sexual health, delayed admission of the problem and opted to distance themselves from seeking healthcare.[13] For example, young men had more difficulties to admit their SRH needs and to seek help as compared to young women.[5] Similarly, the midwives indicated that men only seek help when they have severe symptoms, while also noting that young men are increasingly attending STI testing and are more open to discuss sexual health.[17]

Men expressed increased social expectations on them to be more involved in healthcare during pregnancy and birth, which corresponded to personal willingness and desire to share responsibility for the security and support of their partners.[27,38,44,46] Men were also eager to participate in other reproductive healthcare services, such as infertility treatment and home abortion.[35,71,82] However, men were faced with barriers in their desire to participate and experienced "paddling upstream" to fulfil their involvement.[39]

The literature also discussed how social and gender norms affect the healthcare system being perceived as women-centred. Youth clinics, for example, were perceived as a place for the SRH of girls, which created a barrier for young men seeking healthcare.[5] Additionally, the social norms hindered HCPs talking about sexual health, especially when the patient is older than the HCP.[16] In turn, HCPs reinforced these social norms by supporting the traditional gender expectations of the woman as the primary infant caregiver and overlooking the importance of shared parenthood and including the man in infant care.[38,84]

Studies also described how social and gender norms affected the way healthcare deals with victimized men. The training and education the emergency departments offered in Sweden about caring for violence victims focuses only on women and children and not victimized men.[85] Similar experiences of the reinforcement of traditional gender positions by HCPs were perceived by men subjected to intimate partner violence. These men felt alone since society did not acknowledge their experiences, and the HCPs expected them to embody traditional ideals of masculinities.[28]

For peer review only - http://bmjopen.bmj.com/site/about/guidelines.xhtml

5.2. Policies

Politics and policies were rarely discussed in the literature, but there were some mentions of the regulations and guidelines in SRHC, which have been discussed under point 4. The only mention of policy was in the context of gender-based violence. While most of the counties and emergency departments in Sweden had a policy about the care for victims of violence, these policies focus merely on women and children but not men or other groups.[85]

Discussion

In the previous section we reviewed, charted and synthesized the available literature in relation to the factors influencing men's experiences in SRHC. To summarize, the majority of the reviewed literature discussed men's experiences in reproductive healthcare, mainly care related to infertility, pregnancy and birth. The literature lacked men's perspectives on contraception, including condom use and vasectomy. Regarding sexual healthcare, the available literature captured mainly STIs and HIV treatment and prevention but not men's experiences in other sexual health issues, such as impotence or gender-based violence. The literature also lacked the perspectives of particular groups of men, such as Indigenous, national minorities and men with functional variations. Furthermore, migrants and MSM were almost only mentioned in relation to HIV treatment and prevention.

The literature indicated that men face difficulties to be included in reproductive healthcare, where they are mostly treated as an accompanying partner, receiving little attention. The knowledge and attitudes of HCPs were crucial for their ability to discuss men's SRH and also for men's experiences in SRHC. Furthermore, the literature rarely discussed healthcare organization and policies and how they affect men's health seeking behaviours and experiences in SRHC. Lastly, men's right to SRH is usually not stressed in the literature, unless it is related to a specific group of men, such as MSM and transmen.

While we presented the factors influencing men's experiences in SRHC in separate levels and the reviewed articles did not explicitly study the interaction between these levels, the theoretical framework still enables us to understand the interaction between these determinants. We presented some examples in the results of how these levels are linked and influence each other. The interaction between gender and social norms with the other determinants might be of special significance. For example, the literature described how traditional social and gender norms affect the attitudes and behaviours of HCPs. A clear example of this was how men were treated as an accompanying partner during healthcare visits related to infertility, antenatal care and birth. In many cases men are still not seen as an equal partner or as a primary caregiver for their new-borns, which likely influences the attitudes of HCPs toward men seeking antenatal care, in turn affecting men's experiences of those services negatively.[14,39]

While traditional gender norms and values of masculinities provide important pieces in explaining men's health seeking behaviours, a more comprehensive picture of men's experiences in SRHC is needed. The literature showed other determinants of men's experiences in SRHC, including how the healthcare system is organized. It seems SRHC in the Nordic countries focuses mainly on women, while there is a lack of knowledge about men's SRH and no clear entry for men into SRHC. The healthcare system should adapt a gender-responsive approach that ensures accessible healthcare services for men and which through its approach addresses the impacts of gender norms on men, women and HCPs.[2,86]

For peer review only - http://bmjopen.bmj.com/site/about/guidelines.xhtml

To reach universal access to SRHC and gender equity, it is of importance to engage men in SRH and ensure that their needs are met.[87] Improving men's experiences in SRHC in the Nordic countries is not only important for improving men's SRH but also could enable men to strengthen their support of women's SRH and thus gender equality.[87,88] Meeting men's needs for SRHC could consequently decrease STIs, unintended pregnancies and improve parenting and family relationships.[9,21] Such specific focus on men in the SRHC organization to improve men's health and rights with the goal to contribute to gender equality will benefit both men and women.[89,90]

Strengths and limitations of this study

To the best of our knowledge, this review is the first to examine the experiences of men in SRHC in the Nordic countries. The review provides interesting and important information about these experiences, by organizing them in a theoretical framework that make it easier to understand and draw conclusions.

Even though we developed and followed our search strategies thoroughly, the review has some limitations. The broad nature of the field and the wide variety of terms related to SRH make it difficult to assure the inclusion of all relevant literature. Additionally, due to practical reasons, the search was restricted to two databases but complemented with manually screening the reference lists of the identified literature. Another strength of this review was the use of a Nordic-specific database without restriction to language, which ensured an equal inclusion of the literature from other Nordic countries, even though most of the literature in this review was published in Sweden.

Furthermore, the adapted framework allowed us to use a relevant ecological lens on men's experiences in SRHC and to systematically identify and categorize the concepts discussed in the selected literature. However, the use of this framework might have caused us to overlook aspects of the research topic that fell outside the interest of this scoping review.

Finally, it is important to note that, when reporting results and discussing them, we choose to implicitly treat the Nordic countries as essentially similar. While we argue that this makes sense because of the actual similarities between these countries and their healthcare systems, we also acknowledge that this might obscure important differences between or within countries (e.g., relation to place of residence (rural vs. urban) or cultural differences).

Conclusion

Despite the uncontroversial importance of men's right to access SRHC on equal terms, the available literature indicated that SRH is mainly the domain of women and healthcare around men's SRH is not sufficiently prioritized. A more comprehensive picture of men's experiences in SRHC is needed.

There is a lack of knowledge about men's SRH and no clear entry for men into SRHC. This indicates the necessity for improvements in the medical education of HCPs and in health system interventions. Further research should examine the influence of policies and the healthcare organization on men's access and experiences in SRHC and explore the identified knowledge gaps of men's experiences in SRHC related to sexual function, contraceptive use and gender-based violence.

For peer review only - http://bmjopen.bmj.com/site/about/guidelines.xhtml

Author contributions

MB was the lead reviewer and author of the manuscript. AKH and JPS contributed to the identification and selection of articles. All authors contributed to data interpretation. All authors provided comments and agreed on the final version of the manuscript. AKH was the project leader.

Declaration of interests

The authors declare that they have no conflict of interest.

Funding

This study was supported by the Public Health Agency of Sweden (Folkhälsomyndigheten). The Public Health Agency of Sweden was not involved in the analysis, interpretation, writing or the decision to submit this paper for publication.

Word count

5365 words

For peer review only

References

1. Osika Friberg I, Krantz G, Määttä S, Järbrink K. Sex differences in health care consumption in Sweden: a register-based cross-sectional study. *Scandinavian journal of public health*. 2016;**44**(3):264–73.
2. Europe WHO. Strategy on the health and well-being of men in the WHO European Region. Regional Committee for Europe; 2018.
3. Nicolosi A, Buvat J, Glasser DB, Hartmann U, Laumann EO, Gingell C. Sexual behaviour, sexual dysfunctions and related help seeking patterns in middle-aged and elderly Europeans: the global study of sexual attitudes and behaviors. *World journal of urology*. 2006;**24**(4):423–28.
4. Folkhälsomyndigheten. Sexuell och reproduktiv hälsa och rättigheter i Sverige 2017 Stockholm2019. Available from: http://www.folkhalsomyndigheten.se.
5. Sollesnes R. Exploring issues that motivate contact with adolescent health. *Vård i Norden*. 2010;**30**(2):4–7.
6. FSUM [Föreningen för Sveriges ungdomsmottagningar]. Handbok för Sveriges ungdomsmotagningar. [Handbook of Sweden's youth clinics] 2018. Available from: http://www.fsum.nu/wp-content/uploads/2018/05/handbok_original_utskrift.pdf.
7. Danielsson M, Berglund T, Forsberg M, Larsson M, Rogala C, Tydén T. Sexual and reproductive health: Health in Sweden: The National Public Health Report 2012. Chapter 9. *Scandinavian journal of public health*. 2012;**40**(9_suppl):176–96.
8. Kato-Wallace J, Barker G, Sharafi L, Mora L, Lauro G. Adolescent boys and young men: engaging them as supporters of gender equality and health and understanding their vulnerabilities. *Washington, DC and New York: Promundo and UNFPA*. 2016.
9. World Health Organization. The health and well-being of men in the WHO European Region: better health through a gender approach: World Health Organization. Regional Office for Europe; 2018.
10. Brannstrom J, Svedhem Johansson V, Marrone G, Wendahl S, Yilmaz A, Blaxhult A, et al. Deficiencies in the health care system contribute to a high rate of late HIV diagnosis in Sweden. *HIV medicine*. 2016;**17**(6):425–35.
11. Gios L, Mirandola M, Sherriff N, Toskin I, Blondeel K, Dias S, et al. Being in the closet. correlates of Outness among MSM in 13 European cities. *Journal of homosexuality*. 2021;**68**(3):415–33.
12. Hearn J, Nordberg M, Andersson K, Balkmar D, Gottzén L, Klinth R, et al. Hegemonic masculinity and beyond: 40 years of research in Sweden. *Men and masculinities*. 2012;**15**(1):31–55.
13. Christianson M, Boman J, Essen B. "Men don't think that far" - Interviewing men in Sweden about chlamydia and HIV testing during pregnancy from a discursive masculinities construction perspective. *Sexual & reproductive healthcare : official journal of the Swedish Association of Midwives*. 2017;**12**:107–15.
14. Widarsson M, Kerstis B, Sundquist K, Engström G, Sarkadi A. Support needs of expectant mothers and fathers: a qualitative study. *The Journal of perinatal education*. 2012;**21**(1):36–44.
15. Johansson M, Hellstrom AL, Berg M. Severe male infertility after failed ICSI treatment--a phenomenological study of men's experiences. *Reproductive health*. 2011;**8**:4.
16. Klaeson K, Hovlin L, Guva H, Kjellsdotter A. Sexual health in primary health care - a qualitative study of nurses' experiences. *Journal of clinical nursing*. 2017;**26**(11-12):1545–54.
17. Grandahl M, Bodin M, Stern J. In everybody's interest but no one's assigned responsibility: midwives' thoughts and experiences of preventive work for men's sexual and reproductive health and rights within primary care. *BMC public health*. 2019;**19**(1):1423.
18. European institute for gender equality. Gender Equality Index 2020 [Available from: https://eige.europa.eu/gender-equality-index/2020/compare-countries.

For peer review only - http://bmjopen.bmj.com/site/about/guidelines.xhtml

19. Levy JK, Darmstadt GL, Ashby C, Quandt M, Halsey E, Nagar A, et al. Characteristics of successful programmes targeting gender inequality and restrictive gender norms for the health and wellbeing of children, adolescents, and young adults: a systematic review. *The Lancet Global Health*. 2020;**8**(2):e225–e36.
20. Gupta GR, Oomman N, Grown C, Conn K, Hawkes S, Shawar YR, et al. Gender equality and gender norms: framing the opportunities for health. *The Lancet*. 2019;**393**(10190):2550–62.
21. WHO Europe. Strategy on the health and well-being of men in the WHO European Region. Regional Committee for Europe; 2018.
22. White A, De Sousa B, De Visser R, Hogston R, Madsen S, Matara P, et al. The State of Men's Health in Europe: extended report. *Brussels: European Commission Directorate-General for Health and Consumers*. 2011.
23. Folkhälsomyndigheten. Motiverande samtal med unga om sexuell hälsa. En kartläggande litteraturöversikt inom omraadet hivprevention samt sexuell och reproduktiv hälsa och rättigheter (SRHR). 2018.
24. Organization WH. Sexual health and its linkages to reproductive health: an operational approach. 2017.
25. Kilbourne AM, Switzer G, Hyman K, Crowley-Matoka M, Fine MJ. Advancing health disparities research within the health care system: a conceptual framework. *American journal of public health*. 2006;**96**(12):2113–21.
26. Mikkelsen AT, Madsen SA, Humaidan P. Psychological aspects of male fertility treatment. *Journal of advanced nursing*. 2013;**69**(9):1977–86.
27. Klaeson K, Sandell K, Bertero CM. Talking about sexuality: desire, virility, and intimacy in the context of prostate cancer associations. *American journal of men's health*. 2013;**7**(1):42–53.
28. Simmons J, Bruggemann AJ, Swahnberg K. Disclosing victimisation to healthcare professionals in Sweden: a constructivist grounded theory study of experiences among men exposed to interpersonal violence. *BMJ open*. 2016;**6**(6):e010847.
29. Vik A, Brekke M. Do patients consult their GP for sexual concerns? A cross sectional explorative study. *Scand J Prim Health Care*. 2017;**35**(4):373–78.
30. Micaux Obol C, Armuand GM, Rodriguez-Wallberg KA, Ahlgren J, Ljungman P, Wettergren L, et al. Oncologists and hematologists' perceptions of fertility-related communication - a nationwide survey. *Acta oncologica (Stockholm, Sweden)*. 2017;**56**(8):1103–10.
31. Armuand GM, Wettergren L, Rodriguez-Wallberg KA, Lampic C. Women more vulnerable than men when facing risk for treatment-induced infertility: a qualitative study of young adults newly diagnosed with cancer. *Acta oncologica (Stockholm, Sweden)*. 2015;**54**(2):243–52.
32. Johansson M, Thies-Lagergren L. Swedish fathers' experiences of childbirth in relation to maternal birth position: a mixed method study. *Women and birth : journal of the Australian College of Midwives*. 2015;**28**(4):e140–7.
33. Herder T, Agardh A. Navigating between rules and reality: a qualitative study of HIV positive MSM's experiences of communication at HIV clinics in Sweden about the rules of conduct and infectiousness. *AIDS care*. 2019;**31**(10):1304–10.
34. Oster I, Hedestig O, Johansson M, Klingstedt N, Lindh J. Sharing experiences in a support group: men's talk during the radiotherapy period for prostate cancer. *Palliative & supportive care*. 2013;**11**(4):331–9.
35. Sylvest R, Furbringer JK, Schmidt L, Pinborg A. Infertile men's needs and assessment of fertility care. *Upsala journal of medical sciences*. 2016;**121**(4):276–82.
36. Andersson E, Christensson K, Hildingsson I. Parents' experiences and perceptions of group-based antenatal care in four clinics in Sweden. *Midwifery*. 2012;**28**(4):502–8.
37. Asenhed L, Kilstam J, Alehagen S, Baggens C. Becoming a father is an emotional roller coaster - an analysis of first-time fathers' blogs. *Journal of clinical nursing*. 2014;**23**(9-10):1309–17.

For peer review only - http://bmjopen.bmj.com/site/about/guidelines.xhtml

38. Johnsen H, Stenback P, Hallden BM, Crang Svalenius E, Persson EK. Nordic fathers' willingness to participate during pregnancy. *Journal of reproductive and infant psychology*. 2017;**35**(3):223–35.

39. Widarsson M, Engström G, Tydén T, Lundberg P, Hammar LM. 'Paddling upstream': Fathers' involvement during pregnancy as described by expectant fathers and mothers. *Journal of clinical nursing*. 2015;**24**(7-8):1059–68.

40. Johansson M, Rubertsson C, Rådestad I, Hildingsson I. Childbirth–an emotionally demanding experience for fathers. *Sexual & Reproductive Healthcare*. 2012;**3**(1):11–20.

41. Bäckström C, Wahn EH. Support during labour: first-time fathers' descriptions of requested and received support during the birth of their child. *Midwifery*. 2011;**27**(1):67–73.

42. Koert E, Malling GMH, Sylvest R, Krog MC, Kolte AM, Schmidt L, et al. Recurrent pregnancy loss: couples' perspectives on their need for treatment, support and follow up. *Human reproduction (Oxford, England)*. 2019;**34**(2):291–96.

43. Fabian H, Sarkadi A, Åhman A. Challenges and benefits of conducting parental classes in Sweden: Midwives' perspectives. *Sexual & Reproductive Healthcare*. 2015;**6**(4):236–42.

44. Premberg Å, Carlsson G, Hellström A-L, Berg M. First-time fathers' experiences of childbirth—A phenomenological study. *Midwifery*. 2011;**27**(6):848–53.

45. Hasman K, Kjaergaard H, Esbensen BA. Fathers' experience of childbirth when non-progressive labour occurs and augmentation is established. A qualitative study. *Sexual & Reproductive Healthcare*. 2014;**5**(2):69–73.

46. Persson EK, Fridlund B, Kvist LJ, Dykes A-K. Fathers' sense of security during the first postnatal week—A qualitative interview study in Sweden. *Midwifery*. 2012;**28**(5):e697–e704.

47. Grandahl M, Nevéus T, Dalianis T, Larsson M, Tydén T, Stenhammar C. 'I also want to be vaccinated!'–adolescent boys' awareness and thoughts, perceived benefits, information sources, and intention to be vaccinated against Human papillomavirus (HPV). *Human vaccines & immunotherapeutics*. 2019;**15**(7-8):1794–802.

48. Christianson M, Boman J, Essen B. 'Let men into the pregnancy'--men's perceptions about being tested for chlamydia and HIV during pregnancy. *Midwifery*. 2013;**29**(4):351–8.

49. Persson KI, Berglund T, Bergstrom J, Eriksson LE, Tikkanen R, Thorson A, et al. Motivators and barriers for HIV testing among men who have sex with men in Sweden. *Journal of clinical nursing*. 2016;**25**(23-24):3605–18.

50. Berg RC. Predictors of never testing for HIV among a national online sample of men who have sex with men in Norway. *Scandinavian Journal of Public Health*. 2013;**41**(4):398–404.

51. Stromdahl S, Liljeros F, Thorson AE, Persson KI, Forsberg BC. HIV testing and prevention among foreign-born Men Who have Sex with Men: an online survey from Sweden. *BMC public health*. 2017;**17**(1):139.

52. Makenzius M, Tyden T, Darj E, Larsson M. Autonomy and dependence - experiences of home abortion, contraception and prevention. *Scandinavian Journal of Caring Sciences*. 2013;**27**(3):569–79.

53. Marrone G, Mellgren A, Eriksson LE, Svedhem V. High Concordance between Self-Reported Adherence, Treatment Outcome and Satisfaction with Care Using a Nine-Item Health Questionnaire in InfCareHIV. *PloS one*. 2016;**11**(6):e0156916.

54. Bjornshagen V, Moseng BU, Ugreninov E. Who Do You Reach? A Norwegian Pilot Project on HIV Self-Testing that Targeted Men Who Have Sex with Men. *AIDS and behavior*. 2020;**24**(2):568–79.

55. Schmidt AJ, Hickson F, Weatherburn P, Marcus U. Comparison of the performance of STI screening services for gay and bisexual men across 40 European cities: results from the European MSM Internet Survey. *Sexually transmitted infections*. 2013;**89**(7):575–82.

56. Hoyos J, Mate T, Indave BI, Agusti C, Chanos S, Pichon F, et al. Knowledge, actual and potential use of HIV self-sampling testing kits among MSM recruited in eight European countries. *HIV medicine*. 2018;**19 Suppl 1**:27–33.

For peer review only - http://bmjopen.bmj.com/site/about/guidelines.xhtml

57. Stromdahl S, Hoijer J, Eriksen J. Uptake of peer-led venue-based HIV testing sites in Sweden aimed at men who have sex with men (MSM) and trans persons: a cross-sectional survey. *Sexually transmitted infections*. 2019;**95**(8):575–79.
58. Hildingsson I, Sjöling M. Fathers' experiences of support during pregnancy and the first year following childbirth–findings from a Swedish regional survey. *Journal of Men's Health*. 2011;**8**(4):258–66.
59. Schytt E, Bergstrom M. First-time fathers' expectations and experiences of childbirth in relation to age. *Midwifery*. 2014;**30**(1):82–8.
60. Hildingsson I, Cederlöf L, Widén S. Fathers' birth experience in relation to midwifery care. *Women and birth*. 2011;**24**(3):129–36.
61. Andersson E, Norman Å, Kanlinder C, Plantin L. What do expectant fathers expect of antenatal care in Sweden? A cross-sectional study. *Sexual & Reproductive Healthcare*. 2016;**9**:27–34.
62. Armuand GM, Rodriguez-Wallberg KA, Wettergren L, Ahlgren J, Enblad G, Hoglund M, et al. Sex differences in fertility-related information received by young adult cancer survivors. *Journal of clinical oncology : official journal of the American Society of Clinical Oncology*. 2012;**30**(17):2147–53.
63. Rasmusson EM, Plantin L, Elmerstig E. 'Did they think I would understand all that on my own?' A questionnaire study about sexuality with Swedish cancer patients. *European journal of cancer care*. 2013;**22**(3):361–9.
64. Fridriksson J, Gunseus K, Stattin P. Information on pros and cons of prostate-specific antigen testing to men prior to blood draw: a study from the National Prostate Cancer Register (NPCR) of Sweden. *Scandinavian journal of urology and nephrology*. 2012;**46**(5):326–31.
65. Bergengren O, Garmo H, Bratt O, Holmberg L, Johansson E, Bill-Axelson A. Satisfaction with Care Among Men with Localised Prostate Cancer: A Nationwide Population-based Study. *European urology oncology*. 2018;**1**(1):37–45.
66. Berglund A, Garmo H, Robinson D, Tishelman C, Holmberg L, Bratt O, et al. Differences according to socioeconomic status in the management and mortality in men with high risk prostate cancer. *European journal of cancer (Oxford, England : 1990)*. 2012;**48**(1):75–84.
67. Sylvest R, Koert E, Vittrup I, Birch Petersen K, Hvidman HW, Hald F, et al. Men's expectations and experiences of fertility awareness assessment and counseling. *Acta obstetricia et gynecologica Scandinavica*. 2018;**97**(12):1471–77.
68. Bodin M, Tyden T, Kall L, Larsson M. Can Reproductive Life Plan-based counselling increase men's fertility awareness? *Upsala journal of medical sciences*. 2018;**123**(4):255–63.
69. Holter H, Sandin-Bojo AK, Gejervall AL, Wikland M, Wilde-Larsson B, Bergh C. Patient-centred quality of care in an IVF programme evaluated by men and women. *Human reproduction (Oxford, England)*. 2014;**29**(12):2695–703.
70. Qvarnstrom A, Oscarsson MG. Experiences of and attitudes towards HIV/STI prevention among holidaymaking men who have sex with men living in Sweden: a cross-sectional Internet survey. *Scand J Public Health*. 2015;**43**(5):490–6.
71. Kero A, Lalos A, Wulff M. Home abortion–experiences of male involvement. *The European Journal of Contraception & Reproductive Health Care*. 2010;**15**(4):264–70.
72. Åhman A, Lindgren P, Sarkadi A. Facts first, then reaction—Expectant fathers' experiences of an ultrasound screening identifying soft markers. *Midwifery*. 2012;**28**(5):e667–e75.
73. Johansson M, Hildingsson I, Fenwick J. Important factors working to mediate Swedish fathers' experiences of a caesarean section. *Midwifery*. 2013;**29**(9):1041–49.
74. Jungmarker EB, Lindgren H, Hildingsson I. Playing second fiddle is okay–Swedish fathers' experiences of prenatal care. *The Journal of Midwifery & Women's Health*. 2010;**55**(5):421–29.
75. Andersson E, Small R. Fathers' satisfaction with two different models of antenatal care in Sweden - Findings from a quasi-experimental study. *Midwifery*. 2017;**50**:201–07.

For peer review only - http://bmjopen.bmj.com/site/about/guidelines.xhtml

76. Johansson M, Hildingsson I. Intrapartum care could be improved according to Swedish fathers: mode of birth matters for satisfaction. *Women and Birth*. 2013;**26**(3):195–201.
77. Schildmeijer K, Frykholm O, Kneck A, Ekstedt M. Not a Straight Line-Patients' Experiences of Prostate Cancer and Their Journey Through the Healthcare System. *Cancer nursing*. 2019;**42**(1):E36–e43.
78. Wibe T, Hellesø R, Varsi C, Ruland C, Ekstedt M. How does an online patient-nurse communication service meet the information needs of men with recently diagnosed testicular cancer? *International Scholarly Research Notices*. 2012;**2012**.
79. Thies-Lagergren L, Johansson M. Intrapartum midwifery care impact Swedish couple's birth experiences - A cross-sectional study. *Women and birth : journal of the Australian College of Midwives*. 2019;**32**(3):213–20.
80. Johansson M, Hildingsson I, Fenwick J. 'As long as they are safe–Birth mode does not matter'Swedish fathers' experiences of decision-making around caesarean section. *Women and Birth*. 2014;**27**(3):208–13.
81. Holter H, Gejervall AL, Borg K, Sandin-Bojo AK, Wikland M, Wilde-Larsson B, et al. In vitro fertilization healthcare professionals generally underestimate patients' satisfaction with quality of care. *Acta obstetricia et gynecologica Scandinavica*. 2017;**96**(3):302–12.
82. Makenzius M, Tyden T, Darj E, Larsson M. Women and men's satisfaction with care related to induced abortion. *The European journal of contraception & reproductive health care : the official journal of the European Society of Contraception*. 2012;**17**(4):260–9.
83. Armuand G, Dhejne C, Olofsson JI, Rodriguez-Wallberg KA. Transgender men's experiences of fertility preservation: a qualitative study. *Human reproduction (Oxford, England)*. 2017;**32**(2):383–90.
84. Erlandsson K, Häggström-Nordin E. Prenatal parental education from the perspective of fathers with experience as primary caregiver immediately following birth: a phenomenographic study. *The journal of perinatal education*. 2010;**19**(1):19.
85. Linnarsson JR, Benzein E, Årestedt K, Erlingsson C. Preparedness to care for victims of violence and their families in emergency departments. *Emergency medicine journal*. 2013;**30**(3):198–201.
86. Kågesten A, Chandra-Mouli V. Gender-transformative programmes: implications for research and action. *The Lancet Global Health*. 2020;**8**(2):e159–e60.
87. Starrs AM, Ezeh AC, Barker G, Basu A, Bertrand JT, Blum R, et al. Accelerate progress—sexual and reproductive health and rights for all: report of the Guttmacher–Lancet Commission. *The Lancet*. 2018;**391**(10140):2642-92.
88. Dahl S, Sonnerby C, Järvklo N. Män och jämställdhet; Betänkande av Utredningen om män och jämställdhet. Utredningar SO; 2014. Report No.: SOU 2014:6.
89. Ruane-McAteer E, Gillespie K, Amin A, Aventin Á, Robinson M, Hanratty J, et al. Gender-transformative programming with men and boys to improve sexual and reproductive health and rights: a systematic review of intervention studies. *BMJ global health*. 2020;**5**(10):e002997.
90. Bustamante-Forest R, Giarratano G. Changing men's involvement in reproductive health and family planning. *The Nursing Clinics of North America*. 2004;**39**(2):301–18.
91. Linnarsson JR, Benzein E, Arestedt K, Erlingsson C. Preparedness to care for victims of violence and their families in emergency departments. *Emergency medicine journal : EMJ*. 2013;**30**(3):198–201.
92. Qvist T, Cowan SA, Graugaard C, Helleberg M. High linkage to care in a community-based rapid HIV testing and counseling project among men who have sex with men in Copenhagen. *Sexually transmitted diseases*. 2014;**41**(3):209–14.

For peer review only - http://bmjopen.bmj.com/site/about/guidelines.xhtml

For peer review only

For peer review only - http://bmjopen.bmj.com/site/about/guidelines.xhtml

Appendices

Appendix 1: Söktermer

1) Pubmed/Medline

Search	Search terms	Nr. Of articles
**#1**<br>**Sexual and reproductive health**	(Sexual Health[mesh] OR "Sexual health" OR Reproductive Health[mesh] OR "Reproductive health" OR<br>"Sexual and reproductive health" OR "Sexual and reproductive health and rights" OR SRHR OR<br><br>"sexual function" OR "sexual functions" OR "sexual dysfunction" OR "sexual dysfunctions" OR<br>erectile dysfunction[mesh] OR "erectile dysfunction" OR sexual satisfaction OR sex offenses[mesh] OR sexual violence OR gender-based violence[mesh] OR gender-based violence OR<br><br>family planning services[mesh] OR "Family Planning" OR contraceptive agents[mesh] OR contraceptive devices[mesh] OR "contraceptive" OR "contraceptives" OR Condom[mesh] OR condom OR condoms OR Infertility[mesh] OR infertility OR fertility[mesh] OR fertility OR prostatic neoplasms[mesh] OR "Prostate cancer" OR<br><br>Genital Diseases, Male[mesh] OR sexually transmitted diseases[mesh] OR "Sexually transmitted infections" OR STIs OR "Sexually transmitted diseases" OR STDs OR chlamydia[mesh] OR Chlamydia OR Gonorrhoea[mesh] OR gonorrhea OR gonorrhoeae OR Syphilis[mesh] OR Syphilis OR Trichomonas Infections[mesh] OR Trichomoniasis OR Herpes Genitalis[mesh] OR "herpes genitalis"[tiab] OR Papillomavirus Infections[mesh] OR "Papillomavirus" OR Condylomata Acuminata[mesh] OR "Genital warts" OR HIV Infections[mesh]) AND	1 034 552
**#2**<br>**men**	(men[mesh] OR Men OR man OR men's OR Male[mesh] OR male OR masculinity[mesh] OR masculinity OR Men's Health[mesh] OR Homosexuality, Male[mesh] OR "MSM" OR "men having sex with men"[TIAB] OR "men who have sex with men"[TIAB] OR "men who have sex with other men"[TIAB] OR<br>((transgender*[TIAB] OR transgender persons[MH] OR transsexual*[TIAB]) AND man) OR transman[TIAB] OR "trans men"[TIAB] OR transmen[TIAB]) AND	558 975
**#3**<br>**healthcare**	(Health Services Accessibility[mesh] OR Health Facilities[mesh] OR community health services[mesh] OR health services[mesh] OR health services research[mesh] OR delivery of health care[mesh] OR preventive health services[mesh] OR health services needs and demand[MH] OR quality of health care[majr:noexp] OR "Health care providers" OR<br><br>"Health care"[tiab] OR "Health services"[tiab] OR "Healthcare"[tiab] OR "health-care"[tiab] OR clinic[tiab] OR hospital[tiab] OR "primary care"[tiab]) AND	121 596
**#4 experiences**	(Professional-Patient Relations[mesh] OR Attitude of Health Personnel[mesh] OR Patient Satisfaction[mesh] OR Healthcare Disparities[mesh] OR patient acceptance of health care[MH] OR health care evaluation mechanisms[mesh] OR<br><br>"perception"[tiab] OR "perceptions"[tiab] OR "perceive"[tiab] OR "perceived"[tiab] OR "satisfaction"[tiab] OR "expectation"[tiab] OR "expectations"[tiab] OR "experience"[tiab] OR "experiences"[tiab] OR "evaluation"[tiab] OR "assessment"[tiab] OR "quality"[tiab] OR "trust"[tiab] OR "Shame"[tiab] OR "stigma"[tiab]) AND	93 209
**#5**<br>**Emperical studies**	(Epidemiologic Research Design[mesh] OR Qualitative Research[mesh] OR empirical research[mesh] OR<br>Surveys and Questionnaires[mesh] OR survey OR surveys OR questionnaires OR questionnaire OR Empirical OR Cross-Sectional Studies OR Cohort OR Case-Control OR Observational OR Registries OR analysis OR	79 428

For peer review only - http://bmjopen.bmj.com/site/about/guidelines.xhtml

	Clinical Trials OR Meta-Analysis OR “meta analysis” OR “systematic review” OR “scoping review” OR “literature review” OR “review of literature” OR<br><br>Qualitative OR “Grounded Theory” OR Interviews as Topic[mesh] OR “interviews” OR “interview” OR focus groups[mesh] OR “Focus group” OR “focus groups” OR themes[tiab]) AND	
**#6**<br>**Nordic countries**	(Scandinavian and Nordic Countries[mesh] OR Scandinavian[tiab] OR Denmark[tiab] OR Danish[tiab] OR Finland[tiab] OR Finnish[tiab] OR Norway[tiab] OR Norwegian[tiab] OR Sweden[tiab] OR Swedish[tiab] OR Iceland[tiab] OR Icelandic[tiab] OR Greenland[tiab])	1892

Search results 13th Mai 2020: 1892 articles

Published from 1st January 2010: 896 articles

2) Svemed+

(Sexual health[mesh] OR Reproductive health[mesh] OR sexually transmitted diseases[mesh] OR hiv infections[mesh] OR contraceptive agents[mesh] OR contraceptive devices[mesh] OR infertility[mesh] OR prostatic neoplasms[mesh] OR sex offences[mesh] OR erectile dysfunction[mesh]) AND
(men[mesh] OR male[mesh] OR masculinity[mesh]) AND
(Professional-Patient Relations[mesh] OR Attitude of Health Personnel[mesh] OR Patient Satisfaction[mesh] OR Healthcare[mesh] Disparities[mesh] OR patient acceptance of health care[mesh] OR health care evaluation mechanisms[mesh] OR Health Facilities[mesh] OR community health services[mesh] OR health services[mesh] OR preventive health services[mesh])

Search results 26th April 2020: 1696 articles

Published from 1st January 2010: 390 articles

For peer review only

Appendix 2: lista över inkluderade artiklar om mäns erfarenheter inom sexuell och reproduktiva hälso och sjukvård i de nordiska länderna

Authors (year)	SRH subject	Country	Study design	Aim of the study	Discussed concepts
**Åhman et al. (2012)** [72]	1	Sweden	Qualitative (interviews) 17 m	To explore men's expectations of routine ultrasound and experiences when soft markers were discovered.	Information, policy, organization of care, waiting times, satisfaction, control, autonomy, Support, power and responsibility
**Andersson et al. (2012)** [36]	1	Sweden	Qualitative (interviews) 20 k, 8 m	To investigate parents' experiences and perceptions of group antenatal care in different antenatal clinics in Sweden	Knowledge about the services, continuity of care, users' gender, information, hcps' knowledge/competence, Engaging men
**Andersson et al. (2016)** [61]	1	Sweden	Quasi randomized study (survey) 627 m	To identify expectant fathers' expectations regarding the content of antenatal care during pregnancy and to examine associations between expectations and social factors	Information, regard, engaging men, users' age
**Andersson et al. (2017)** [75]	1	Sweden	Quasi randomized study (survey) 239 m	To compare the experiences of fathers with two different models of antenatal care, group based antenatal care and standard antenatal care	Information, satisfaction, support, autonomy
**Armuand et al. (2012)** [62]	5, cancer	Sweden	Cross-sectional (survey) 328 k, 156 m	To investigate cancer survivors' perception of fertility-related information and use of FP options in connection with cancer treatment during reproductive age. An additional aim was to investigate the relationships between receiving fertility-related information and sociodemographic factors, diagnosis, and a pretreatment desire for children.	Information, users' age
**Armuand et al. (2015)** [31]	5, cancer	Sweden	Qualitative (interviews) 11 k, 10 m	To investigate newly diagnosed cancer patients ' experiences of fertility-related communication and their reasoning about the risk of future infertility.	Information, satisfaction, autonomy, time constrain, No body's responsibility, vulnerability, masculinity
**Armuand et al. (2017)** [83]	5	Sweden	Qualitative (interviews) 15 m	To evaluate how transgender men experienced FP aimed at oocyte cryopreservation in a pilot program, which was developed within an established university hospital-based FP program.	Referral possibility, waiting times, vulnerability, masculinity, satisfaction
**Åsenhed et al. (2014)** [37]	1	Sweden	Qualitative (internet bloggs) 11 m	To identify and describe the process of fatherhood during the partner's pregnancy among expectant, first-time fathers.	Women centred care, engaging men, users' gender, willingness to talk
**Bäckström et al. (2011)** [41]	1	Sweden	Qualitative (interviews) 10 m	To explore how first-time fathers describe requested and received support during a normal birth.	Communication, trust, support, engaging men, autonomy, users' as person

For peer review only - http://bmjopen.bmj.com/site/about/guidelines.xhtml

**Berg (2013)** [50]	6	Norway	Cross-sectional (survey) 2011 m	To identify prevalence of, and factors that are associated with, HIV testing	Users' age, users' education, users' knowledge, knowledge about the services, users' attitude, users' sexuality, confidentiality, regard, holistic care
**Bergengren et al. (2018)** [65]	Cancer	Sweden	Register based cross-sectional (survey) 1 288 m	To investigate overall satisfaction with care (OSC) and factors associated with OSC among men with low-risk prostate cancer (PC)	Satisfaction, autonomy, information, hcps' profession, users' age, users' education, waiting times, health outcome
**Berglund et al. (2012)** [66]	Cancer	Sweden	Population based cohort 17 522 m	To examine possible associations between socioeconomic status, metastatic work-up, treatment and mortality in patients with high risk pca managed in Sweden	Users' SES, health outcome
**Bjornshagen et al. (2020)** [54]	6	Norway	Cross-sectional (survey) 849 m	To illustrate who might benefit from HIV self-testing, by describing the characteristics of MSM who took an interest in the GLHN pilot project	Confidentiality, anonymity, health seeking behaviours, users' sexuality, users' age
**Bodin et al. (2018)** [68]	5	Sweden	Randomised controlled study (structured interviews) 201 m	To evaluate if Reproductive Life Plan (RLP)-based counselling during a sexual health visit could increase men's fertility awareness	Users' knowledge, information, satisfaction
**Brannstrom et al. (2016)** [10]	6	Sweden	Cross-sectional (survey) 372 m, 203 w	To identify factors in HIV-infected patients and the health care system which contribute to late diagnosis.	Users' age, users' ethnicity, health outcome, users' sexuality, users' gender
**Christianson et al. (2013)** [48]	6	Sweden	Qualitative (interviews) 20 m	To investigate how to prevent transmission of HIV and CT from a gender perspective by exploring whether screening of men during pregnancy may be an innovative way to reach men, to increase detection, and to avoid the present gendered responsibility.	Risk perception, engaging men, women centred healthcare, information, knowledge about the services, social norms, No body's responsibility, users' attitude
**Christianson et al. (2017)** [13]	6	Sweden	Qualitative (interviews) 20 m	To discursively explore expectant fathers' perceptions of chlamydia and HIV, and their masculinity constructions about testing, and explored how they talked about their potential resistance towards testing and their pre-test emotions.	Vulnerability, masculinity, health seeking behaviour, users' attitude/feelings
**Erlandsson and Häggström-Nordin (2010)** [84]	1, 2	Sweden	Qualitative (interviews) 15 m	To capture fathers' conceptions of parental education topics, illuminated by their experiences as primary caregiver of their child immediately following birth.	Role of men, engaging men, social norms
**Fabian et al. (2015)** [43]	1, 2	Sweden	Qualitative (interviews) 26 midwives	To explore antenatal care midwives' experiences and thoughts about the parental class activities provided during pregnancy.	Holistic care, clinical training, hcps' competence, Users' social capital, engaging men, users' gender

For peer review only - http://bmjopen.bmj.com/site/about/guidelines.xhtml

**Fridriksson et al. (2012)** [64]	2, cancer	Sweden	Register based cross-sectional (survey)<br>1 621 m	To assess the proportion of men subsequently diagnosed with prostate cancer who had received information prior to blood draw	Information, knowledge about the services
**Grandahl and Small (2019)** [17]	Other	Sweden	Qualitative (interviews)<br>22 midwives	To explore the thoughts and experiences of midwives working in the primary care setting concerning their preventive work for men's sexual and reproductive health and rights	No body's responsibility, hcps' attitude, Clinical training, hcps' knowledge, health seeking behaviour, organization of care, time constrains, women centred care, engaging men
**Grandahl et al. (2109)** [47]	6	Sweden	Qualitative (interviews) 33 m	To investigates boys' awareness and thoughts about human papillomavirus (HPV) and HPV vaccination, perceived benefits of vaccinating men, information sources and intention to be vaccinated against HPV	Users' attitude, willingness to participate, risk perception, information
**Hasman et al. (2014)** [45]	1	Denmark	Qualitative (interviews)<br>10 m	To describe how fathers experienced childbirth when nonprogressive labour occurred and augmentation was established.	Information, engaging men, role of men, control, hcps' competence, , security
**Herder and Agardh (2019)** [33]	6	Sweden	Qualitative (interviews)<br>10 m	To explore experiences and perceptions regarding communication about infectiousness and the rules of conduct with clinical staff at HIV clinics among MSM living with HIV in Sweden	Hcps' attitude, regard, trust, hcps' knowledge, security, , information
**Hildingsson and Sjöling (2011)** [58]	1	Sweden	Prospective longitudinal study (survey)<br>655 m	To describe personal and professional sources of support used by prospective and new fathers and to study factors associated with fathers having no support from anyone in mid-pregnancy.	Support, engaging men, users' social capital, willingness to participate, satisfaction
**Hildingsson et al. (2011)** [60]	1	Sweden	Cross-sectional (survey)<br>595 m	To identify the proportion of fathers having a positive experience of a normal birth and to explore factors related to midwifery care that were associated with a positive experience.	Satisfaction, support, information, users' age, users' ethnicity, users' education
**Holter et al. (2014)** [69]	5	Sweden	Cross-sectional (survey)<br>292 m,363 k	To investigate whether men and women differ in their evaluations of the importance of different aspects of quality of care, when measured by the use of the validated QPP-IVF instrument and to investigate if any baseline characteristics influenced the scores of subjective importance	Continuity of care, information, satisfaction
**Holter et al. (2017)** [81]	5	Sweden	Cross-sectional (survey)<br>268 IVF staff, 1435 m, 1863 k	To compare IVF healthcare professionals' estimates with patients' actual experiences of patient-centered quality of care measured with the QPP-IVF questionnaire, and investigate if certain factors influenced the IVF professionals' perceptions and IVF patients' experience of quality of care.	Satisfaction, continuity of care, autonomy, easy access, users' education, public vs. Private

For peer review only - http://bmjopen.bmj.com/site/about/guidelines.xhtml

**Hoyos et al. (2018)** [56]	6	Multinational	Cross-sectional (survey) 8 226 m (397 Danish)	To describe the knowledge about the existence as well as actual and potential use of self-sampling testing and to assess the acceptability of different result communication methods as well as the preferred sampling method among MSM recruited online in eight European countries.	Knowledge about the services, acceptability, communication
**Johansson and Hildingsson (2013)** [76]	1	Sweden	Cross-sectional (survey) 827 m	To explore Swedish fathers' intrapartum care quality experiences, with a specific focus on care deficiencies in relation to birth mode. A secondary aim was to explore which issues of quality that contributed most to dissatisfaction with the overall assessment of the care.	Satisfaction, support, autonomy, nature of the problem, information
**Johansson and Thies-Lagergren (2015)** [32]	1	Sweden	Mixed methods (survey, free texts) 221 m	To investigate how maternal birth position during second stage of labour may influence fathers' experience of childbirth	Satisfaction, nature of the problem, power, security, engaging men, support, willingness to participate, hcps' attitude, information, hcps' knowledge/competence, continuity of care, trust
**Johansson et al. (2011)** [15]	5	Sweden	Qualitative (interviews) 8 m	To describe men's experiences of obstructive azoospermia infertility	Vulnerability, masculinity, women centred care, engaging men
**Johansson et al. (2012)** [40]	1	Sweden	Mixed methods (survey, free texts) 827 m	To explore Swedish fathers' birth experiences, and factors associated with a less-positive birth experience.	Satisfaction, nature of the problem, users' education, hcps' competence, support, information, regard, autonomy, engaging men
**Johansson et al. (2013)** [73]	1	Sweden	Qualitative (interviews) 22 m	To describe and explore fathers' experiences of their partner's caesarean section birth.	Nature of the problem, information, hcps' Attitude, support, satisfaction, hcps' competence, control
**Johansson et al. (2014)** [80]	1	Sweden	Qualitative (interviews) 21 m	To explore and describe Swedish fathers' beliefs and attitudes around the decision for a caesarean section.	Shared responsibility, trust, information, nature of the problem
**Johnsen et al. (2017)** [38]	1	Multinational	Qualitative (interviews) 31 m Sweden, 8 m Denmark, 5 m Finland	To illuminate expectant first-time fathers' experiences of participation during pregnancy in three Nordic countries.	Willingness to participate, engaging men, social norms, responsibility
**Jungmarker et al. (2010)** [74]	1	Sweden	Cohort study (survey) 827 m	To describe expectant fathers' experiences of and involvement in prenatal care in Sweden.	Willingness to participate, satisfaction, holistic care, information, support, engaging men
**Kero et al. (2010)** [71]	7	Sweden	Qualitative (interviews) 23 couples	To gain knowledge about the male partner's experience of being present during an induced home abortion	Willingness to participate, autonomy, information, engaging men

For peer review only - http://bmjopen.bmj.com/site/about/guidelines.xhtml

**Klaeson et al. (2013)** [27]	8, cancer	Sweden	Qualitative (fgds) 19 m	To explore how men diagnosed with prostate cancer before the age of 65 years, in all stages, experienced and talked about changes in their sexuality due to cancer as a subgroup in the society	Vulnerability, masculinity, nature of the problem, social norms, Willingness to talk, hcps' gender, continuity of care, information, support
**Klaeson et al. (2017)** [16]	Other	Sweden	Qualitative (interviews) 9 nurses	To illuminate nurses' experiences and opportunities to discuss sexual health with patients in primary health care	Social norms, hcps' attitude, clinical training, organization of care, no body's responsibility, users' gender, clinical training, holistic care, users' age
**Koert et al. (2019)** [42]	1	Denmark	Qualitative (interviews) 11 couples	What do couples referred to or attending a Recurrent Pregnancy Loss clinic believe they need in terms of treatment, support and follow up?	Holistic care, regard, engaging men , information, continuity of care, referral possibility, organization of care
**Linnarsson et al. (2013)** [91]	4	Sweden	Cross-sectional (survey) 46 emergency departments	To describe the preparedness to provide care for victims of violence and their families in emergency departments (eds) in Sweden.	Women centred care, policy, organization of care, clinical training
**Makenzius et al. (2012)** [82]	7	Sweden	Cross-sectional (survey) 590 m, 798 k	To investigate satisfaction with abortion care among women and their male partners, and to identify factors associated with high overall care satisfaction	Satisfaction, regard, information, willingness to participate
**Makenzius et al. (2013)** [52]	7	Sweden	Qualitative (interviews) 24 k, 13 m	To explore women's and men's experiences and needs in relation to an induced medical abortion that involves carrying out the final treatment at home and to elicit their views on contraception and prevention of future unwanted pregnancies.	Autonomy, responsibility, continuity of care, privacy, control, information, regard, satisfaction, women centred care
**Marrone et al. (2016)** [53]	6	Sweden	Register based cross-sectional (survey) 1896 m, 950 k	To evaluate the Health Questionnaire and identify the main determinants of adherence	Satisfaction, autonomy, users' gender, users' ethnicity, health outcome
**Micaux Obol et al. (2017)** [30]	5, cancer	Sweden	Nationwide cross-sectional (survey) 329 oncologists and haematologists	To investigate the practice behaviors, attitudes, confidence in knowledge and perceived barriers to discussing fertility issues among physicians in cancer care and to identify factors related to physicians' practice behaviors regarding discussions about treatment-related fertility risks with female and male patients of reproductive age.	Information, users' age, no body's responsibility, referral possibility, workload, organization of care, hcps' knowledge
**Mikkelsen et al. (2013)** [26]	5	Denmark	Cross-sectional (survey) 210 m	To gain further knowledge about the experiences of infertile men for whom intracytoplasmic sperm injection treatment was the only way to establish fatherhood and to explore the psychological needs of the infertile man, focusing on communication in the clinic	Vulnerability, masculinity, engaging men, information , hcps' gender

For peer review only - http://bmjopen.bmj.com/site/about/guidelines.xhtml

**Oster et al. (2013)** [34]	Cancer	Sweden	Qualitative (conversational support group sessions) 9 m	To describe the shared experiences in a conversational support group of men with prostate cancer during a course of radiotherapy.	Satisfaction, nature of the problem, autonomy, waiting times, information, hcps' attitude, support, regard
**Persson et al. (2012)** [46]	1	Sweden	Qualitative (fgds, interviews) 20 m	To explore and describe factors, which influence fathers' sense of security during the first postnatal week.	Willingness to participate, information, security, hcps' knowledge/competence, organization of care, engaging men, support, regard, follow up
**Persson et al. (2016)** [49]	6	Sweden	Cross-sectional (survey) 2 373 m	To explore motivators and barriers to HIV testing and to assess factors associated with testing among MSM in the era of ART	Risk perception, users' knowledge, confidentiality, knowledge about the services, users' age, users' ethnicity, users' education
**Premberg et al. (2011)** [44]	1	Sweden	Qualitative (re-enactment interviews) 10 m	To describe fathers' experiences during childbirth	Information, willingness to participate, engaging men, support, autonomy
**Qvarnstrom and Oscarsson (2015)** [70]	6	Sweden	Cross-sectional (survey) 656 m	To describe experiences of and attitudes towards HIV/ STI prevention efforts prior to travel abroad among MSM and to investigate the kinds of prevention efforts that are desirable.	Availability of services, information, information
**Qvist et al. (2014)** [92]	6	Denmark	Cross-sectional (survey) 1 clinic	To evaluate a community based human immunodeficiency virus (HIV) testing program for its capacity to reach men who have sex with men (MSM) and successfully refer HIV-positive patients to treatment.	Easy access, follow up, referral possibility
**Rasmusson et al. (2013)** [63]	8, cancer	Sweden	Cross-sectional (survey) 54 k, 51 m	To investigate information about sexual effects of cancer on patients irrespective of age and diagnosis in terms of fertility, sexual desire and sexual function.	Information, users' gender, users' age
**Schildmeijer et al. (2019)** [77]	Cancer	Sweden	Qualitative (interviews) 14 m	1. Explore and describe how patients diagnosed with prostate cancer experience their journey through cancer care, by visualizing a typical patient journey and juxtaposing it with the SCP, and 2. Identify the patients' needs for support during the journey.	Information, waiting times, control, vulnerability, masculinity
**Schmidt et al. (2013)** [55]	6	Multinational	Cross-sectional (survey) 52430 m	To compare the performance of STI services used by MSM.	Knowledge about the services
**Schytt and Bergstrom (2014)** [59]	1	Sweden	Randomized controlled study (survey) 777 m	To investigate first-time fathers' expectations and experiences of childbirth and satisfaction with care in relation to paternal age.	Users' age, satisfaction, hcps' competence, support, trust

For peer review only - http://bmjopen.bmj.com/site/about/guidelines.xhtml

**Simmons et al. (2016)** [28]	4	Sweden	Qualitative (interviews) 12 m	To develop a theoretical model concerning male victims' processes of disclosing experiences of victimisation to healthcare professionals in Sweden.	Holistic care, fear, trust, confidentiality, masculinity, users' gender, hcps' gender, social norms, gender norms, support, regard
**Sollesnes (2010)** [5]	Other	Norway	Qualitative (fgds) 22 k, 10 m	To obtain insight to factors that can influence adolescent males and females' use of adolescent health clinics	Women centred care, help seeking behaviour, knowledge about the services, confidentiality, regard (patient as person)
**Stromdahl et al. (2017)** [51]	6	Sweden	Cross-sectional (survey) 244 m	To examine HIV-testing prevalence and uptake of HIV prevention interventions including different HIV-testing options among foreign-born MSM living in Sweden	Users' ethnicity, knowledge about the services, risk perception, easy access, anonymity, confidentiality
**Stromdahl et al. (2019)** [57]	6	Sweden	Cross-sectional (survey) 595 m	To evaluate the uptake of Testpoint (whether Testpoint achieved its aim of reaching MSM and trans persons, with a special focus on young and foreign born MSM)	Health seeking behaviour, users' ethnicity, users' age, knowledge about the services
**Sylvest et al. (2016)** [35]	5	Denmark	Qualitative (interviews) 10 m	To explore experience, expectations, needs, and assessment of fertility care among men with severe male-factor infertility.	Information, communication, waiting times, regard (patient as a person), willingness to participate, engaging men, holistic care
**Sylvest et al. (2018)** [67]	5	Denmark	Qualitative (interviews) 21 m	To explore men's expectations and experiences of fertility assessment and counseling through qualitative interviews conducted immediately before and some weeks after fertility counseling	Information, power, satisfaction, regard, masculinity, vulnerability
**Thies-Lagergren and Johansson (2019)** [79]	1	Sweden	Cross-sectional (survey) 209 couples	To describe and evaluate uniformity in couples' birth experience and experience of the quality of intrapartum midwifery care	Satisfaction, control, support,
**Vik and Brekke (2017)** [29]	Other	Norway	Cross-sectional (survey) 22 general practitioners 1 117 consultations	To shed some light upon how frequently and how Norwegian gps deal with concerns related to sexuality among their patients.	Prevalence of sexual problems presented to GP, hcps' competence, satisfaction
**Wibe et al. (2012)** [78]	2, cancer	Norway	Qualitative (online messages, interviews) 12 m	To explore how an online patient-nurse communication (OPNC) service meets the information needs of men with newly diagnosed testicular cancer	Information, support, control, communication, Waiting times
**Widarsson et al. (2012)** [14]	1	Sweden	Qualitative (fgds, interviews) 22 k, 10 m	To describe expectant mothers' and fathers' perceived needs of support during pregnancy	Holistic care, satisfaction, engaging men, women centred care, information
**Widarsson et al. (2015)** [39]	1	Sweden	Qualitative (fgds, interviews) 20 k, 10 m	To describe the perspectives of expectant mothers and fathers on fathers' involvement during pregnancy	Willingness to participate, engaging men, information, satisfaction

For peer review only - http://bmjopen.bmj.com/site/about/guidelines.xhtml

1. Antenatal, intrapartum and postnatal care
2. Comprehensive education and information
3. Contraception counselling and provision
4. Gender-based violence prevention, support and care
5. Fertility care
6. Prevention and control of HIV and other sexually transmissible infections
7. Safe abortion care
8. Sexual function and psychosexual counselling

For peer review only

For peer review only - http://bmjopen.bmj.com/site/about/guidelines.xhtml

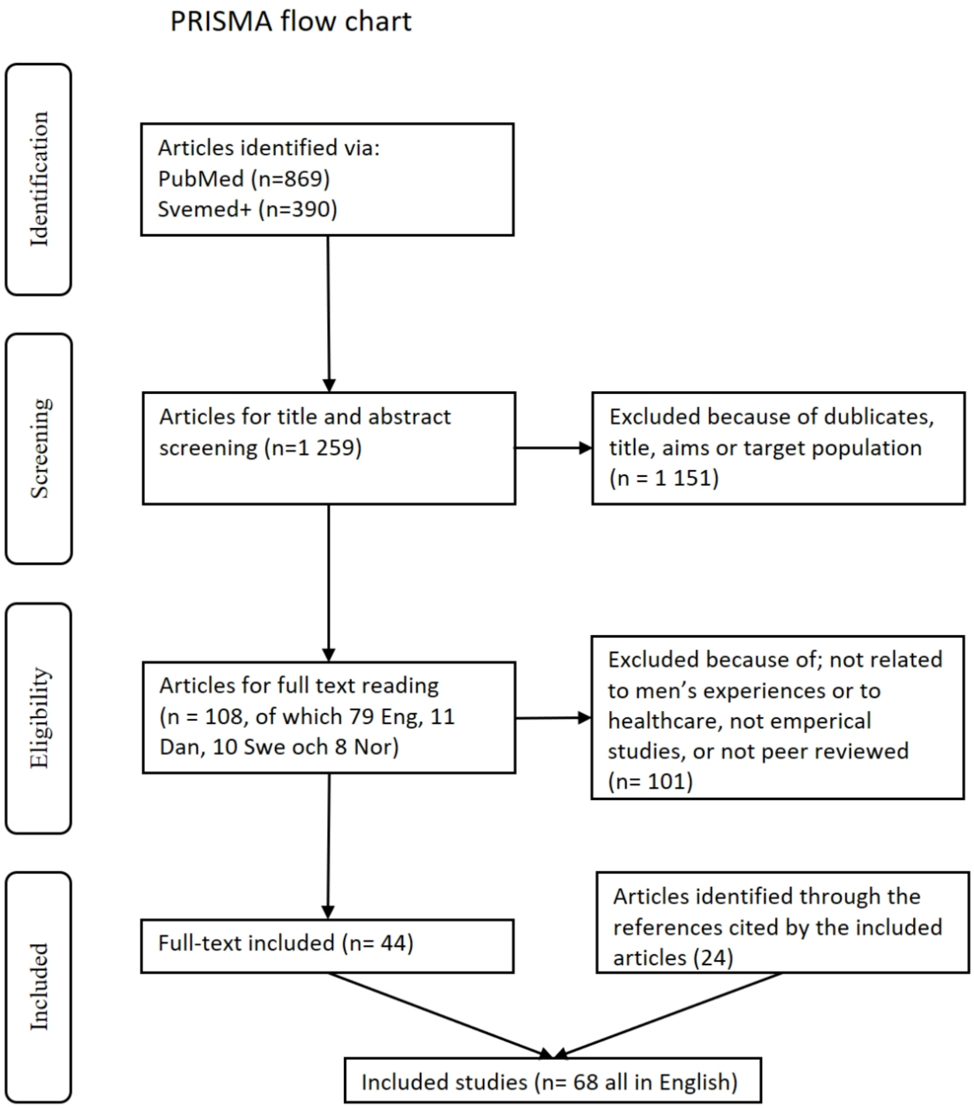

Figure 1: PRISMA flow chart on search results of men's experiences in sexual and reproductive healthcare in the Nordic countries.

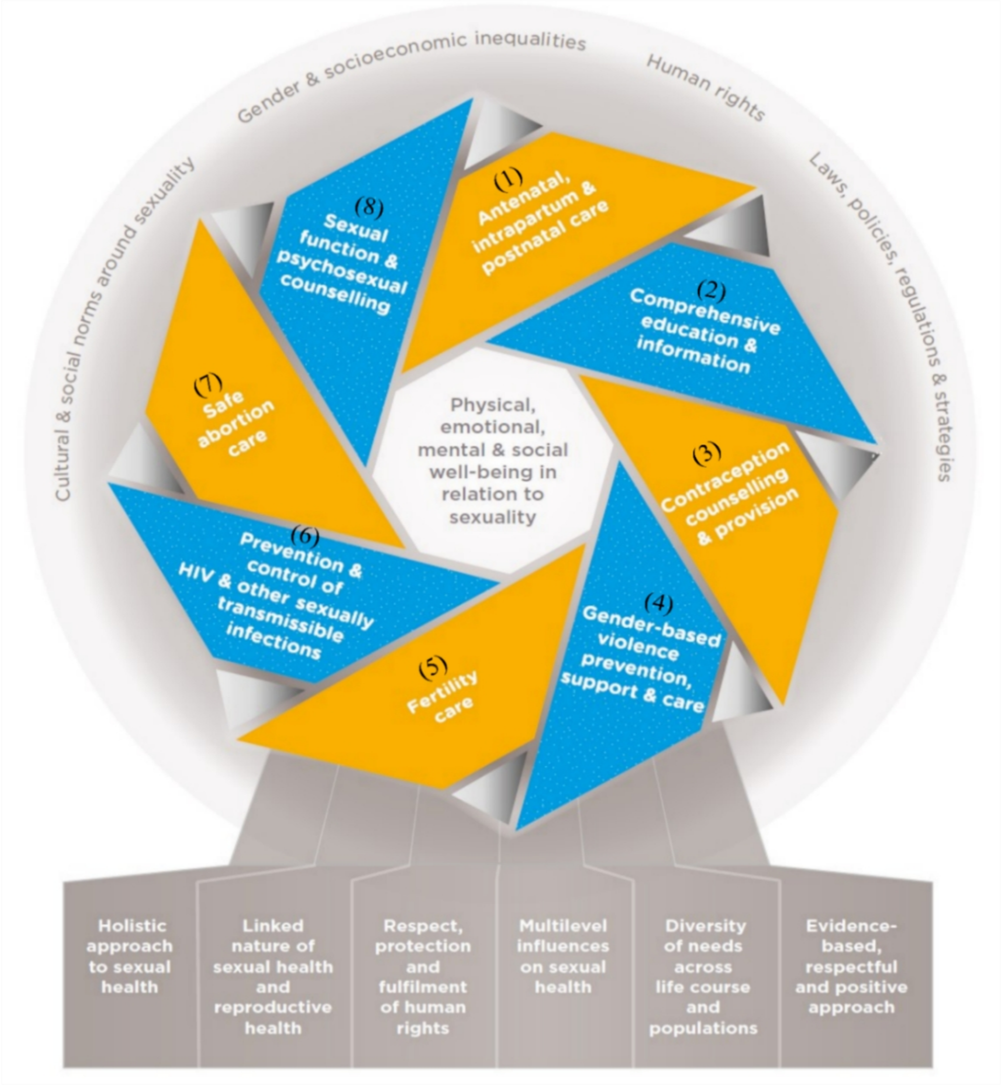

Figure 2: Framework for operationalizing sexual health and its linkages to reproductive health (from "Sexual health and its linkages to reproductive health: an operational approach").24 The intertwined blue and orange ribbons represent sexual health and reproductive health, respectively.

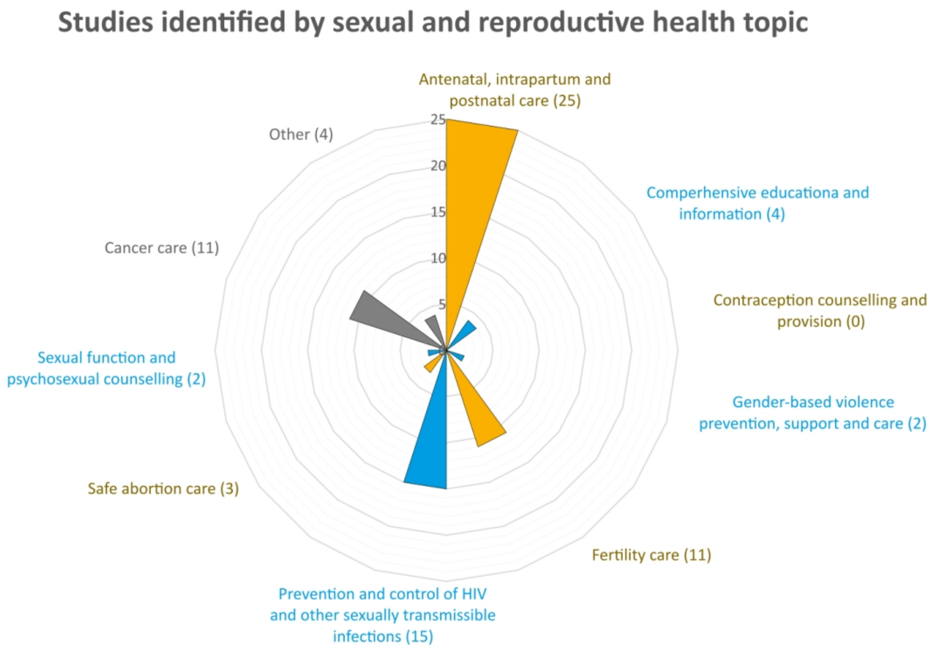

Figure 3: Men's experiences in sexual and reproductive healthcare in the Nordic countries. Number of studies identified grouped by sexual and reproductive health topics.

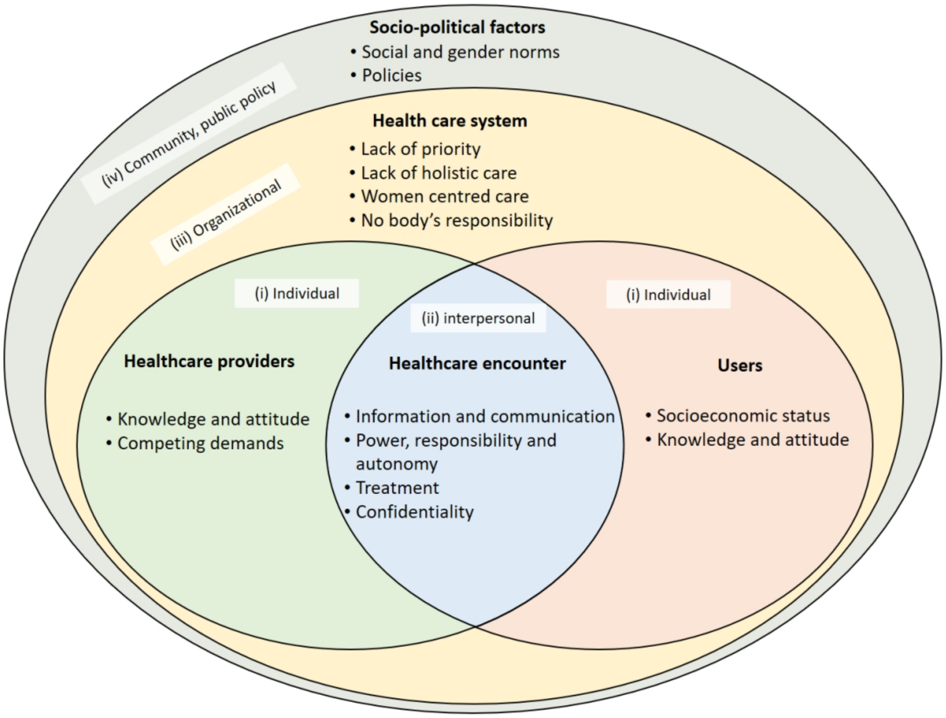

Figure 4: Theoretical framework for analysis of men's experiences in sexual and reproductive healthcare, adapted from Kilbourne et al.25

PRISMA flow chart

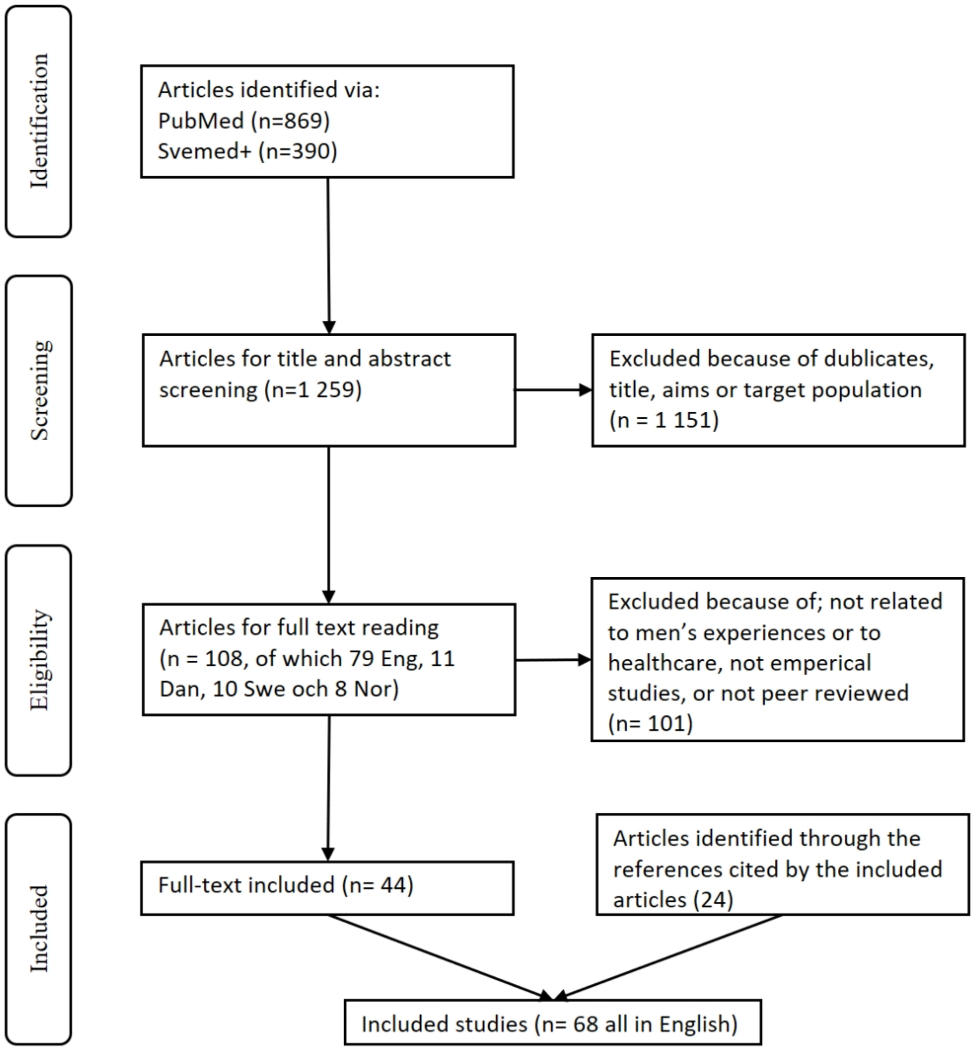

BMJ Open

Men and sexual and reproductive healthcare in the Nordic countries: a scoping review

Journal:	*BMJ Open*
Manuscript ID	bmjopen-2021-052600.R1
Article Type:	Original research
Date Submitted by the Author:	26-Aug-2021
Complete List of Authors:	Baroudi, Mazen; Umeå Universitet Medicinska fakulteten, Epidemiology and Global Health<br>Stoor, Jon Petter; Umeå Universitet Medicinska fakulteten, Department of Epidemiology and Global Health; UiT The Arctic University of Norway, Centre for Sami Health Research, Department of Community Medicine<br>Blåhed, Hanna; Umeå Universitet Medicinska fakulteten, Department of Epidemiology and Global Health<br>Edin, Kerstin; Umeå Universitet Medicinska fakulteten, Department of Epidemiology and Global Health<br>Hurtig, Anna-Karin ; Umeå Universitet Medicinska fakulteten, Department of Epidemiology and Global Health
**Primary Subject Heading**:	Sexual health
Secondary Subject Heading:	Public health, Health services research
Keywords:	PUBLIC HEALTH, Sexual dysfunction < UROLOGY, SEXUAL MEDICINE

SCHOLARONE™
Manuscripts

I, the Submitting Author has the right to grant and does grant on behalf of all authors of the Work (as defined in the below author licence), an exclusive licence and/or a non-exclusive licence for contributions from authors who are: i) UK Crown employees; ii) where BMJ has agreed a CC-BY licence shall apply, and/or iii) in accordance with the terms applicable for US Federal Government officers or employees acting as part of their official duties; on a worldwide, perpetual, irrevocable, royalty-free basis to BMJ Publishing Group Ltd ("BMJ") its licensees and where the relevant Journal is co-owned by BMJ to the co-owners of the Journal, to publish the Work in this journal and any other BMJ products and to exploit all rights, as set out in our licence.

The Submitting Author accepts and understands that any supply made under these terms is made by BMJ to the Submitting Author unless you are acting as an employee on behalf of your employer or a postgraduate student of an affiliated institution which is paying any applicable article publishing charge ("APC") for Open Access articles. Where the Submitting Author wishes to make the Work available on an Open Access basis (and intends to pay the relevant APC), the terms of reuse of such Open Access shall be governed by a Creative Commons licence – details of these licences and which Creative Commons licence will apply to this Work are set out in our licence referred to above.

Other than as permitted in any relevant BMJ Author's Self Archiving Policies, I confirm this Work has not been accepted for publication elsewhere, is not being considered for publication elsewhere and does not duplicate material already published. I confirm all authors consent to publication of this Work and authorise the granting of this licence.

For peer review only

Men and sexual and reproductive healthcare in the Nordic countries: a scoping review

Mazen Baroudi* mazen.baroudi@umu.se

+46– 90 786 75 54 Department of Epidemiology and Global Health, Umeå University, 901 87 UMEÅ

Jon Petter Stoor, petter.stoor@umu.se

Department of Epidemiology and Global Health, Umeå University, Umeå

Centre for Sami Health Research, Department of Community Medicine, UiT – the Arctic University of Norway, Tromsø, Norway

Hanna Blåhed hanna.blahed@umu.se

Department of Epidemiology and Global Health, Umeå University, Umeå

Kerstin Edin kerstin.edin@umu.se

Department of Epidemiology and Global Health, Umeå University, Umeå

Anna-Karin Hurtig anna-karin.hurtig@umu.se

Department of Epidemiology and Global Health, Umeå University, Umeå

*corresponding author

For peer review only

Men and sexual and reproductive healthcare in the Nordic countries: a scoping review

Abstract

Context

Men generally seek healthcare less often than women and, other than traditional gender norms, less is known about the explanation. The aim was to identify knowledge gaps and factors influencing men regarding sexual and reproductive healthcare (SRHC) in the Nordic countries.

Methods

We searched PubMed and SveMed+ for peer-reviewed articles published between January 2010 and May 2020. The analyses identified factors influencing men's experiences of and access to SRHC.

Results

The majority of the 68 articles included focused on pregnancy, birth, infertility, and sexually transmitted infections including HIV. During pregnancy and childbirth, men were treated as accompanying partners rather than individuals with their own needs. The knowledge and attitudes of healthcare providers were crucial for their ability to provide SRHC and for the experiences of men. Organizational obstacles, such as women-centred SRHC and no assigned healthcare profession for men's sexual and reproductive health issues, hindered men's access to SRHC. Lastly, the literature rarely discussed the impact of health policies on men's access to SRHC.

Conclusions

The literature lacked the perspectives of specific groups of men such as migrants, men who have sex with men (MSM) and transmen, and the experiences of men in SRHC related to sexual function, contraceptive use and gender-based violence. These knowledge gaps, taken together with the lack of a clear entry point for men into SRHC indicate the necessity of an improved health and medical education of healthcare providers, as well as of health system interventions.

Keywords

Sexual and reproductive healthcare, men, experience, Nordic countries, gender norms, right to healthcare

Strengths and limitations of this study

- This review is the first to examine the experiences of men in SRHC in the Nordic countries.
- We used of a Nordic-specific database without restriction to language.

For peer review only - http://bmjopen.bmj.com/site/about/guidelines.xhtml

- Search was restricted to two databases but complemented with manually screening the reference lists of the identified literature.
- The broad nature of the field and the wide variety of terms related to sexual and reproductive health make it difficult to assure the inclusion of all relevant literature.
- We implicitly treated the Nordic countries as essentially similar, which might obscure important differences between or within countries.

For peer review only

For peer review only - http://bmjopen.bmj.com/site/about/guidelines.xhtml

Introduction

Addressing men's sexual and reproductive health (SRH) needs alongside that of women's is essential, however men´s SRH is neglected. Men generally seek healthcare, especially primary healthcare, to a lower degree than women, and this also applies to sexual and reproductive healthcare (SRHC).[1,2] For example, 23% of over 40 year-old men in Europe reported sexual dysfunction but only one-quarter of them sought healthcare,[3] and similar results have been reported in Sweden.[4] Also, studies from Sweden and Norway have indicated that youth clinics are perceived as "women clinics". Therefore, fewer men seek these services compared to young women.[5,6] Additionally, although not universal, men test themselves for sexually transmitted diseases to a lower extent compared to women.[7,8] However, various groups of men might have different health seeking behaviours and different experiences in SRHC. For example, men with high socioeconomic status[1,9] and men who have sex with men (MSM) seek SRHC more often.[10,11] The higher use of SRHC among MSM might be due to their higher needs. Furthermore, MSM experiences in SRHC might differ due to their level of openness about their sexual orientation and related to structural factors such as homophobia.[10,11]

Traditional gender norms might urge men to be independent, strong and invulnerable and also hinder them from acknowledging having problems, creating a barrier to seeking healthcare.[12] In particular, admitting sexual health problems might imply more vulnerability for men, thus decreasing the likelihood of seeking healthcare.[5,13] Even though gender norms play an important role in men's health seeking behaviours, it cannot alone explain the lower utilization of SRHC. Men who eventually sought SRHC did not get the help they expected. For example, more than half of men who sought help related to sexual function in Sweden reported not getting enough support.[4] Furthermore, men often felt excluded in healthcare related to infertility and pregnancy.[14,15] These experiences might be due to the lack of response of the health system to men's needs that can be related to healthcare organization and delivery,[9] including no support or guidelines for health professionals to promote men's SRH.[16] Additionally, health and medical education in Sweden, as an example, does not have enough focus on men's SRH.[16,17]

The Nordic countries are among the best in the world in the available international gender equality statistics.[18] Since gender inequality affects women's sexual and reproductive health (SRH) to a larger degree compared to men,[19,20] there is a greater focus on women's rights to SRH. Men's SRH does not get the same attention in practice and little is known about men's SRH in the Nordic countries.[21] The available literature mainly focuses on gender norms and masculinities and its link to health seeking behaviours and risk taking, while much less is known on how men are experiencing SRHC.[9,21-23]

Aim

The aim of this scoping review was to identify knowledge gaps and factors influencing men regarding SRHC in the Nordic countries during the period between 2010 and 2020.

For peer review only - http://bmjopen.bmj.com/site/about/guidelines.xhtml

Method

This review was performed according to Arksey and O'Malleys' method stages for conducting a scoping review, which includes identifying the research question, literature search, study selection, charting and synthetizing.[24] The research questions included: 1) What is the current status of the literature published in Scandinavian regarding men and SRHC? 2) How men in the Scandinavian countries are experiencing SRHC?

Search strategies and selection criteria

A structured search of the literature was conducted using two databases, PubMed and SveMed+ (a Scandinavian database) without restriction of language. Search terms included sexual and reproductive health, men, healthcare, experiences, and Nordic countries (see Appendix 1 for detailed search terms). The following eligibility criteria were used: (1) peer-reviewed empirical studies, all study designs were considered; (2) published between January 2010 and May 2020; (3) assessing men's experiences in SRHC or perspectives of HCPs on men's SRHC; and (4) conducted in the Nordic countries.

The initial search gave 1286 articles (896 from PubMed and 390 from SveMed+). After screening the titles and abstracts, 108 articles were read in full, and after being judged for their eligibility, 44 articles remained. An additional 24 papers were identified through the reference lists of these papers, resulting in 68 papers included in this scoping review (Figure 1). The articles were judged for eligibility by the first author, but when uncertainties arose, two co-authors read and judged the articles for eligibility separately. The three researchers then discussed the articles and decided unanimously on the inclusion/exclusion of these articles.

Data extraction and synthesis

The identified articles were mapped using the World Health Organization (WHO) framework for operationalizing SRH,[25] and the result part of each article were extracted and coded using sensitizing concepts of healthcare experiences (Appendix 2). Thereafter, the results were synthesized using a theoretical framework, adapted from Kilbourne et al., which provides health service research perspectives on understanding health and healthcare disparities.[26]

Patient and Public Involvement

Patients and the public were not involved in this study.

Figure 1: PRISMA flow chart on search results of men's experiences in sexual and reproductive healthcare in the Nordic countries.

For peer review only - http://bmjopen.bmj.com/site/about/guidelines.xhtml

Results

Description of the identified studies

Despite not restricting the language of the studies, all the 68 studies included were in English. The absolute majority of the studies were conducted in Sweden (54 articles), while six studies were conducted in Denmark, five in Norway and three in more than one country. No studies were identified from Iceland or Greenland.

Half of the studies (34) adopted a qualitative design, 32 studies a quantitative design and two studies a mixed methods design. Most of the studies (61 articles) were about men's perspectives of SRHC, while only seven studies covered the perspectives of healthcare providers (HCPs). Of the studies dealing with men's perspectives, 16 studies assessed women's perspectives together with that of men. Apart from two articles about the experiences of transgender men, the articles did not mention gender identities. Most of the papers dealing with men's perspectives referred to the overall experience of healthcare and healthcare staff in general. Of the 28 papers referring to specific primary healthcare providers, 14 mentioned midwives, eight mentioned physicians and six mentioned nurses.

SRH topics were grouped with help of the WHO framework for operationalizing sexual health and its linkages to reproductive health.[25] This framework was used because it demonstrates the interlinked nature between sexual health and reproductive health, yet clearly distinguish topics for intervention and research in both sexual health and reproductive health (Figure 2). Besides the eight topics from this framework, SRH cancers were also added, while the remaining studies with no one topic of focus were grouped under "other".

More than one-third of the papers were about the experiences of fathers/expectant fathers during antenatal, intrapartum and postnatal care (25 papers, including 12 about antenatal care and 11 about intrapartum care), while 15 papers dealt with sexually transmitted infections (STIs), mainly HIV (12 papers) and MSM (nine papers). We found 11 papers concerning men's experiences in infertility care (three of them were related to infertility among cancer patients) and eleven papers in cancer care. We also found four studies dealing with sexual education and information (two of them related to cancer and the other two related to antenatal care), three studies about abortion care, two studies about sexual violence and two studies about sexual functioning and counselling (both related to cancer patients). We found no study dealing with the provision of men's contraceptive counselling (Figure 3).

Figure 2: Framework for operationalizing sexual health and its linkages to reproductive health (from "Sexual health and its linkages to reproductive health: an operational approach").[25] The intertwined blue and orange ribbons represent sexual health and reproductive health, respectively.

Figure 3: Men's experiences in sexual and reproductive healthcare in the Nordic countries. Number of studies identified grouped by sexual and reproductive health topics.

For peer review only - http://bmjopen.bmj.com/site/about/guidelines.xhtml

Theoretical framework for analysis

The identified literature dealt with men's experiences in SRHC from various perspectives and can be organized in the framework adapted from Kilbourne et al.[26] Kilbourne et al. framework provides a multi-level approach to understand healthcare disparities. It provides an ecological lens that goes beyond individual to interpersonal and organizational factors. The factors influencing men's experiences are divided into (i) individual, including healthcare providers and users; (ii) interpersonal, which deals with the healthcare encounter and contact circumstances; (iii) organizational, which deals with healthcare system factors; and (iv) the larger influence of the community and public policies (Figure 4).

Figure 4: Theoretical framework for analysis of men's experiences in sexual and reproductive healthcare, adapted from Kilbourne et al.[26]

1. Healthcare providers' factors

The literature described how factors related to HCPs, such as sex, attitude, knowledge and competence, affect the HCP-user relationship and experiences of men in healthcare. For example, female HCPs did not prevent men from talking about their concerns regarding infertility.[27] Similarly, men diagnosed with prostate cancer wished to talk about sexuality with a mature knowledgeable HCP, without considering their sex.[28] Furthermore, disclosing victimization to female HCPs as compared to male HCPs was claimed to be easier for some men.[29]

1.1. Varied levels of knowledge, competing demands and differing attitudes

Lacking knowledge about men's SRH was expressed by various HCP professions and were associated with less ability to deal with men's SRH consultations. For example, nurses perceived that their lack of knowledge was influencing their preparedness to provide sexual health consultations for men.[16] Midwives also expressed their limited knowledge about male SRH, which was considered essential if inviting men for SRH consultations.[17] Also, less experienced physicians (young and/or under training) felt uncomfortable dealing with sexual health consultations.[30] Moreover, the competing demands in the form of high workload and limited time hindered HCPs from discussing SRH with men.[16,31,32]

Differing attitudes towards health seeking behaviours of men were found. While most HCPs were described as having positive attitudes, being friendly, sensitive and supportive,[33-36] some were still perceived as harsh and nonresponsive.[33,34] These negative attitudes were sometimes perceived by men as discrimination based on their sex, which hindered them, for example, from disclosing victimization.[29]

1.2. The view of men in reproductive healthcare services, an accompanying partner or an individual?

Even though HCPs in reproductive healthcare services usually deal with couples having a common reason for visiting healthcare, in most cases, they have primarily communicated with the women.[27,33,37,38] Women were in focus during infertility treatment, pregnancy and birth, leading men to feel neglected, invisible and superfluous during the visits.[14,15,39,40] The lack of interest in listening to or interacting with men also hindered their involvement in supporting their partners, for example, when giving birth.[41,42]

For peer review only - http://bmjopen.bmj.com/site/about/guidelines.xhtml

For peer review only

The lack of focus on men might be explained by time constraints and no time being allocated to men's concerns during visits.[17] Anyhow, the attitudes and behaviours of HCPs generally made a difference in men's perception of their involvement or lack of involvement in healthcare.[39] Couples highlighted the need to treat partners on equal terms and to focus on them as a unit rather than solely on the women,[14,15,27,37] and they expected communication as inclusive with both partners.[36] Men also expressed that HCPs should welcome them to more active involvement during birth and support their role as expectant fathers. HCPs should acknowledge men's needs and give them the opportunity to talk about their concerns.[14,27,33,43]

Examples of good practices involving men in reproductive healthcare are also mentioned in the literature. One example was participatory parental classes or separate parental classes for men and women dealing with men's concerns related to pregnancy and birth, which helped men to take part and to feel involved.[40,44] Another example was assigning tasks and continuously informing men during labour and allowing the father to stay at the hospital after the baby is born. These practices gave men a feeling of being important and recognized, hence receiving needed support.[42,45-47]

2. Healthcare users' factors

The literature described users' factors that influence their experiences in healthcare. This included men's socioeconomic situation, including education, age, knowledge and attitude. For example, the ages of users were discussed in relation to the ages of HCPs; nurses were more comfortable talking about sexuality with younger men as compared to men of their own age or older.[16] Young men, in comparison to young women, were pointed out as being less acquainted with youth clinics or where else to seek SRHC.[5]

Healthcare users' factors are discussed in the literature mainly in three SRH subject areas, namely, prevention and control of HIV and other STIs, antenatal/ intrapartum care and cancer care, which is elaborated on below.

2.1. Prevention and control of HIV and other STIs

Most of the literature focused on HIV testing, treatment and their sociodemographic determinants. See Box 1 for more details about the factors discussed in the literature.

The literature relating to other STIs (besides HIV) was limited to the attitudes of upper secondary school boys (median age=18) toward HPV vaccination and attitudes of men toward STI testing during the pregnancies of their partners. Upper secondary school boys had a positive attitude with regard to participation in HPV vaccinations; they stated that vaccinating only girls is unfair. Even though they had a positive attitude to share the responsibility of STI prevention, boys rarely used condoms, especially if they knew their sex partner in advance.[48] Men's attitudes toward STI testing during pregnancy were diverse. Some men perceived the test as an "infidelity check" that is sensitive and can risk the relationship, while others perceived it as a safety measure that should be "routine" during pregnancy.[13,49]

For peer review only - http://bmjopen.bmj.com/site/about/guidelines.xhtml

Box 1**: Key characteristics of users in relation to HIV testing and treatment**

- **Age**: Younger age was reported to be associated with higher HIV testing among men who have sex with men (MSM)[50,51] and earlier diagnosis in the general population.[10]
- **Education**: A lower level of education was associated with less testing for HIV in the general population,[51] but not among MSM.[50]
- **Country of birth**: Studies showed that country of birth was not associated with lower HIV testing among MSM.[50,52] However, two-thirds of the foreign-born HIV patients had not been tested for HIV at migration to Sweden.[10,53] Therefore, foreign-born men were more likely to be diagnosed late (65% of foreign-born compared to 43% of Swedish-born) and less likely to optimally adhere to HIV treatment.[10,54]
- **Sexuality**: Since HIV testing was perceived as implicitly implying same sex sexual relations, non-disclosing MSM were more likely to have been never tested for HIV.[51,55,56] However, MSM were less likely to be diagnosed late (40% of MSM compared to 67% of heterosexual patients) and less likely to optimally adhere to HIV treatment.[10,54]
- **Knowledge**: Men's knowledge about HIV transmission was associated with never being tested for HIV among MSM and the general population.[50,51] Never being tested for HIV was also associated with not knowing if the tests were free or affordable[51,57] and lack of knowledge about HIV testing services.[45,50,52] For example, only one-fourth of MSM knew about home sampling (Internet ordered tests),[58] and around 40% have never heard of the Testpoints programme (peer-led testing performed in MSM clubs, among other places).[59]
- **Risk perception**: The perception of having a very low risk of contracting STIs, including HIV, was highly associated with never being tested for HIV or STIs.[3,7,49,52]

2.2. Antenatal and intrapartum care

The literature discussed men's socioeconomic characteristics, knowledge and experiences in antenatal and intrapartum healthcare. Lack of knowledge about antenatal services, such as antenatal classes, was common among men; they usually had not heard about the service before but received information from their partners.[37] Men who had no social support from family and friends during the pregnancies of their partners were more dissatisfied with antenatal care and less likely to attend parental classes.[60] Studies found younger age and higher education level were associated with lower satisfaction with the overall birth experience,[41,61] while no such association was reported in relation to men's country of birth.[62] Additionally, younger men as compared to older men, perceived midwives as less supportive, less attentive and as not inspiring confidence.[61] These differences might be explained by younger men having higher expectations.[63]

2.3. Cancer care and SRH

The literature explored the factors of users related to cancer care, especially the effects of cancer treatment on fertility and sexuality. The majority of physicians claimed that they discussed the impact of cancer treatment on fertility if the patient was at reproductive age. However, one-third of the physicians did not do this regularly.[31,64] Around half of men in the 41–60 years old age group

For peer review only - http://bmjopen.bmj.com/site/about/guidelines.xhtml

claimed that they had not received enough information about the effects of cancer treatment on sexual desire, sexual function and fertility.[65]

Similar to other SRH services, lack of knowledge about the services was common, with only around one-fifth of men knowing about the PSA test for prostate cancer screening before testing.[66] Studies showed no associations between age and the overall satisfaction with cancer care, while a higher level of education was associated with lower overall satisfaction with prostate cancer care.[67] Furthermore, the literature indicated that manual workers were less likely to receive a bone scan and radical prostatectomy, and they had higher overall and cancer-specific mortalities as compared to non-manual employees.[68]

3. Healthcare encounter factors

The factors under which the healthcare encounters took place influenced the HCP-user relationship and experiences of users. The literature discussed, among other issues, HCP-user communication and the power and autonomy of men.

3.1. Information and communication

Information and communication were recurring themes in all SRH subject areas. More than half of the studies touched upon some aspect of information, or the way it is delivered and communicated. Receiving information was described as valuable and important and made men feel pleased, satisfied and empowered.[53,69-74] During the birth process, for example, information helped men to feel included and to find their place in supporting their partners and facilitated the decision-making of couples.[33,43,46,69] Contrarily, lack of sufficient information was associated with more concerns and feelings of exclusion and dissatisfaction.[41,75] Insufficient information was reported in various healthcare settings, for example, the effects of antenatal care,[37,76-78] infertility care[71] and cancer treatment/surgery on sexual health.[65,79]

The literature also discussed the format of information. Oral information was especially preferred when the matter aroused many questions, such as communicating an infertility diagnosis[36] or HPV vaccination,[48] while written information was considered more suitable in other cases, such as HIV and STI information for MSM.[72] However, even though recommended by the National Board of Health and Welfare in Sweden, studies have shown that the majority of men did not receive written information about prostate cancer screening and some were not even aware that they underwent the screening.[66] In other cases, a combination of oral and written information was considered easier to comprehend, for example, when communicating the side effects of cancer treatment on fertility[35] (see Box 2).

For peer review only - http://bmjopen.bmj.com/site/about/guidelines.xhtml

Box 2: **The charactersitics of satisfying information and communication - men's views**

- **Clear and simple language**: Clear and proper level information were perceived as important. The inability to understand the medical language of HCPs caused distress.[33,35,45,69]
- **Reliable**: Contradictions, unrealistic information and lack of reliable information caused frustration.[34,43] Exaggerated information (i.e. under- or overstating the real situation) was associated with unease, confusion and a sense of not being taken seriously.[53] Men wanted to feel welcome when asking questions and wanted honest, consistent and clear answers.[42,43] Men expressed a need for help to choose reliable websites and organize and discuss the information received.[14]
- **Personalized and relevant**: While general information could be obtained from the Internet, receiving personalized and relevant information from the HCPs was a high priority[27,47,69] For example, an online patient-nurse communication service played a central role in providing personalized information for cancer patients.[80]
- **Comprehensive and sufficient**: Receiving adequate and comprehensive information was regarded as important.[53] For example, men highlighted the need for a deeper dialogue about personal experiences or the psychological concequences of male infertility,[27] as well as psychological support during waiting times for cancer treatment.[79]
- **Appropriate and interactive**: The way HCPs communicated the information affected men's feelings; a possitive attitude and "a good mood" among HCPs mirrored less stress in men.[75] Having time to ask questions and interact with HCPs was also appreciated.[32]
- **Timely**: Constant updates of information during their partner's labour and birth was highly appreciated by men. Men who received timely information felt well informed, calm, secured and satisfied.[33,42,62,75] On the other hand, receiving information at inappropriate times was perceived as insufficient.[79]
- **Inclusive**: Involving men in the communication as an equal partner in reproductive healthcare was perceived as necessary.[27]

3.2. Lack of control and compromised autonomy in reproductive healthcare

Men's engagement in reproductive healthcare seemed to be a complex matter; midwives valued men's involvement, to a certain point, since they experienced over involvement as a possible sign of controlling behaviour or intimate partner violence.[17] The literature discussed men's involvement and their lack of control and compromised autonomy in various situations in reproductive healthcare, especially during pregnancy and birth. For example, the inability to help or act during their partner's birth made men experience lack of power and control.[46,75,81] Similarly, the uncontrollable process of non-progressing delivery left men with a feeling of helplessness and insecurity.[46] Men appreciated being involved in the decision regarding their partner's elective or emergency caesarean section, but 40% of the men felt they were not involved enough.[78,82] Also, men reported being more in control and more involved in decision-making during an elective caesarean section or normal spontaneous vaginal birth as compared to emergency caesarean section or assisted vaginal birth.[75,78,82] However, they also described situations where they were forced to participate in tasks and rituals without their consent, (i.e., cutting the umbilical cord or touching the child's head before the baby was

For peer review only - http://bmjopen.bmj.com/site/about/guidelines.xhtml

born).[45] Even though involvement in decision-making during birth was associated with higher satisfaction,[41,78] it was still important to be able to choose whether to participate or not in different stages of birth.[42]

Compromised autonomy was also reported in the infertility clinic[83] and when banking sperm before cancer treatment.[32] To the contrary, control and involvement in decisions were more satisfactory during home abortions. The pregnant woman made the decision, but the partner's opinion was important for her.[53,73]

3.3. Good treatment increases security and satisfaction

Men wanted HCPs to treat them as persons, respecting their needs, feelings and experiences. HCPs should try to understand the unique situation of each man and take it seriously.[36,42,47,53] Respectful treatment was highly expected and associated with higher satisfaction with care.[63,84] It was especially important to deliver negative news with sensitivity.[43,69] Men who experienced HCPs as professional, empathetic and attentive, felt satisfied, important and "not just a number".[5,36,51,53] In other cases, men perceived insensitivity and lack of respect or attention in the comments of HCPs, resulting in feeling disappointed and dissatisfied.[41,53,69]

The support of midwives during antenatal, intrapartum and postnatal care was necessary and created a feeling of security and satisfaction. Providing attention and information and addressing men's needs and questions helped men to build trust in the midwives and be supportive to their partners.[42,47,62,75-78] However, men were not always satisfied with the support of midwives, which made men feel insecure, helpless and worried.[41,78,81]

3.4. Confidentiality, a prerequisite to access to SRHC

Confidentiality was considered an essential condition to access certain SRH services, including youth clinics and HIV testing. For example, fear of being recognized in the clinic was one of the main reasons for not being tested for HIV.[50] Getting an HIV test was considered as implicitly disclosing same-sex sexuality, which led to preferring self-testing as an anonymous alternative, especially among non-gay MSM and those who had never been tested for HIV.[55] Therefore, anonymous HIV testing outside the healthcare system were requested and considered helpful for MSM.[52] Similarly, young people visiting youth clinics expressed the importance of HCPs' confidentiality and that they are used to and only work with young people.[5] Trust in HCPs' confidentiality was also described as important in the process of men disclosing victimization.[29]

4. Healthcare system factors

The healthcare system influenced the HCP-user relationship through its effects on HCPs and the healthcare encounters. Among other issues, the literature discussed the organization of healthcare, the holistic approach (or the lack of it), SRHC as traditionally women-centred care and men's SRHC as "nobody's mandate".

4.1. Men's SRH is not a priority

The literature indicated that the clinical training and organization of care does not give men's SRH enough priority. Nurses, for example, highlighted the lack of basic medical training and organizational support to deal with men's sexual health issues. Their main source of knowledge about men's SRH was received from pharmaceutical companies.[16] Similarly, midwifery education and

For peer review only - http://bmjopen.bmj.com/site/about/guidelines.xhtml

clinical training doesn't regularly include andrology, which together with lack of time and organizational support hindered them from providing counselling to men.[17]

Another example of the low priority of men's SRH was the lack of follow-up and continuity of care, which was reported in various services. For example, men reported not being followed-up after being prescribed medication for sexual function.[28] Additionally, the stays of men with the family after delivery was not welcomed in some hospitals, even though this was important for men in order to feel supported and to support their new family.[47]

The lack of prioritizing men's SRH was also reflected by the few prevention activities that healthcare performs regarding men's SRH. For example, the vast majority of MSM did not encounter any HIV/STI prevention services, despite the importance of making it more available.[72] Another example was the missed opportunity to counsel for sexual health in around one-third of men testing for HIV.[51]

4.2. Lack of holistic care

The literature discussed the lack of holistic care in SRH services. For example, psychological aspects of infertility were usually not acknowledged and therefore overlooked.[36] For couples with repeated pregnancy loss, psychological counselling was restricted to a few with certain criterion and also without considering individual situations.[43] Furthermore, antenatal care was perceived to focus mainly on medical support and rarely on emotional and psychological support, leaving only few users being very satisfied with this aspect of antenatal care.[14,76] Consequently, men who were subjected to gender-based violence were less likely to seek help unless they had severe physical injuries.[29]

4.3. Women-centred reproductive healthcare, a compromised right for inclusion of men

Both men and women expressed a wish to include men and to focus on "the couple" rather than one partner, that is, equal partners sharing a common reason for visiting reproductive healthcare.[15,36,43,53] Even though men felt that the focus on women in reproductive healthcare is reasonable, they stated that this attention should not exclude men.[36,49] The feeling of exclusion was experienced by men in different reproductive health services, including fertility and antenatal care.[14,15,38,49] One study showed that the investigations and treatments focused only on the women, even when the cause of infertility was a low sperm count, which led to perceive infertility care as the "women's world".[15] Additionally, the midwives discussed sexual and reproductive rights for men as being women's partners rather than being men's own rights, and men's concerns about contraception are dependent upon his partner's choice to include him or not in contraceptive counselling.[17]

4.4. Men's SRH is nobody's responsibility

Different HCPs expressed their concern about men's sexual health as "no one's responsibility". The attitudes of midwives toward providing counselling to men were divided. Some were positive and found it a continuation of their current responsibilities that concerned women.[17] This opinion was shared by men of pregnant partners who expressed their trust and faith in midwives and saw them as the best ones to promote sexual health among men.[49] Other midwives were reluctant and expressed their difficulty in providing counselling to men. For them, the pregnancy is about the woman's body, and thus, man's participation was not evident.[17] Nurses also questioned if men's sexual health is their duty, especially if it included an emotional aspect. In their opinion, primary care

For peer review only - http://bmjopen.bmj.com/site/about/guidelines.xhtml

was not equipped to deal with sexual health problems; therefore, they often referred patients to other healthcare units.[16]

5. Sociopolitical factors

The outer layer of the model (Figure 4) discusses the social and political factors, including social and gender norms, which affect the healthcare system and the attitudes and behaviours of HCPs and users.

5.1. Social and gender norms

The literature described traditional social and gender norms as contributing to setting values for men that hinder their abilities to cope or seek help and affect their sexual health and wellbeing. For example, infertility was described as a "malfunction of manhood" that is faced by denial and changes how men perceive their masculinities. The absence of sperm was an identity question and a threat to men's masculinities.[15,27,32,69] Similarly, suffering the decline in sexual function associated with a diagnosis of prostate cancer was perceived as threatening to the male identity and therefore accompanied by feelings of inadequacy and not being a "real man".[28,79]

Furthermore, the attempts of men to conform to traditional masculinity norms affected their ability to talk about experiences of violence, especially if they were exposed to intimate partner violence.[29] Additionally, transmen had experiences of vulnerability during gynaecological examination or when they resumed menstrual bleeding after family planning treatment. This was perceived as stressful, humiliating and uncomfortable, as well as a reminder of a sex they "wanted to forget".[85]

These "threatened masculinities" were also reflected through men's health seeking behaviours. Men disregarded their sexual health, delayed admission of the problem and opted to distance themselves from seeking healthcare.[13] For example, young men had more difficulties to admit their SRH needs and to seek help as compared to young women.[5] Similarly, the midwives indicated that men only seek help when they have severe symptoms, while also noting that young men are increasingly attending STI testing and are more open to discuss sexual health.[17]

Men expressed increased social expectations on them to be more involved in healthcare during pregnancy and birth, which corresponded to personal willingness and desire to share responsibility for the security and support of their partners.[28,39,45,47] Men were also eager to participate in other reproductive healthcare services, such as infertility treatment and home abortion.[36,73,84] However, men were faced with barriers in their desire to participate and experienced "paddling upstream" to fulfil their involvement.[40]

The literature also discussed how social and gender norms affect the healthcare system being perceived as women-centred. Youth clinics, for example, were perceived as a place for the SRH of girls, which created a barrier for young men seeking healthcare.[5] Additionally, the social norms hindered HCPs talking about sexual health, especially when the patient is older than the HCP.[16] In turn, HCPs reinforced these social norms by supporting the traditional gender expectations of the woman as the primary infant caregiver and overlooking the importance of shared parenthood and including the man in infant care.[39,86]

Studies also described how social and gender norms affected the way healthcare deals with victimized men. The training and education the emergency departments offered in Sweden about caring for violence victims focuses only on women and children and not victimized men.[87] Similar experiences of the reinforcement of traditional gender positions by HCPs were perceived by men

For peer review only - http://bmjopen.bmj.com/site/about/guidelines.xhtml

subjected to intimate partner violence. These men felt alone since society did not acknowledge their experiences, and the HCPs expected them to embody traditional ideals of masculinities.[29]

5.2. Policies

Politics and policies were rarely discussed in the literature, but there were some mentions of the regulations and guidelines in SRHC, which have been discussed under point 4. The only mention of policy was in the context of gender-based violence. While most of the counties and emergency departments in Sweden had a policy about the care for victims of violence, these policies focus merely on women and children but not men or other groups.[87]

Discussion

In the previous section we reviewed, charted and synthesized the available literature in relation to the factors influencing men's experiences in SRHC. To summarize, the majority of the 68 reviewed papers discussed men's experiences in reproductive healthcare, mainly care related to infertility, pregnancy and birth. The literature lacked men's perspectives on contraception, including condom use and vasectomy. Regarding sexual healthcare, the available literature captured mainly STIs and HIV treatment and prevention but not men's experiences in other sexual health issues, such as impotence or gender-based violence. This focus on STIs and reproduction reflects the biomedical gaze of healthcare. Keeping topics like gender-based violence and sexual satisfaction, to a great extent, outside the focus of healthcare and health service research. The literature also lacked the perspectives of particular groups of men who might face different experiences in SRHC, such as transmen, indigenous, national minorities and men with functional variations. Furthermore, MSM were only mentioned in relation to HIV treatment and prevention. Similarly, migrants were the main focus in only two studies related to foreign-born MSM and HIV testing.

The literature indicated that men face difficulties to be included in reproductive healthcare, where they are mostly treated as an accompanying partner, receiving little attention. The knowledge and attitudes of HCPs were crucial for their ability to discuss men's SRH and also for men's experiences in SRHC. Furthermore, the literature rarely discussed healthcare organization and policies and how they affect men's health seeking behaviours and experiences in SRHC. Lastly, men's right to SRH is usually not stressed in the literature, unless it is related to a specific group of men, such as MSM and transmen.

While we presented the factors influencing men's experiences in SRHC in separate levels and the reviewed articles did not explicitly study the interaction between these levels, the theoretical framework still enables us to understand the interaction between these determinants. We presented some examples in the results of how these levels are linked and influence each other. The interaction between gender and social norms with the other determinants might be of special significance. For example, the literature described how traditional social and gender norms affect the attitudes and behaviours of HCPs. A clear example of this was how men were treated as an accompanying partner during healthcare visits related to infertility, antenatal care and birth. In many cases men are still not seen as an equal partner or as a primary caregiver for their new-borns, which likely influences the attitudes of HCPs toward men seeking antenatal care, in turn affecting men's experiences of those services negatively.[14,40]

While traditional gender norms and values of masculinities provide important pieces in explaining men's health seeking behaviours, a more comprehensive picture of men's experiences in SRHC is

For peer review only - http://bmjopen.bmj.com/site/about/guidelines.xhtml

needed. The literature showed other determinants of men's experiences in SRHC, including how the healthcare system is organized. It seems SRHC in the Nordic countries focuses mainly on women, while there is a lack of knowledge about men's SRH and no clear entry for men into SRHC. The healthcare system should adapt a gender-responsive approach that ensures accessible healthcare services for men and which through its approach addresses the impacts of gender norms on men, women and HCPs.[2,88]

To reach universal access to SRHC and gender equity, it is of importance to engage men in SRH and ensure that their needs are met.[89] Improving men's experiences in SRHC in the Nordic countries is not only important for improving men's SRH but also could enable men to strengthen their support of women's SRH and thus gender equality.[89,90] Meeting men's needs for SRHC could consequently decrease STIs, unintended pregnancies and improve parenting and family relationships.[9,21] Such specific focus on men in the SRHC organization to improve men's health and rights with the goal to contribute to gender equality will benefit both men and women.[91,92]

Strengths and limitations of this study

To the best of our knowledge, this review is the first to examine the experiences of men in SRHC in the Nordic countries. The review provides interesting and important information about these experiences, by organizing them in a theoretical framework that make it easier to understand and draw conclusions. However, the design of our study and our search terms are best suited to draw conclusions about men's experiences of SRHC rather than the determinants of SRHC utilization even though we reported both in this study.

Even though we developed and followed our search strategies thoroughly, the review has some limitations. The broad nature of the field and the wide variety of terms related to SRH make it difficult to assure the inclusion of all relevant literature. Additionally, due to the restricted time of the project and the limited funding we included only peer-reviewed literature in two databases, we did not register a review protocol prior to the study and no stakeholder consultation was conducted after performing this scoping review. However, we complemented the search with manually screening the reference lists of the identified literature. Another strength of this review was the use of a Nordic-specific database without restriction to language, which ensured an equal inclusion of the literature from other Nordic countries, even though most of the literature in this review was published in Sweden.

Furthermore, the adapted framework allowed us to use a relevant ecological lens on men's experiences in SRHC and to systematically identify and categorize the concepts discussed in the selected literature. However, the use of this framework might have caused us to overlook aspects of the research topic that fell outside the interest of this scoping review.

Finally, it is important to note that, when reporting results and discussing them, we choose to implicitly treat the Nordic countries as essentially similar. While we argue that this makes sense because of the actual similarities between these countries and their healthcare systems, we also acknowledge that this might obscure important differences between or within countries (e.g., relation to place of residence (rural vs. urban) or cultural differences).

For peer review only - http://bmjopen.bmj.com/site/about/guidelines.xhtml

Conclusion

Despite the uncontroversial importance of men's right to access SRHC on equal terms, the available literature indicated that SRH is mainly the domain of women and healthcare around men's SRH is not sufficiently prioritized. A more comprehensive picture of men's experiences in SRHC is needed.

There is a lack of knowledge about men's SRH and no clear entry for men into SRHC. This indicates the necessity for improvements in the medical education of HCPs and in health system interventions. Further research should examine the influence of policies and the healthcare organization on men's access and experiences in SRHC and explore the identified knowledge gaps of men's experiences in SRHC related to specific groups of men such as migrants, MSM and transmen and to specific SRH subject areas such as sexual function, contraceptive use and gender-based violence.

For peer review only

Author contributions

MB was the lead reviewer and author of the manuscript. AKH and JPS contributed to the identification and selection of articles and contributed to data interpretation. HB and KE contributed to data interpretation and provided comments on the manuscript. All authors agreed on the final version of the manuscript. AKH was the project leader.

Declaration of interests

The authors declare that they have no conflict of interest.

Funding

This study was supported by the Public Health Agency of Sweden (Folkhälsomyndigheten). The Public Health Agency of Sweden was not involved in the analysis, interpretation, writing or the decision to submit this paper for publication.

Data availability statement

Data sharing not applicable as no datasets generated and/or analysed for this study.

Ethical statements

Patient consent for publication

Not required.

Ethical approval

Ethical approval for this study was not required as it was a review and no participants were involved.

Word count

5365 words

For peer review only - http://bmjopen.bmj.com/site/about/guidelines.xhtml

For peer review only

For peer review only - http://bmjopen.bmj.com/site/about/guidelines.xhtml

References

1. Osika Friberg I, Krantz G, Määttä S, Järbrink K. Sex differences in health care consumption in Sweden: a register-based cross-sectional study. *Scandinavian journal of public health*. 2016;**44**(3):264–73.
2. Europe WHO. Strategy on the health and well-being of men in the WHO European Region. Regional Committee for Europe; 2018.
3. Nicolosi A, Buvat J, Glasser DB, Hartmann U, Laumann EO, Gingell C. Sexual behaviour, sexual dysfunctions and related help seeking patterns in middle-aged and elderly Europeans: the global study of sexual attitudes and behaviors. *World journal of urology*. 2006;**24**(4):423–28.
4. Folkhälsomyndigheten. Sexuell och reproduktiv hälsa och rättigheter i Sverige 2017 Stockholm2019. Available from: http://www.folkhalsomyndigheten.se.
5. Sollesnes R. Exploring issues that motivate contact with adolescent health. *Vård i Norden*. 2010;**30**(2):4–7.
6. FSUM [Föreningen för Sveriges ungdomsmottagningar]. Handbok för Sveriges ungdomsmotagningar. [Handbook of Sweden's youth clinics] 2018. Available from: http://www.fsum.nu/wp-content/uploads/2018/05/handbok_original_utskrift.pdf.
7. Danielsson M, Berglund T, Forsberg M, Larsson M, Rogala C, Tydén T. Sexual and reproductive health: Health in Sweden: The National Public Health Report 2012. Chapter 9. *Scandinavian journal of public health*. 2012;**40**(9_suppl):176–96.
8. Kato-Wallace J, Barker G, Sharafi L, Mora L, Lauro G. Adolescent boys and young men: engaging them as supporters of gender equality and health and understanding their vulnerabilities. *Washington, DC and New York: Promundo and UNFPA*. 2016.
9. World Health Organization. The health and well-being of men in the WHO European Region: better health through a gender approach: World Health Organization. Regional Office for Europe; 2018.
10. Brannstrom J, Svedhem Johansson V, Marrone G, Wendahl S, Yilmaz A, Blaxhult A, et al. Deficiencies in the health care system contribute to a high rate of late HIV diagnosis in Sweden. *HIV medicine*. 2016;**17**(6):425–35.
11. Gios L, Mirandola M, Sherriff N, Toskin I, Blondeel K, Dias S, et al. Being in the closet. correlates of Outness among MSM in 13 European cities. *Journal of homosexuality*. 2021;**68**(3):415–33.
12. Hearn J, Nordberg M, Andersson K, Balkmar D, Gottzén L, Klinth R, et al. Hegemonic masculinity and beyond: 40 years of research in Sweden. *Men and masculinities*. 2012;**15**(1):31–55.
13. Christianson M, Boman J, Essen B. "Men don't think that far" - Interviewing men in Sweden about chlamydia and HIV testing during pregnancy from a discursive masculinities construction perspective. *Sexual & reproductive healthcare : official journal of the Swedish Association of Midwives*. 2017;**12**:107–15.
14. Widarsson M, Kerstis B, Sundquist K, Engström G, Sarkadi A. Support needs of expectant mothers and fathers: a qualitative study. *The Journal of perinatal education*. 2012;**21**(1):36–44.
15. Johansson M, Hellstrom AL, Berg M. Severe male infertility after failed ICSI treatment--a phenomenological study of men's experiences. *Reproductive health*. 2011;**8**:4.
16. Klaeson K, Hovlin L, Guva H, Kjellsdotter A. Sexual health in primary health care - a qualitative study of nurses' experiences. *Journal of clinical nursing*. 2017;**26**(11-12):1545–54.
17. Grandahl M, Bodin M, Stern J. In everybody's interest but no one's assigned responsibility: midwives' thoughts and experiences of preventive work for men's sexual and reproductive health and rights within primary care. *BMC public health*. 2019;**19**(1):1423.
18. European institute for gender equality. Gender Equality Index 2020 [Available from: https://eige.europa.eu/gender-equality-index/2020/compare-countries.

For peer review only

For peer review only - http://bmjopen.bmj.com/site/about/guidelines.xhtml

19. Levy JK, Darmstadt GL, Ashby C, Quandt M, Halsey E, Nagar A, et al. Characteristics of successful programmes targeting gender inequality and restrictive gender norms for the health and wellbeing of children, adolescents, and young adults: a systematic review. *The Lancet Global Health*. 2020;**8**(2):e225–e36.
20. Gupta GR, Oomman N, Grown C, Conn K, Hawkes S, Shawar YR, et al. Gender equality and gender norms: framing the opportunities for health. *The Lancet*. 2019;**393**(10190):2550–62.
21. WHO Europe. Strategy on the health and well-being of men in the WHO European Region. Regional Committee for Europe; 2018.
22. White A, De Sousa B, De Visser R, Hogston R, Madsen S, Matara P, et al. The State of Men’s Health in Europe: extended report. *Brussels: European Commission Directorate-General for Health and Consumers*. 2011.
23. Folkhälsomyndigheten. Motiverande samtal med unga om sexuell hälsa. En kartläggande litteraturöversikt inom omraadet hivprevention samt sexuell och reproduktiv hälsa och rättigheter (SRHR). 2018.
24. Arksey H, O'Malley L. Scoping studies: towards a methodological framework. *International journal of social research methodology*. 2005;**8**(1):19–32.
25. Organization WH. Sexual health and its linkages to reproductive health: an operational approach. 2017.
26. Kilbourne AM, Switzer G, Hyman K, Crowley-Matoka M, Fine MJ. Advancing health disparities research within the health care system: a conceptual framework. *American journal of public health*. 2006;**96**(12):2113–21.
27. Mikkelsen AT, Madsen SA, Humaidan P. Psychological aspects of male fertility treatment. *Journal of advanced nursing*. 2013;**69**(9):1977–86.
28. Klaeson K, Sandell K, Bertero CM. Talking about sexuality: desire, virility, and intimacy in the context of prostate cancer associations. *American journal of men's health*. 2013;**7**(1):42–53.
29. Simmons J, Bruggemann AJ, Swahnberg K. Disclosing victimisation to healthcare professionals in Sweden: a constructivist grounded theory study of experiences among men exposed to interpersonal violence. *BMJ open*. 2016;**6**(6):e010847.
30. Vik A, Brekke M. Do patients consult their GP for sexual concerns? A cross sectional explorative study. *Scand J Prim Health Care*. 2017;**35**(4):373–78.
31. Micaux Obol C, Armuand GM, Rodriguez-Wallberg KA, Ahlgren J, Ljungman P, Wettergren L, et al. Oncologists and hematologists' perceptions of fertility-related communication - a nationwide survey. *Acta oncologica (Stockholm, Sweden)*. 2017;**56**(8):1103–10.
32. Armuand GM, Wettergren L, Rodriguez-Wallberg KA, Lampic C. Women more vulnerable than men when facing risk for treatment-induced infertility: a qualitative study of young adults newly diagnosed with cancer. *Acta oncologica (Stockholm, Sweden)*. 2015;**54**(2):243–52.
33. Johansson M, Thies-Lagergren L. Swedish fathers' experiences of childbirth in relation to maternal birth position: a mixed method study. *Women and birth : journal of the Australian College of Midwives*. 2015;**28**(4):e140–7.
34. Herder T, Agardh A. Navigating between rules and reality: a qualitative study of HIV positive MSM's experiences of communication at HIV clinics in Sweden about the rules of conduct and infectiousness. *AIDS care*. 2019;**31**(10):1304–10.
35. Oster I, Hedestig O, Johansson M, Klingstedt N, Lindh J. Sharing experiences in a support group: men's talk during the radiotherapy period for prostate cancer. *Palliative & supportive care*. 2013;**11**(4):331–9.
36. Sylvest R, Furbringer JK, Schmidt L, Pinborg A. Infertile men's needs and assessment of fertility care. *Upsala journal of medical sciences*. 2016;**121**(4):276–82.
37. Andersson E, Christensson K, Hildingsson I. Parents' experiences and perceptions of group-based antenatal care in four clinics in Sweden. *Midwifery*. 2012;**28**(4):502–8.
38. Asenhed L, Kilstam J, Alehagen S, Baggens C. Becoming a father is an emotional roller coaster - an analysis of first-time fathers' blogs. *Journal of clinical nursing*. 2014;**23**(9-10):1309–17.

For peer review only - http://bmjopen.bmj.com/site/about/guidelines.xhtml

39. Johnsen H, Stenback P, Hallden BM, Crang Svalenius E, Persson EK. Nordic fathers' willingness to participate during pregnancy. *Journal of reproductive and infant psychology*. 2017;**35**(3):223–35.

40. Widarsson M, Engström G, Tydén T, Lundberg P, Hammar LM. 'Paddling upstream': Fathers' involvement during pregnancy as described by expectant fathers and mothers. *Journal of clinical nursing*. 2015;**24**(7-8):1059–68.

41. Johansson M, Rubertsson C, Rådestad I, Hildingsson I. Childbirth–an emotionally demanding experience for fathers. *Sexual & Reproductive Healthcare*. 2012;**3**(1):11–20.

42. Bäckström C, Wahn EH. Support during labour: first-time fathers' descriptions of requested and received support during the birth of their child. *Midwifery*. 2011;**27**(1):67–73.

43. Koert E, Malling GMH, Sylvest R, Krog MC, Kolte AM, Schmidt L, et al. Recurrent pregnancy loss: couples' perspectives on their need for treatment, support and follow up. *Human reproduction (Oxford, England)*. 2019;**34**(2):291–96.

44. Fabian H, Sarkadi A, Åhman A. Challenges and benefits of conducting parental classes in Sweden: Midwives' perspectives. *Sexual & Reproductive Healthcare*. 2015;**6**(4):236–42.

45. Premberg Å, Carlsson G, Hellström A-L, Berg M. First-time fathers' experiences of childbirth—A phenomenological study. *Midwifery*. 2011;**27**(6):848–53.

46. Hasman K, Kjaergaard H, Esbensen BA. Fathers' experience of childbirth when non-progressive labour occurs and augmentation is established. A qualitative study. *Sexual & Reproductive Healthcare*. 2014;**5**(2):69–73.

47. Persson EK, Fridlund B, Kvist LJ, Dykes A-K. Fathers' sense of security during the first postnatal week—A qualitative interview study in Sweden. *Midwifery*. 2012;**28**(5):e697–e704.

48. Grandahl M, Nevéus T, Dalianis T, Larsson M, Tydén T, Stenhammar C. 'I also want to be vaccinated!'–adolescent boys' awareness and thoughts, perceived benefits, information sources, and intention to be vaccinated against Human papillomavirus (HPV). *Human vaccines & immunotherapeutics*. 2019;**15**(7-8):1794–802.

49. Christianson M, Boman J, Essen B. 'Let men into the pregnancy'--men's perceptions about being tested for chlamydia and HIV during pregnancy. *Midwifery*. 2013;**29**(4):351–8.

50. Persson KI, Berglund T, Bergstrom J, Eriksson LE, Tikkanen R, Thorson A, et al. Motivators and barriers for HIV testing among men who have sex with men in Sweden. *Journal of clinical nursing*. 2016;**25**(23-24):3605–18.

51. Berg RC. Predictors of never testing for HIV among a national online sample of men who have sex with men in Norway. *Scandinavian Journal of Public Health*. 2013;**41**(4):398–404.

52. Stromdahl S, Liljeros F, Thorson AE, Persson KI, Forsberg BC. HIV testing and prevention among foreign-born Men Who have Sex with Men: an online survey from Sweden. *BMC public health*. 2017;**17**(1):139.

53. Makenzius M, Tyden T, Darj E, Larsson M. Autonomy and dependence - experiences of home abortion, contraception and prevention. *Scandinavian Journal of Caring Sciences*. 2013;**27**(3):569–79.

54. Marrone G, Mellgren A, Eriksson LE, Svedhem V. High Concordance between Self-Reported Adherence, Treatment Outcome and Satisfaction with Care Using a Nine-Item Health Questionnaire in InfCareHIV. *PloS one*. 2016;**11**(6):e0156916.

55. Bjornshagen V, Moseng BU, Ugreninov E. Who Do You Reach? A Norwegian Pilot Project on HIV Self-Testing that Targeted Men Who Have Sex with Men. *AIDS and behavior*. 2020;**24**(2):568–79.

56. Qvist T, Cowan SA, Graugaard C, Helleberg M. High linkage to care in a community-based rapid HIV testing and counseling project among men who have sex with men in Copenhagen. *Sexually transmitted diseases*. 2014;**41**(3):209–14.

57. Schmidt AJ, Hickson F, Weatherburn P, Marcus U. Comparison of the performance of STI screening services for gay and bisexual men across 40 European cities: results from the European MSM Internet Survey. *Sexually transmitted infections*. 2013;**89**(7):575–82.

For peer review only

For peer review only - http://bmjopen.bmj.com/site/about/guidelines.xhtml

58. Hoyos J, Mate T, Indave BI, Agusti C, Chanos S, Pichon F, et al. Knowledge, actual and potential use of HIV self-sampling testing kits among MSM recruited in eight European countries. *HIV medicine*. 2018;**19 Suppl 1**:27–33.

59. Stromdahl S, Hoijer J, Eriksen J. Uptake of peer-led venue-based HIV testing sites in Sweden aimed at men who have sex with men (MSM) and trans persons: a cross-sectional survey. *Sexually transmitted infections*. 2019;**95**(8):575–79.

60. Hildingsson I, Sjöling M. Fathers' experiences of support during pregnancy and the first year following childbirth–findings from a Swedish regional survey. *Journal of Men's Health*. 2011;**8**(4):258–66.

61. Schytt E, Bergstrom M. First-time fathers' expectations and experiences of childbirth in relation to age. *Midwifery*. 2014;**30**(1):82–8.

62. Hildingsson I, Cederlöf L, Widén S. Fathers' birth experience in relation to midwifery care. *Women and birth*. 2011;**24**(3):129–36.

63. Andersson E, Norman Å, Kanlinder C, Plantin L. What do expectant fathers expect of antenatal care in Sweden? A cross-sectional study. *Sexual & Reproductive Healthcare*. 2016;**9**:27–34.

64. Armuand GM, Rodriguez-Wallberg KA, Wettergren L, Ahlgren J, Enblad G, Hoglund M, et al. Sex differences in fertility-related information received by young adult cancer survivors. *Journal of clinical oncology : official journal of the American Society of Clinical Oncology*. 2012;**30**(17):2147–53.

65. Rasmusson EM, Plantin L, Elmerstig E. 'Did they think I would understand all that on my own?' A questionnaire study about sexuality with Swedish cancer patients. *European journal of cancer care*. 2013;**22**(3):361–9.

66. Fridriksson J, Gunseus K, Stattin P. Information on pros and cons of prostate-specific antigen testing to men prior to blood draw: a study from the National Prostate Cancer Register (NPCR) of Sweden. *Scandinavian journal of urology and nephrology*. 2012;**46**(5):326–31.

67. Bergengren O, Garmo H, Bratt O, Holmberg L, Johansson E, Bill-Axelson A. Satisfaction with Care Among Men with Localised Prostate Cancer: A Nationwide Population-based Study. *European urology oncology*. 2018;**1**(1):37–45.

68. Berglund A, Garmo H, Robinson D, Tishelman C, Holmberg L, Bratt O, et al. Differences according to socioeconomic status in the management and mortality in men with high risk prostate cancer. *European journal of cancer (Oxford, England : 1990)*. 2012;**48**(1):75–84.

69. Sylvest R, Koert E, Vittrup I, Birch Petersen K, Hvidman HW, Hald F, et al. Men's expectations and experiences of fertility awareness assessment and counseling. *Acta obstetricia et gynecologica Scandinavica*. 2018;**97**(12):1471–77.

70. Bodin M, Tyden T, Kall L, Larsson M. Can Reproductive Life Plan-based counselling increase men's fertility awareness? *Upsala journal of medical sciences*. 2018;**123**(4):255–63.

71. Holter H, Sandin-Bojo AK, Gejervall AL, Wikland M, Wilde-Larsson B, Bergh C. Patient-centred quality of care in an IVF programme evaluated by men and women. *Human reproduction (Oxford, England)*. 2014;**29**(12):2695–703.

72. Qvarnstrom A, Oscarsson MG. Experiences of and attitudes towards HIV/STI prevention among holidaymaking men who have sex with men living in Sweden: a cross-sectional Internet survey. *Scand J Public Health*. 2015;**43**(5):490–6.

73. Kero A, Lalos A, Wulff M. Home abortion–experiences of male involvement. *The European Journal of Contraception & Reproductive Health Care*. 2010;**15**(4):264–70.

74. Åhman A, Lindgren P, Sarkadi A. Facts first, then reaction—Expectant fathers' experiences of an ultrasound screening identifying soft markers. *Midwifery*. 2012;**28**(5):e667–e75.

75. Johansson M, Hildingsson I, Fenwick J. Important factors working to mediate Swedish fathers' experiences of a caesarean section. *Midwifery*. 2013;**29**(9):1041–49.

76. Jungmarker EB, Lindgren H, Hildingsson I. Playing second fiddle is okay–Swedish fathers' experiences of prenatal care. *The Journal of Midwifery & Women's Health*. 2010;**55**(5):421–29.

For peer review only - http://bmjopen.bmj.com/site/about/guidelines.xhtml

77. Andersson E, Small R. Fathers' satisfaction with two different models of antenatal care in Sweden - Findings from a quasi-experimental study. *Midwifery*. 2017;**50**:201–07.

78. Johansson M, Hildingsson I. Intrapartum care could be improved according to Swedish fathers: mode of birth matters for satisfaction. *Women and Birth*. 2013;**26**(3):195–201.

79. Schildmeijer K, Frykholm O, Kneck A, Ekstedt M. Not a Straight Line-Patients' Experiences of Prostate Cancer and Their Journey Through the Healthcare System. *Cancer nursing*. 2019;**42**(1):E36–e43.

80. Wibe T, Hellesø R, Varsi C, Ruland C, Ekstedt M. How does an online patient-nurse communication service meet the information needs of men with recently diagnosed testicular cancer? *International Scholarly Research Notices*. 2012;**2012**.

81. Thies-Lagergren L, Johansson M. Intrapartum midwifery care impact Swedish couple's birth experiences - A cross-sectional study. *Women and birth : journal of the Australian College of Midwives*. 2019;**32**(3):213–20.

82. Johansson M, Hildingsson I, Fenwick J. 'As long as they are safe–Birth mode does not matter'Swedish fathers' experiences of decision-making around caesarean section. *Women and Birth*. 2014;**27**(3):208–13.

83. Holter H, Gejervall AL, Borg K, Sandin-Bojo AK, Wikland M, Wilde-Larsson B, et al. In vitro fertilization healthcare professionals generally underestimate patients' satisfaction with quality of care. *Acta obstetricia et gynecologica Scandinavica*. 2017;**96**(3):302–12.

84. Makenzius M, Tyden T, Darj E, Larsson M. Women and men's satisfaction with care related to induced abortion. *The European journal of contraception & reproductive health care : the official journal of the European Society of Contraception*. 2012;**17**(4):260–9.

85. Armuand G, Dhejne C, Olofsson JI, Rodriguez-Wallberg KA. Transgender men's experiences of fertility preservation: a qualitative study. *Human reproduction (Oxford, England)*. 2017;**32**(2):383–90.

86. Erlandsson K, Häggström-Nordin E. Prenatal parental education from the perspective of fathers with experience as primary caregiver immediately following birth: a phenomenographic study. *The journal of perinatal education*. 2010;**19**(1):19.

87. Linnarsson JR, Benzein E, Årestedt K, Erlingsson C. Preparedness to care for victims of violence and their families in emergency departments. *Emergency medicine journal*. 2013;**30**(3):198–201.

88. Kågesten A, Chandra-Mouli V. Gender-transformative programmes: implications for research and action. *The Lancet Global Health*. 2020;**8**(2):e159–e60.

89. Starrs AM, Ezeh AC, Barker G, Basu A, Bertrand JT, Blum R, et al. Accelerate progress—sexual and reproductive health and rights for all: report of the Guttmacher–Lancet Commission. *The Lancet*. 2018;**391**(10140):2642–92.

90. Dahl S, Sonnerby C, Järvklo N. Män och jämställdhet; Betänkande av Utredningen om män och jämställdhet. Utredningar SO; 2014. Report No.: SOU 2014:6.

91. Ruane-McAteer E, Gillespie K, Amin A, Aventin Á, Robinson M, Hanratty J, et al. Gender-transformative programming with men and boys to improve sexual and reproductive health and rights: a systematic review of intervention studies. *BMJ global health*. 2020;**5**(10):e002997.

92. Bustamante-Forest R, Giarratano G. Changing men's involvement in reproductive health and family planning. *The Nursing Clinics of North America*. 2004;**39**(2):301–18.

For peer review only

For peer review only - http://bmjopen.bmj.com/site/about/guidelines.xhtml

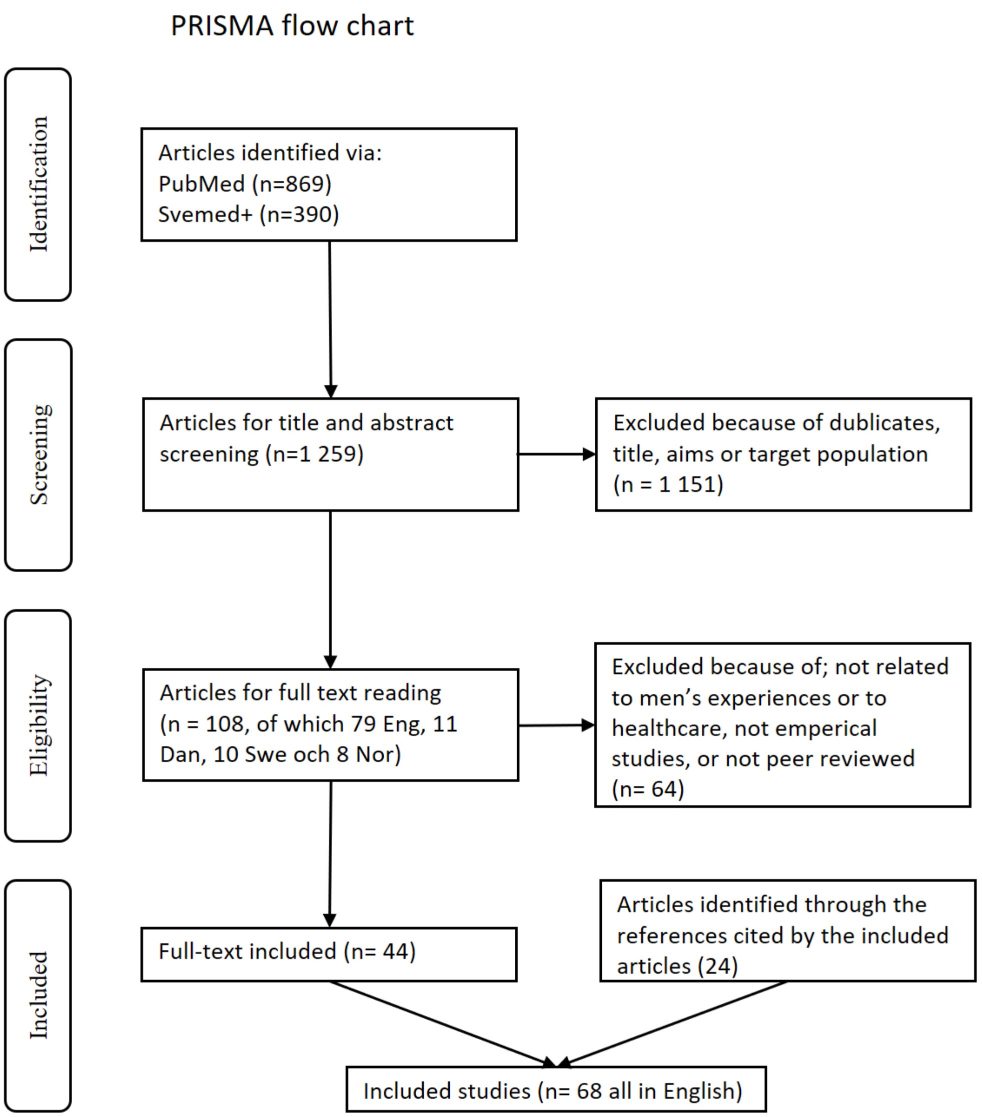

Figure 1: PRISMA flow chart on search results of men's experiences in sexual and reproductive healthcare in the Nordic countries.

512x579mm (57 x 57 DPI)

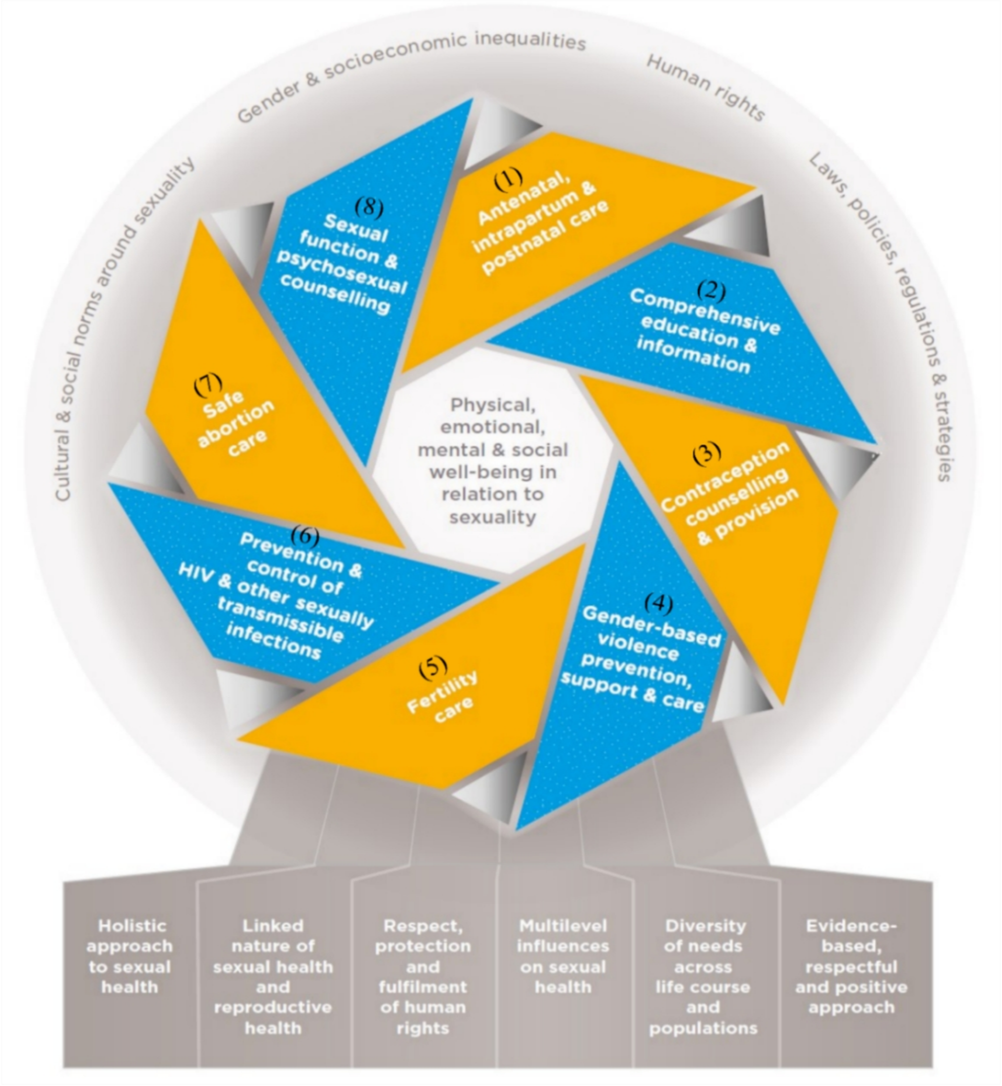

Figure 2: Framework for operationalizing sexual health and its linkages to reproductive health (from "Sexual health and its linkages to reproductive health: an operational approach").24 The intertwined blue and orange ribbons represent sexual health and reproductive health, respectively.

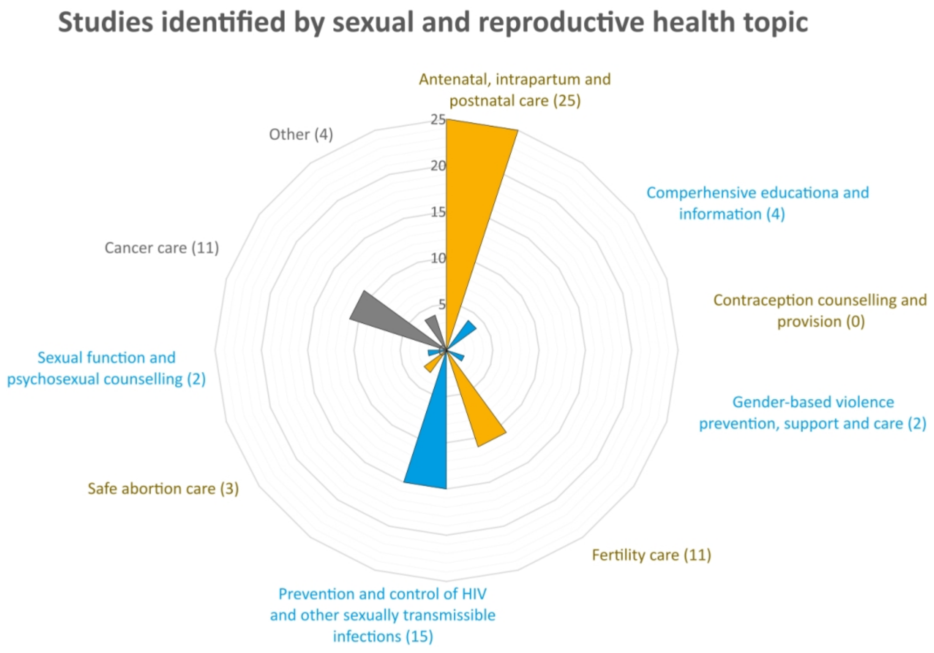

Figure 3: Men's experiences in sexual and reproductive healthcare in the Nordic countries. Number of studies identified grouped by sexual and reproductive health topics.

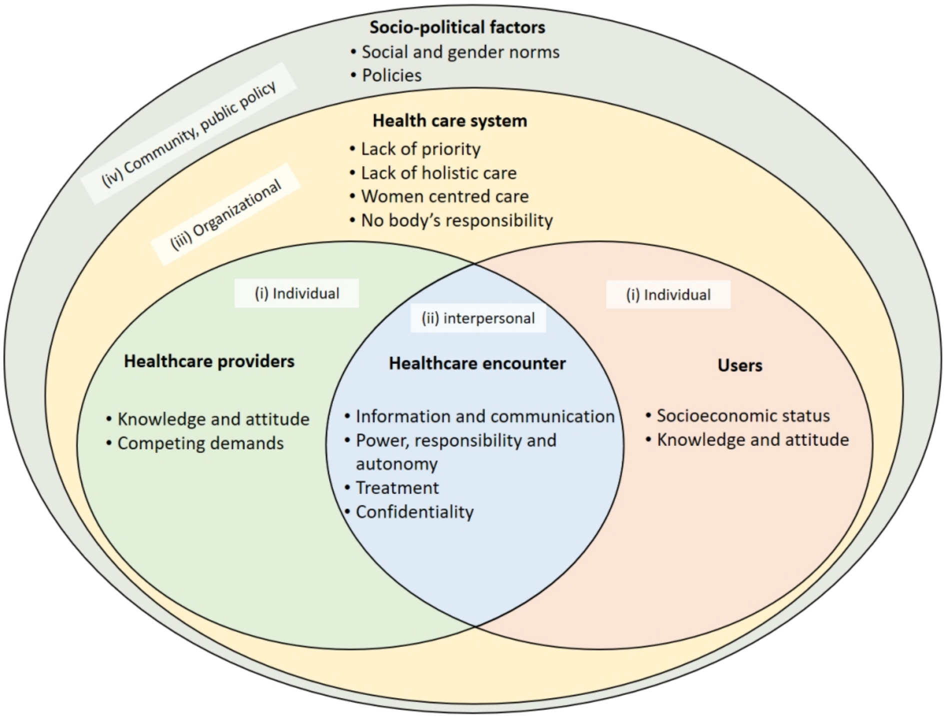

Figure 4: Theoretical framework for analysis of men's experiences in sexual and reproductive healthcare, adapted from Kilbourne et al.25

Appendix 1: Search terms

1) Pubmed/Medline

Search	Search terms	Nr. Of articles
**#1<br>Sexual and reproductive health**	(Sexual Health[mesh] OR “Sexual health” OR Reproductive Health[mesh] OR “Reproductive health” OR<br>“Sexual and reproductive health” OR “Sexual and reproductive health and rights” OR SRHR OR<br><br>"sexual function" OR "sexual functions" OR "sexual dysfunction" OR “sexual dysfunctions" OR<br>erectile dysfunction[mesh] OR “erectile dysfunction” OR sexual satisfaction OR sex offenses[mesh] OR sexual violence OR gender-based violence[mesh] OR gender-based violence OR<br><br>family planning services[mesh] OR "Family Planning" OR contraceptive agents[mesh] OR contraceptive devices[mesh] OR “contraceptive” OR “contraceptives” OR Condom[mesh] OR condom OR condoms OR Infertility[mesh] OR infertility OR fertility[mesh] OR fertility OR prostatic neoplasms[mesh] OR “Prostate cancer” OR<br><br>Genital Diseases, Male[mesh] OR sexually transmitted diseases[mesh] OR “Sexually transmitted infections” OR STIs OR “Sexually transmitted diseases” OR STDs OR chlamydia[mesh] OR Chlamydia OR Gonorrhoea[mesh] OR gonorrhea OR gonorrhoeae OR Syphilis[mesh] OR Syphilis OR Trichomonas Infections[mesh] OR Trichomoniasis OR Herpes Genitalis[mesh] OR "herpes genitalis”[tiab] OR Papillomavirus Infections[mesh] OR “Papillomavirus” OR Condylomata Acuminata[mesh] OR “Genital warts” OR HIV Infections[mesh])<br>AND	1 034 552
**#2<br>men**	(men[mesh] OR Men OR man OR men’s OR Male[mesh] OR male OR masculinity[mesh] OR masculinity OR Men's Health[mesh] OR Homosexuality, Male[mesh] OR “MSM” OR “men having sex with men”[TIAB] OR “men who have sex with men”[TIAB] OR “men who have sex with other men”[TIAB] OR<br>((transgender*[TIAB] OR transgender persons[MH] OR transsexual*[TIAB]) AND man) OR transman[TIAB] OR “trans men”[TIAB] OR transmen[TIAB]) AND	558 975
**#3<br>healthcare**	(Health Services Accessibility[mesh] OR Health Facilities[mesh] OR community health services[mesh] OR health services[mesh] OR health services research[mesh] OR delivery of health care[mesh] OR preventive health services[mesh] OR health services needs and demand[MH] OR quality of health care[majr:noexp] OR “Health care providers” OR<br><br>“Health care”[tiab] OR “Health services”[tiab] OR “Healthcare”[tiab] OR “health-care”[tiab] OR clinic[tiab] OR hospital[tiab] OR “primary care”[tiab]) AND	121 596
**#4 experiences**	(Professional-Patient Relations[mesh] OR Attitude of Health Personnel[mesh] OR Patient Satisfaction[mesh] OR Healthcare Disparities[mesh] OR patient acceptance of health care[MH] OR health care evaluation mechanisms[mesh] OR<br><br>“perception”[tiab] OR “perceptions”[tiab] OR “perceive”[tiab] OR “perceived”[tiab] OR “satisfaction”[tiab] OR “expectation”[tiab] OR “expectations”[tiab] OR “experience”[tiab] OR “experiences”[tiab] OR “evaluation”[tiab] OR “assessment”[tiab] OR “quality”[tiab] OR “trust”[tiab] OR “Shame”[tiab] OR “stigma”[tiab]) AND	93 209
**#5<br>Empirical studies**	(Epidemiologic Research Design[mesh] OR Qualitative Research[mesh] OR empirical research[mesh] OR<br>Surveys and Questionnaires[mesh] OR survey OR surveys OR questionnaires OR questionnaire OR Empirical OR Cross-Sectional Studies OR Cohort OR Case-Control OR Observational OR Registries OR analysis OR	79 428

	Clinical Trials OR Meta-Analysis OR "meta analysis" OR "systematic review" OR "scoping review" OR "literature review" OR "review of literature" OR<br><br>Qualitative OR "Grounded Theory" OR Interviews as Topic[mesh] OR "interviews" OR "interview" OR focus groups[mesh] OR "Focus group" OR "focus groups" OR themes[tiab]) AND	
**#6**<br>**Nordic countries**	(Scandinavian and Nordic Countries[mesh] OR Scandinavian[tiab] OR Denmark[tiab] OR Danish[tiab] OR Finland[tiab] OR Finnish[tiab] OR Norway[tiab] OR Norwegian[tiab] OR Sweden[tiab] OR Swedish[tiab] OR Iceland[tiab] OR Icelandic[tiab] OR Greenland[tiab])	1892

Search results 13th Mai 2020: 1892 articles

Published from 1st January 2010: 896 articles

2) Svemed+

(Sexual health[mesh] OR Reproductive health[mesh] OR sexually transmitted diseases[mesh] OR hiv infections[mesh] OR contraceptive agents[mesh] OR contraceptive devices[mesh] OR infertility[mesh] OR prostatic neoplasms[mesh] OR sex offences[mesh] OR erectile dysfunction[mesh]) AND
(men[mesh] OR male[mesh] OR masculinity[mesh]) AND
(Professional-Patient Relations[mesh] OR Attitude of Health Personnel[mesh] OR Patient Satisfaction[mesh] OR Healthcare[mesh] Disparities[mesh] OR patient acceptance of health care[mesh] OR health care evaluation mechanisms[mesh] OR Health Facilities[mesh] OR community health services[mesh] OR health services[mesh] OR preventive health services[mesh])

Search results 26th April 2020: 1696 articles

Published from 1st January 2010: 390 articles

For peer review only

Appendix 2:

list of included articles on men's experiences in sexual and reproductive healthcare in the Nordic countries

Authors (year)	SRH subject	Country	Study design	Aim of the study	Discussed concepts
**Åhman et al. (2012)** [74]	1	Sweden	Qualitative (interviews) 17 m	To explore men's expectations of routine ultrasound and experiences when soft markers were discovered.	Information, policy, organization of care, waiting times, satisfaction, control, autonomy, Support, power and responsibility
**Andersson et al. (2012)** [37]	1	Sweden	Qualitative (interviews) 20 k, 8 m	To investigate parents' experiences and perceptions of group antenatal care in different antenatal clinics in Sweden	Knowledge about the services, continuity of care, users' gender, information, hcps' knowledge/competence, Engaging men
**Andersson et al. (2016)** [63]	1	Sweden	Quasi randomized study (survey) 627 m	To identify expectant fathers' expectations regarding the content of antenatal care during pregnancy and to examine associations between expectations and social factors	Information, regard, engaging men, users' age
**Andersson et al. (2017)** [77]	1	Sweden	Quasi randomized study (survey) 239 m	To compare the experiences of fathers with two different models of antenatal care, group based antenatal care and standard antenatal care	Information, satisfaction, support, autonomy
**Armuand et al. (2012)** [64]	5, cancer	Sweden	Cross-sectional (survey) 328 k, 156 m	To investigate cancer survivors' perception of fertility-related information and use of FP options in connection with cancer treatment during reproductive age. An additional aim was to investigate the relationships between receiving fertility-related information and sociodemographic factors, diagnosis, and a pretreatment desire for children.	Information, users' age
**Armuand et al. (2015)** [32]	5, cancer	Sweden	Qualitative (interviews) 11 k, 10 m	To investigate newly diagnosed cancer patients' experiences of fertility-related communication and their reasoning about the risk of future infertility.	Information, satisfaction, autonomy, time constrain, No body's responsibility, vulnerability, masculinity
**Armuand et al. (2017)** [85]	5	Sweden	Qualitative (interviews) 15 m	To evaluate how transgender men experienced FP aimed at oocyte cryopreservation in a pilot program, which was developed within an established university hospital-based FP program.	Referral possibility, waiting times, vulnerability, masculinity, satisfaction
**Åsenhed et al. (2014)** [38]	1	Sweden	Qualitative (internet bloggs) 11 m	To identify and describe the process of fatherhood during the partner's pregnancy among expectant, first-time fathers.	Women centred care, engaging men, users' gender, willingness to talk
**Bäckström et al. (2011)** [42]	1	Sweden	Qualitative (interviews) 10 m	To explore how first-time fathers describe requested and received support during a normal birth.	Communication, trust, support, engaging men, autonomy, users' as person

For peer review only - http://bmjopen.bmj.com/site/about/guidelines.xhtml

**Berg (2013)** [51]	6	Norway	Cross-sectional (survey) 2011 m	To identify prevalence of, and factors that are associated with, HIV testing	Users' age, users' education, users' knowledge, knowledge about the services, users' attitude, users' sexuality, confidentiality, regard, holistic care
**Bergengren et al. (2018)** [67]	Cancer	Sweden	Register based cross-sectional (survey) 1 288 m	To investigate overall satisfaction with care (OSC) and factors associated with OSC among men with low-risk prostate cancer (PC)	Satisfaction, autonomy, information, hcps' profession, users' age, users' education, waiting times, health outcome
**Berglund et al. (2012)** [68]	Cancer	Sweden	Population based cohort 17 522 m	To examine possible associations between socioeconomic status, metastatic work-up, treatment and mortality in patients with high risk pca managed in Sweden	Users' SES, health outcome
**Bjornshagen et al. (2020)** [55]	6	Norway	Cross-sectional (survey) 849 m	To illustrate who might benefit from HIV self-testing, by describing the characteristics of MSM who took an interest in the GLHN pilot project	Confidentiality, anonymity, health seeking behaviours, users' sexuality, users' age
**Bodin et al. (2018)** [70]	5	Sweden	Randomised controlled study (structured interviews) 201 m	To evaluate if Reproductive Life Plan (RLP)-based counselling during a sexual health visit could increase men's fertility awareness	Users' knowledge, information, satisfaction
**Brannstrom et al. (2016)** [10]	6	Sweden	Cross-sectional (survey) 372 m, 203 w	To identify factors in HIV-infected patients and the health care system which contribute to late diagnosis.	Users' age, users' ethnicity, health outcome, users' sexuality, users' gender
**Christianson et al. (2013)** [49]	6	Sweden	Qualitative (interviews) 20 m	To investigate how to prevent transmission of HIV and CT from a gender perspective by exploring whether screening of men during pregnancy may be an innovative way to reach men, to increase detection, and to avoid the present gendered responsibility.	Risk perception, engaging men, women centred healthcare, information, knowledge about the services, social norms, No body's responsibility, users' attitude
**Christianson et al. (2017)** [13]	6	Sweden	Qualitative (interviews) 20 m	To discursively explore expectant fathers' perceptions of chlamydia and HIV, and their masculinity constructions about testing, and explored how they talked about their potential resistance towards testing and their pre-test emotions.	Vulnerability, masculinity, health seeking behaviour, users' attitude/feelings
**Erlandsson and Häggström-Nordin (2010)** [86]	1, 2	Sweden	Qualitative (interviews) 15 m	To capture fathers' conceptions of parental education topics, illuminated by their experiences as primary caregiver of their child immediately following birth.	Role of men, engaging men, social norms
**Fabian et al. (2015)** [44]	1, 2	Sweden	Qualitative (interviews) 26 midwives	To explore antenatal care midwives' experiences and thoughts about the parental class activities provided during pregnancy.	Holistic care, clinical training, hcps' competence, Users' social capital, engaging men, users' gender

For peer review only - http://bmjopen.bmj.com/site/about/guidelines.xhtml

**Fridriksson et al. (2012)** [66]	2, cancer	Sweden	Register based cross-sectional (survey)<br>1 621 m	To assess the proportion of men subsequently diagnosed with prostate cancer who had received information prior to blood draw	Information, knowledge about the services
**Grandahl and Small (2019)** [17]	Other	Sweden	Qualitative (interviews)<br>22 midwives	To explore the thoughts and experiences of midwives working in the primary care setting concerning their preventive work for men's sexual and reproductive health and rights	No body's responsibility, hcps' attitude, Clinical training, hcps' knowledge, health seeking behaviour, organization of care, time constrains, women centred care, engaging men
**Grandahl et al. (2109)** [48]	6	Sweden	Qualitative (interviews) 33 m	To investigates boys' awareness and thoughts about human papillomavirus (HPV) and HPV vaccination, perceived benefits of vaccinating men, information sources and intention to be vaccinated against HPV	Users' attitude, willingness to participate, risk perception, information
**Hasman et al. (2014)** [46]	1	Denmark	Qualitative (interviews)<br>10 m	To describe how fathers experienced childbirth when nonprogressive labour occurred and augmentation was established.	Information, engaging men, role of men, control, hcps' competence, , security
**Herder and Agardh (2019)** [34]	6	Sweden	Qualitative (interviews)<br>10 m	To explore experiences and perceptions regarding communication about infectiousness and the rules of conduct with clinical staff at HIV clinics among MSM living with HIV in Sweden	Hcps' attitude, regard, trust, hcps' knowledge, security, , information
**Hildingsson and Sjöling (2011)** [60]	1	Sweden	Prospective longitudinal study (survey)<br>655 m	To describe personal and professional sources of support used by prospective and new fathers and to study factors associated with fathers having no support from anyone in mid-pregnancy.	Support, engaging men, users' social capital, willingness to participate, satisfaction
**Hildingsson et al. (2011)** [62]	1	Sweden	Cross-sectional (survey)<br>595 m	To identify the proportion of fathers having a positive experience of a normal birth and to explore factors related to midwifery care that were associated with a positive experience.	Satisfaction, support, information, users' age, users' ethnicity, users' education
**Holter et al. (2014)** [71]	5	Sweden	Cross-sectional (survey)<br>292 m,363 k	To investigate whether men and women differ in their evaluations of the importance of different aspects of quality of care, when measured by the use of the validated QPP-IVF instrument and to investigate if any baseline characteristics influenced the scores of subjective importance	Continuity of care, information, satisfaction
**Holter et al. (2017)** [83]	5	Sweden	Cross-sectional (survey)<br>268 IVF staff, 1435 m, 1863 k	To compare IVF healthcare professionals' estimates with patients' actual experiences of patient-centered quality of care measured with the QPP-IVF questionnaire, and investigate if certain factors influenced the IVF professionals' perceptions and IVF patients' experience of quality of care.	Satisfaction, continuity of care, autonomy, easy access, users' education, public vs. Private

For peer review only - http://bmjopen.bmj.com/site/about/guidelines.xhtml

**Hoyos et al. (2018)** [58]	6	Multinational	Cross-sectional (survey) 8 226 m (397 Danish)	To describe the knowledge about the existence as well as actual and potential use of self-sampling testing and to assess the acceptability of different result communication methods as well as the preferred sampling method among MSM recruited online in eight European countries.	Knowledge about the services, acceptability, communication
**Johansson and Hildingsson (2013)** [78]	1	Sweden	Cross-sectional (survey) 827 m	To explore Swedish fathers' intrapartum care quality experiences, with a specific focus on care deficiencies in relation to birth mode. A secondary aim was to explore which issues of quality that contributed most to dissatisfaction with the overall assessment of the care.	Satisfaction, support, autonomy, nature of the problem, information
**Johansson and Thies-Lagergren (2015)** [33]	1	Sweden	Mixed methods (survey, free texts) 221 m	To investigate how maternal birth position during second stage of labour may influence fathers' experience of childbirth	Satisfaction, nature of the problem, power, security, engaging men, support, willingness to participate, hcps' attitude, information, hcps' knowledge/competence, continuity of care, trust
**Johansson et al. (2011)** [15]	5	Sweden	Qualitative (interviews) 8 m	To describe men's experiences of obstructive azoospermia infertility	Vulnerability, masculinity, women centred care, engaging men
**Johansson et al. (2012)** [41]	1	Sweden	Mixed methods (survey, free texts) 827 m	To explore Swedish fathers' birth experiences, and factors associated with a less-positive birth experience.	Satisfaction, nature of the problem, users' education, hcps' competence, support, information, regard, autonomy, engaging men
**Johansson et al. (2013)** [75]	1	Sweden	Qualitative (interviews) 22 m	To describe and explore fathers' experiences of their partner's caesarean section birth.	Nature of the problem, information, hcps' Attitude, support, satisfaction, hcps' competence, control
**Johansson et al. (2014)** [82]	1	Sweden	Qualitative (interviews) 21 m	To explore and describe Swedish fathers' beliefs and attitudes around the decision for a caesarean section.	Shared responsibility, trust, information, nature of the problem
**Johnsen et al. (2017)** [39]	1	Multinational	Qualitative (interviews) 31 m Sweden, 8 m Denmark, 5 m Finland	To illuminate expectant first-time fathers' experiences of participation during pregnancy in three Nordic countries.	Willingness to participate, engaging men, social norms, responsibility
**Jungmarker et al. (2010)** [76]	1	Sweden	Cohort study (survey) 827 m	To describe expectant fathers' experiences of and involvement in prenatal care in Sweden.	Willingness to participate, satisfaction, holistic care, information, support, engaging men
**Kero et al. (2010)** [73]	7	Sweden	Qualitative (interviews) 23 couples	To gain knowledge about the male partner's experience of being present during an induced home abortion	Willingness to participate, autonomy, information, engaging men

For peer review only - http://bmjopen.bmj.com/site/about/guidelines.xhtml

**Klaeson et al. (2013)** [28]	8, cancer	Sweden	Qualitative (fgds) 19 m	To explore how men diagnosed with prostate cancer before the age of 65 years, in all stages, experienced and talked about changes in their sexuality due to cancer as a subgroup in the society	Vulnerability, masculinity, nature of the problem, social norms, Willingness to talk, hcps' gender, continuity of care, information, support
**Klaeson et al. (2017)** [16]	Other	Sweden	Qualitative (interviews) 9 nurses	To illuminate nurses' experiences and opportunities to discuss sexual health with patients in primary health care	Social norms, hcps' attitude, clinical training, organization of care, no body's responsibility, users' gender, clinical training, holistic care, users' age
**Koert et al. (2019)** [43]	1	Denmark	Qualitative (interviews) 11 couples	What do couples referred to or attending a Recurrent Pregnancy Loss clinic believe they need in terms of treatment, support and follow up?	Holistic care, regard, engaging men , information, continuity of care, referral possibility, organization of care
**Linnarsson et al. (2013)** [87]	4	Sweden	Cross-sectional (survey) 46 emergency departments	To describe the preparedness to provide care for victims of violence and their families in emergency departments (eds) in Sweden.	Women centred care, policy, organization of care, clinical training
**Makenzius et al. (2012)** [84]	7	Sweden	Cross-sectional (survey) 590 m, 798 k	To investigate satisfaction with abortion care among women and their male partners, and to identify factors associated with high overall care satisfaction	Satisfaction, regard, information, willingness to participate
**Makenzius et al. (2013)** [53]	7	Sweden	Qualitative (interviews) 24 k, 13 m	To explore women's and men's experiences and needs in relation to an induced medical abortion that involves carrying out the final treatment at home and to elicit their views on contraception and prevention of future unwanted pregnancies.	Autonomy, responsibility, continuity of care, privacy, control, information, regard, satisfaction, women centred care
**Marrone et al. (2016)** [54]	6	Sweden	Register based cross-sectional (survey) 1896 m, 950 k	To evaluate the Health Questionnaire and identify the main determinants of adherence	Satisfaction, autonomy, users' gender, users' ethnicity, health outcome
**Micaux Obol et al. (2017)** [31]	5, cancer	Sweden	Nationwide cross-sectional (survey) 329 oncologists and haematologists	To investigate the practice behaviors, attitudes, confidence in knowledge and perceived barriers to discussing fertility issues among physicians in cancer care and to identify factors related to physicians' practice behaviors regarding discussions about treatment-related fertility risks with female and male patients of reproductive age.	Information, users' age, no body's responsibility, referral possibility, workload, organization of care, hcps' knowledge
**Mikkelsen et al. (2013)** [27]	5	Denmark	Cross-sectional (survey) 210 m	To gain further knowledge about the experiences of infertile men for whom intracytoplasmic sperm injection treatment was the only way to establish fatherhood and to explore the psychological needs of the infertile man, focusing on communication in the clinic	Vulnerability, masculinity, engaging men, information , hcps' gender

For peer review only - http://bmjopen.bmj.com/site/about/guidelines.xhtml

**Oster et al. (2013)** [35]	Cancer	Sweden	Qualitative (conversational support group sessions) 9 m	To describe the shared experiences in a conversational support group of men with prostate cancer during a course of radiotherapy.	Satisfaction, nature of the problem, autonomy, waiting times, information, hcps' attitude, support, regard
**Persson et al. (2012)** [47]	1	Sweden	Qualitative (fgds, interviews) 20 m	To explore and describe factors, which influence fathers' sense of security during the first postnatal week.	Willingness to participate, information, security, hcps' knowledge/competence, organization of care, engaging men, support, regard, follow up
**Persson et al. (2016)** [50]	6	Sweden	Cross-sectional (survey) 2 373 m	To explore motivators and barriers to HIV testing and to assess factors associated with testing among MSM in the era of ART	Risk perception, users' knowledge, confidentiality, knowledge about the services, users' age, users' ethnicity, users' education
**Premberg et al. (2011)** [45]	1	Sweden	Qualitative (re-enactment interviews) 10 m	To describe fathers' experiences during childbirth	Information, willingness to participate, engaging men, support, autonomy
**Qvarnstrom and Oscarsson (2015)** [72]	6	Sweden	Cross-sectional (survey) 656 m	To describe experiences of and attitudes towards HIV/ STI prevention efforts prior to travel abroad among MSM and to investigate the kinds of prevention efforts that are desirable.	Availability of services, information, information
**Qvist et al. (2014)** [56]	6	Denmark	Cross-sectional (survey) 1 clinic	To evaluate a community based human immunodeficiency virus (HIV) testing program for its capacity to reach men who have sex with men (MSM) and successfully refer HIV-positive patients to treatment.	Easy access, follow up, referral possibility
**Rasmusson et al. (2013)** [65]	8, cancer	Sweden	Cross-sectional (survey) 54 k, 51 m	To investigate information about sexual effects of cancer on patients irrespective of age and diagnosis in terms of fertility, sexual desire and sexual function.	Information, users' gender, users' age
**Schildmeijer et al. (2019)** [79]	Cancer	Sweden	Qualitative (interviews) 14 m	1. Explore and describe how patients diagnosed with prostate cancer experience their journey through cancer care, by visualizing a typical patient journey and juxtaposing it with the SCP, and 2. Identify the patients' needs for support during the journey.	Information, waiting times, control, vulnerability, masculinity
**Schmidt et al. (2013)** [57]	6	Multinational	Cross-sectional (survey) 52430 m	To compare the performance of STI services used by MSM.	Knowledge about the services
**Schytt and Bergstrom (2014)** [61]	1	Sweden	Randomized controlled study (survey) 777 m	To investigate first-time fathers' expectations and experiences of childbirth and satisfaction with care in relation to paternal age.	Users' age, satisfaction, hcps' competence, support, trust

For peer review only - http://bmjopen.bmj.com/site/about/guidelines.xhtml

**Simmons et al. (2016)** [29]	4	Sweden	Qualitative (interviews) 12 m	To develop a theoretical model concerning male victims' processes of disclosing experiences of victimisation to healthcare professionals in Sweden.	Holistic care, fear, trust, confidentiality, masculinity, users' gender, hcps' gender, social norms, gender norms, support, regard
**Sollesnes (2010)** [5]	Other	Norway	Qualitative (fgds) 22 k, 10 m	To obtain insight to factors that can influence adolescent males and females' use of adolescent health clinics	Women centred care, help seeking behaviour, knowledge about the services, confidentiality, regard (patient as person)
**Stromdahl et al. (2017)** [52]	6	Sweden	Cross-sectional (survey) 244 m	To examine HIV-testing prevalence and uptake of HIV prevention interventions including different HIV-testing options among foreign-born MSM living in Sweden	Users' ethnicity, knowledge about the services, risk perception, easy access, anonymity, confidentiality
**Stromdahl et al. (2019)** [59]	6	Sweden	Cross-sectional (survey) 595 m	To evaluate the uptake of Testpoint (whether Testpoint achieved its aim of reaching MSM and trans persons, with a special focus on young and foreign born MSM)	Health seeking behaviour, users' ethnicity, users' age, knowledge about the services
**Sylvest et al. (2016)** [36]	5	Denmark	Qualitative (interviews) 10 m	To explore experience, expectations, needs, and assessment of fertility care among men with severe male-factor infertility.	Information, communication, waiting times, regard (patient as a person), willingness to participate, engaging men, holistic care
**Sylvest et al. (2018)** [69]	5	Denmark	Qualitative (interviews) 21 m	To explore men's expectations and experiences of fertility assessment and counseling through qualitative interviews conducted immediately before and some weeks after fertility counseling	Information, power, satisfaction, regard, masculinity, vulnerability
**Thies-Lagergren and Johansson (2019)** [81]	1	Sweden	Cross-sectional (survey) 209 couples	To describe and evaluate uniformity in couples' birth experience and experience of the quality of intrapartum midwifery care	Satisfaction, control, support,
**Vik and Brekke (2017)** [30]	Other	Norway	Cross-sectional (survey) 22 general practitioners 1 117 consultations	To shed some light upon how frequently and how Norwegian gps deal with concerns related to sexuality among their patients.	Prevalence of sexual problems presented to GP, hcps' competence, satisfaction
**Wibe et al. (2012)** [80]	2, cancer	Norway	Qualitative (online messages, interviews) 12 m	To explore how an online patient-nurse communication (OPNC) service meets the information needs of men with newly diagnosed testicular cancer	Information, support, control, communication, Waiting times
**Widarsson et al. (2012)** [14]	1	Sweden	Qualitative (fgds, interviews) 22 k, 10 m	To describe expectant mothers' and fathers' perceived needs of support during pregnancy	Holistic care, satisfaction, engaging men, women centred care, information
**Widarsson et al. (2015)** [40]	1	Sweden	Qualitative (fgds, interviews) 20 k, 10 m	To describe the perspectives of expectant mothers and fathers on fathers' involvement during pregnancy	Willingness to participate, engaging men, information, satisfaction

For peer review only - http://bmjopen.bmj.com/site/about/guidelines.xhtml

1. Antenatal, intrapartum and postnatal care
2. Comprehensive education and information
3. Contraception counselling and provision
4. Gender-based violence prevention, support and care
5. Fertility care
6. Prevention and control of HIV and other sexually transmissible infections
7. Safe abortion care
8. Sexual function and psychosexual counselling

For peer review only

For peer review only - http://bmjopen.bmj.com/site/about/guidelines.xhtml

Preferred Reporting Items for Systematic reviews and Meta-Analyses extension for Scoping Reviews (PRISMA-ScR) Checklist

SECTION	ITEM	PRISMA-ScR CHECKLIST ITEM	REPORTED ON PAGE #
**TITLE**			
Title	1	Identify the report as a scoping review.	Line 1,2
**ABSTRACT**			
Structured summary	2	Provide a structured summary that includes (as applicable): background, objectives, eligibility criteria, sources of evidence, charting methods, results, and conclusions that relate to the review questions and objectives.	Lines 22 to 43
**INTRODUCTION**			
Rationale	3	Describe the rationale for the review in the context of what is already known. Explain why the review questions/objectives lend themselves to a scoping review approach.	Lines 83 to 88
Objectives	4	Provide an explicit statement of the questions and objectives being addressed with reference to their key elements (e.g., population or participants, concepts, and context) or other relevant key elements used to conceptualize the review questions and/or objectives.	Lines 90,91 and 95 to 97
**METHODS**			
Protocol and registration	5	Indicate whether a review protocol exists; state if and where it can be accessed (e.g., a Web address); and if available, provide registration information, including the registration number.	Lines 493
Eligibility criteria	6	Specify characteristics of the sources of evidence used as eligibility criteria (e.g., years considered, language, and publication status), and provide a rationale.	Lines 102 to 105
Information sources*	7	Describe all information sources in the search (e.g., databases with dates of coverage and contact with authors to identify additional sources), as well as the date the most recent search was executed.	Lines 99 to 105 and appendix 1
Search	8	Present the full electronic search strategy for at least 1 database, including any limits used, such that it could be repeated.	Appendix 1
Selection of sources of evidence†	9	State the process for selecting sources of evidence (i.e., screening and eligibility) included in the scoping review.	Lines 109 to 112
Data charting process‡	10	Describe the methods of charting data from the included sources of evidence (e.g., calibrated forms or forms that have been tested by the team before their use, and whether data charting was done independently or in duplicate) and any processes for obtaining and confirming data from investigators.	Lines 114 to 118 and appendix 2
Data items	11	List and define all variables for which data were sought and any assumptions and simplifications made.	Appendix 2 last column “discussed concepts”
Critical appraisal of individual	12	If done, provide a rationale for conducting a critical appraisal of included sources of evidence; describe	NA

SECTION	ITEM	PRISMA-ScR CHECKLIST ITEM	REPORTED ON PAGE #
sources of evidence§		the methods used and how this information was used in any data synthesis (if appropriate).	
Synthesis of results	13	Describe the methods of handling and summarizing the data that were charted.	Lines 114 to 118
**RESULTS**			
Selection of sources of evidence	14	Give numbers of sources of evidence screened, assessed for eligibility, and included in the review, with reasons for exclusions at each stage, ideally using a flow diagram.	Figure 3
Characteristics of sources of evidence	15	For each source of evidence, present characteristics for which data were charted and provide the citations.	Appendix 2
Critical appraisal within sources of evidence	16	If done, present data on critical appraisal of included sources of evidence (see item 12).	NA
Results of individual sources of evidence	17	For each included source of evidence, present the relevant data that were charted that relate to the review questions and objectives.	Appendix 2
Synthesis of results	18	Summarize and/or present the charting results as they relate to the review questions and objectives.	Figure 4
**DISCUSSION**			
Summary of evidence	19	Summarize the main results (including an overview of concepts, themes, and types of evidence available), link to the review questions and objectives, and consider the relevance to key groups.	Lines 437 to 449
Limitations	20	Discuss the limitations of the scoping review process.	Lines 484 to 508
Conclusions	21	Provide a general interpretation of the results with respect to the review questions and objectives, as well as potential implications and/or next steps.	Lines 510 to 519
**FUNDING**			
Funding	22	Describe sources of funding for the included sources of evidence, as well as sources of funding for the scoping review. Describe the role of the funders of the scoping review.	Lines 527 to 529

JBI = Joanna Briggs Institute; PRISMA-ScR = Preferred Reporting Items for Systematic reviews and Meta-Analyses extension for Scoping Reviews.

* Where *sources of evidence* (see second footnote) are compiled from, such as bibliographic databases, social media platforms, and Web sites.

† A more inclusive/heterogeneous term used to account for the different types of evidence or data sources (e.g., quantitative and/or qualitative research, expert opinion, and policy documents) that may be eligible in a scoping review as opposed to only studies. This is not to be confused with *information sources* (see first footnote).

‡ The frameworks by Arksey and O'Malley (6) and Levac and colleagues (7) and the JBI guidance (4, 5) refer to the process of data extraction in a scoping review as data charting.

§ The process of systematically examining research evidence to assess its validity, results, and relevance before using it to inform a decision. This term is used for items 12 and 19 instead of "risk of bias" (which is more applicable to systematic reviews of interventions) to include and acknowledge the various sources of evidence that may be used in a scoping review (e.g., quantitative and/or qualitative research, expert opinion, and policy document).

From: Tricco AC, Lillie E, Zarin W, O'Brien KK, Colquhoun H, Levac D, et al. PRISMA Extension for Scoping Reviews (PRISMAScR): Checklist and Explanation. Ann Intern Med. 2018;169:467–473. doi: 10.7326/M18-0850.
